# Genome-Wide Identification and Function of Aquaporin Genes During Dormancy and Sprouting Periods of Kernel-Using Apricot (*Prunus armeniaca* L.)

**DOI:** 10.3389/fpls.2021.690040

**Published:** 2021-10-04

**Authors:** Shaofeng Li, Lin Wang, Yaoxiang Zhang, Gaopu Zhu, Xuchun Zhu, Yongxiu Xia, Jianbo Li, Xu Gao, Shaoli Wang, Jianhui Zhang, Ta-na Wuyun, Wenjuan Mo

**Affiliations:** ^1^State Key Laboratory of Tree Genetics and Breeding, Experimental Center of Forestry in North China, National Permanent Scientific Research Base for Warm Temperate Zone Forestry of Jiulong Mountain in Beijing, Chinese Academy of Forestry, Beijing, China; ^2^State Key Laboratory of Tree Genetics and Breeding, Non-timber Forestry Research and Development Center, Chinese Academy of Forestry, Zhengzhou, China; ^3^Department of Plant Sciences and Plant Pathology, Montana State University, Bozeman, MT, United States

**Keywords:** cold resistance, functional analysis, genome-wide analysis, aquaporin gene, kernel-using apricot (*P. armeniaca* L.)

## Abstract

Aquaporins (AQPs) are essential channel proteins that play a major role in plant growth and development, regulate plant water homeostasis, and transport uncharged solutes across biological membranes. In this study, 33 *AQP* genes were systematically identified from the kernel-using apricot (*Prunus armeniaca* L.) genome and divided into five subfamilies based on phylogenetic analyses. A total of 14 collinear blocks containing *AQP* genes between *P. armeniaca* and *Arabidopsis thaliana* were identified by synteny analysis, and 30 collinear blocks were identified between *P. armeniaca* and *P. persica.* Gene structure and conserved functional motif analyses indicated that the *PaAQPs* exhibit a conserved exon-intron pattern and that conserved motifs are present within members of each subfamily. Physiological mechanism prediction based on the aromatic/arginine selectivity filter, Froger’s positions, and three-dimensional (3D) protein model construction revealed marked differences in substrate specificity between the members of the five subfamilies of *PaAQPs*. Promoter analysis of the *PaAQP* genes for conserved regulatory elements suggested a greater abundance of cis-elements involved in light, hormone, and stress responses, which may reflect the differences in expression patterns of *PaAQPs* and their various functions associated with plant development and abiotic stress responses. Gene expression patterns of *PaAQP*s showed that *PaPIP1-3*, *PaPIP2-1*, and *PaTIP1-1* were highly expressed in flower buds during the dormancy and sprouting stages of *P. armeniaca.* A *PaAQP* coexpression network showed that *PaAQPs* were coexpressed with 14 cold resistance genes and with 16 cold stress-associated genes. The expression pattern of 70% of the *PaAQPs* coexpressed with cold stress resistance genes was consistent with the four periods [Physiological dormancy (PD), ecological dormancy (ED), sprouting period (SP), and germination stage (GS)] of flower buds of *P. armeniaca*. Detection of the transient expression of *GFP*-tagged PaPIP1-1, PaPIP2-3, PaSIP1-3, PaXIP1-2, PaNIP6-1, and PaTIP1-1 revealed that the fusion proteins localized to the plasma membrane. Predictions of an *A. thaliana* ortholog-based protein–protein interaction network indicated that PaAQP proteins had complex relationships with the cold tolerance pathway, PaNIP6-1 could interact with WRKY6, PaTIP1-1 could interact with TSPO, and PaPIP2-1 could interact with ATHATPLC1G. Interestingly, overexpression of *PaPIP1-3* and *PaTIP1-1* increased the cold tolerance of and protein accumulation in yeast. Compared with wild-type plants, *PaPIP1-3-* and *PaTIP1-1*-overexpressing (OE) *Arabidopsis* plants exhibited greater tolerance to cold stress, as evidenced by better growth and greater antioxidative enzyme activities. Overall, our study provides insights into the interaction networks, expression patterns, and functional analysis of *PaAQP* genes in *P. armeniaca* L. and contributes to the further functional characterization of *PaAQPs*.

## Introduction

Aquaporins (AQPs) are involved in water transport and facilitate the passage of other small solutes, such as CO_2_, boric acid, H_2_O_2_, glycerol, urea, and silicic acid, through cell membranes (Maurel et al., 2008, 2009). In plants, AQPs play a central role in maintaining the hydraulic conductivity balance and water homeostasis and are involved in the tolerance of abiotic stresses such as cold, drought, or salt stresses. In addition, the roles of AQPs in various growth processes, such as fruit ripening, petal and stomatal regulation, male fertility, seed germination, petal movement, and guard cell closure, have been documented (Heinen and Chaumont, 2009; Mitani-Ueno et al., 2011).

According to the cluster analysis of gene members, AQPs can be classified into seven subfamilies: small basic intrinsic proteins (SIPs), tonoplast intrinsic proteins (TIPs), plasma membrane intrinsic proteins (PIPs), uncategorized X intrinsic proteins (XIPs), nodulin26-like intrinsic proteins (NIPs), hybrid intrinsic proteins (HIPs), and GlpF-like intrinsic proteins (GIPs) (Chaumont et al., 2001; Kaldenhoff and Fischer, 2006; Maurel et al., 2015). Plant AQPs usually include four subfamilies: TIPs, PIPs, SIPs, and NIPs (Anderberg et al., 2012). Although XIP subfamily members are found in some dicots, such as common bean (Ariani and Gepts, 2015), poplar (Gupta and Sankararamakrishnan, 2009), potato (Venkatesh et al., 2013), and *Glycine max* (Cheng et al., 2013), they have not been found in monocots or certain dicots, such as *Arabidopsis thaliana* (Danielson and Johanson, 2008). GIP and HIP subfamily members have been reported in *Physcomitrella* (Danielson and Johanson, 2008) and *Selaginella* (Anderberg et al., 2012).

Recent studies have shown that AQP families are composed of a large number of genes in plants. For instance, there are 34 *AQPs* in *Oryza sativa* (Nguyen et al., 2013), 53 in Chinese cabbage (Tao et al., 2014), 66 in *Glycine max* (Zhang et al., 2013), 35 in *A. thaliana* (Johanson et al., 2001), 41 in common bean (Ariani and Gepts, 2015), 55 in *Populus trichocarpa* (Gupta and Sankararamakrishnan, 2009), and 31 in *Zea mays* (Chaumont et al., 2001). The structural features of AQPs are highly conserved and involve an α-helical bundle forming six transmembrane domains (TM1-TM6), which are connected by five loops (LA-LE). Two conserved Asn-Pro-Ala (NPA) motifs are located in loop B (LB) and loop E (LE) and are involved in transport selectivity (Bansal and Sankararamakrishnan, 2007; Kayum et al., 2017). The ar/R selectivity filter is formed by each residue from TM2 (H2), TM5 (H5), and two residues from LE (LE1 and LE2) and determines the substrate specificity (Hove and Bhave, 2011; Mitani-Ueno et al., 2011). Froger positions P1-P5 represent five conserved amino acid residues and discriminate between glycerol-transporting AQPs (aquaglyceroporins, GLPs) and water-conducting AQPs (Froger et al., 1998). NPA motifs, aromatic/arginine (ar/R) selectivity, and Froger’s position are crucial for the physiological functions of AQPs.

Kernel-using apricot (*P. armeniaca* L., syn. *Armeniaca vulgaris* Lam., also considered by some researchers to be *P. armeniaca* L. × *P. sibirica* L.) is an economically and ecologically important forest tree species in the three northern regions of China (northwestern China, northern China, and northeastern China). Because of its broad adaptability, strong resistance to drought, and easy management, it is one of the pioneer tree species used for ecological construction in mountainous areas and sandy lands, especially in western China. It is one of the six major nut-producing species in the world and is an important species for the production of woody oil and protein beverages. It can also contribute to the economies of poverty-stricken mountainous areas in China. The first high-quality apricot plant (Chuanzhihong) genome has been sequenced and released, laying a foundation for research on the molecular mechanisms underlying important agronomic characteristics of apricot (Jiang et al., 2019). Owing to its short dormancy period and early flowering period, kernel-using apricot (*P*. *armeniaca* L.) easily suffers from late frost damage, resulting in yield reduction or even no production. An effective strategy to solve the problem of a low and unstable yield of kernel-using apricot is to mine key genes related to flower bud dormancy and cold resistance, determine gene expression patterns during dormancy and sprouting periods (SPs), elucidate possible functional mechanisms and determine how the flowering period can be artificially manipulated.

Physiological dormancy (PD) (which is generally called endodormancy) is defined as the stage in which buds cannot burst even under optimal environmental conditions, while ecological dormancy (ED) (which is generally called ecodormancy) is the stage in which buds cannot burst because of lacking environmental factors (especially the temperature requirement accumulation for flower buds in ED periods is not enough). Endodormant buds, which cannot burst under preferred conditions due to internal physiological factors (such as embryos that are not mature physiologically, have a hard seed coat with poor permeability, or contain substances inhibiting germination) (Mitrakos and Diamantoglou, 2006; Gao et al., 2020), transition into the ecodormant (ecologically dormant) stage and acquire bud break capacity after exposure to a certain period of low temperature. When external environmental conditions (the temperature requirement accumulation for flower buds in ecologically dormant periods is enough) (Mazzitelli et al., 2007; Rohde and Bhalerao, 2007) are satisfied, the flower buds enter the SP and germination stage (GS), showing growth signs and sprouting, after which they can fully develop.

Cold stress can cause chilling injury in late spring, which obviously limits plant development and influences fruit yield in economic forests. Previous studies have shown that AQP family members play vital roles in different dormancy periods and lead to freezing tolerance and cold acclimation. For example, in *A. thaliana*, the expression of *AtPIP2-5* and *AtPIP2-6* is significantly upregulated by cold stress in roots and other parts of the plant (Alexandersson et al., 2010). The expression of rice *OsTIP1-1* is downregulated under cold stress conditions (Sakurai et al., 2005) but upregulated in response to water and salinity stress (Liu et al., 1994). Transgenic studies on *AQPs* have been conducted to examine their responses to abiotic stresses such as cold, drought, and salinity. *A. thaliana* plants overexpressing *AtPIP1;4* or *AtPIP2-5* exhibit greater tolerance to cold stress (Afzal et al., 2016). Compared with wild-type tobacco plants, transgenic tobacco plants overexpressing the wheat *TaAQP7* (*PIP2*) gene display improved cold tolerance (Zhou et al., 2012). Overexpression of *MusaPIP1-2* in transgenic banana plants enhances tolerance to cold and drought conditions (Shekhawat and Ganapathi, 2013). Although *AQPs* have been extensively studied in both dicots and monocots through genome-wide and functional analyses (Nguyen et al., 2013), our knowledge of *AQPs* in *P. armeniaca* is still limited, and the expression profile of *AQP* members in flower buds in the different dormancy and sprouting stages is largely unknown in this species.

In this study, the genome sequence of *P. armeniaca* (Longwangmao) and the sequences of known *AQPs* from other species (such as *A. thaliana* and *Prunus persica*) were used to identify the genes encoding AQP members. The phylogenetic relationships, chromosome distribution, conserved residues and elements, protein–protein interactions, and expression patterns of *AQPs* were investigated to identify their functions in cold stress in kernel-using apricot. These results provide valuable information for further studies on the molecular mechanism of *AQPs*, which will facilitate the selection of candidate genes for cold tolerance improvement through genetic engineering in kernel-using apricot.

## Materials and Methods

### Identification of Aquaporin Family Members in *P. armeniaca*

The predicted peptide sequences of AQP members were acquired from the genome database (unpublished) of kernel-using apricot (*P*. *armeniaca* L.), also known as Longwangmao (Chinese Pinyin name), to construct a local protein database. BLASTP searches were performed using AQP protein sequences of *A. thaliana* and poplar (Table 1) as queries in The *Arabidopsis* Information Resource (TAIR)^1^ and the Phytozome database (release version 12.0^2^) as previously described (Gupta and Sankararamakrishnan, 2009; Zhang et al., 2013), with an E-value of 1e^–10^ and a minimum amino acid identity of 50%. The hidden Markov model (HMM) data of the MIP domain (PF00230) were downloaded from the Sanger database^3^. The PF00230 sequence information was then used to query the *P. armeniaca* protein database via HMMER 3.0 software^4^ with default parameters. Thereafter, the predicted AQP protein sequences were submitted to the National Center for Biotechnology Information (NCBI) Conserved Domain Database (CDD)^5^ (Marchler-Bauer et al., 2002, 2015) and SMART (Simple Modular Architecture Research Tool^6^) (Letunic et al., 2012) to confirm the presence and completeness of the MIP domain, with an E-value threshold of 1e^–2^.

**TABLE 1:** The *PaAQP* gene family identified in *P. armeniaca.*

Sub-family	Gene name	Gene Length (bp)	Transcript Length (bp)	Protein length (aa)	Mw (kDa)	PI	TMHs	GRAVY	Plant-mPLoc	WoLF PSORT
*PIP*	*PaPIP1-1*	2667	858	286	30.85	9.49	6	0.291	plas	plas
	*PaPIP1-2*	2721	858	286	30.66	9.25	6	0.338	plas.	plas
	*PaPIP1-3*	1431	870	290	30.90	9.32	6	0.346	plas	plas
	*PaPIP2-1*	3569	861	287	30.54	8.43	6	0.551	plas	plas
	*PaPIP2-2*	1733	843	281	30.16	7.00	6	0.418	cell wall	plas
	*PaPIP2-3*	1521	852	284	30.10	6.89	6	0.490	plas	plas
	*PaPIP2-4*	1229	843	281	29.89	8.93	6	0.427	plas	plas/cyto_plas/golg
*SIP*	*PaSIP1-1*	5185	732	244	25.90	9.52	6	0.769	plas	plas
	*PaSIP1-2*	4906	729	243	25.78	9.30	6	0.812	Plas	vacu
	*PaSIP1-3*	1075	720	240	25.25	10.00	6	0.870	plas	plas
	*PaSIP2-1*	4084	708	236	25.80	9.38	6	0.564	plas	chlo/nucl/cyto/vacu
*XIP*	*PaXIP1-1*	1103	918	304	32.18	5.97	6	0.713	Cell membrane	plas/golg/vacu
	*PaXIP1-2*	1795	975	324	35.08	7.76	6	0.526	plas	plas/golg/E.R.
*NIP*	*PaNIP1-1*	2168	843	281	29.67	9.27	6	0.440	plas/vacu	plas
	*PaNIP2-1*	2305	987	329	35.37	9.15	6	0.252	plas	plas
	*PaNIP3-1*	1750	795	265	27.60	7.65	7	0.565	plas	chlo/chlo_mito/vacu/mito
	*PaNIP3-2*	3572	894	298	31.01	8.62	6	0.363	plas	plas
	*PaNIP4-1*	1425	801	267	28.10	6.89	6	0.725	plas	plas
	*PaNIP4-2*	2027	789	263	27.52	6.89	6	0.600	plas/vacu	plas
	*PaNIP5-1a*	2456	867	289	30.39	9.17	6	0.603	Cell	chlo
	*PaNIP5-1b*	1598	852	284	30.01	9.58	8	0.696	plas	plas
	*PaNIP6-1*	5078	921	307	31.95	8.34	6	0.415	plas	plas
	*PaNIP7-1*	1875	900	300	32.04	5.92	6	0.521	plas	plas
*TIP*	*PaTIP1-1*	1241	756	252	25.92	5.79	6	0.661	vacu	cyto
	*PaTIP1-2*	1205	756	252	26.07	4.78	6	0.888	vacu	vacu
	*PaTIP1-3*	1554	756	252	25.85	5.32	6	0.752	vacu	vacu/plas/cyto/chlo
	*PaTIP2-1*	1958	744	248	25.38	6.26	6	0.946	vacu	cyto
	*PaTIP2-2*	1411	657	219	21.94	6.90	5	0.990	vacu	plas/vacu
	*PaTIP2-3*	2093	768	256	27.63	9.56	5	0.605	plas	cyto/vacu/plas/chlo/nucl
	*PaTIP3-1*	1258	765	255	25.93	6.25	6	0.788	plas/vacu	chlo/E.R./mito
	*PaTIP3-2*	1156	780	260	27.60	6.43	6	0.610	vacu	cyto
	*PaTIP4-1*	1264	747	249	26.30	5.35	6	0.808	vacu	vacu
	*PaTIP5-1*	1262	765	255	25.92	5.82	6	0.834	plas	chlo/E.R./mito

*Mw, molecular weight; pI, the isoelectric point; GRAVY, grand average of hydropathy. Mw, pI, and GRAVY were predicted by the ExPASy database (Swiss Institute of Bioinformatics, Lausanne, Switzerland) and PROT PARAM tool (http://web.expasy.org/protparam/). Transmembrane helical domains (TMHs) were assessed by TMHMM Sever v.2.0 (http://www.cbs.dtu.dk/services/TMHMM/). Possible cell localization of the proteins was predicted using PlantmPLoc (http://www.csbio.sjtu.edu.cn/bioinf/plant-multi/) and WolF PSORT tool (http://www.genscript.com/wolf-psort.html) (Chlo, chloroplast; Cyto, cytosol; E.R., endoplasmic reticulum; E.R., extracellular; Golg, Golgi apparatus; Mito, mitochondria; Nucl, nuclear; Plas, plasma membrane; Vacu, vacuolar membrane).*

### Sequence Alignments and Phylogenetic and Synthetic Analyses

The PaAQP sequences were aligned with those of AtAQPs from *A. thaliana*^7^ (Johanson et al., 2001) and those of PpeAQPs from *P. persica* (Deshmukh et al., 2015) via the ClustalW program^8^ (Thompson et al., 1994). A phylogenetic tree was constructed by Molecular Evolutionary Genetics Analysis (MEGA) software 7.0 (Kumar et al., 2016; Qu et al., 2018) with the maximum likelihood method and 1,000 bootstrap resamplings. PaAQPs were named and confirmed by sequence homology and phylogenetic analyses. The syntenic relationships between the *P. armeniaca*, *A. thaliana*, and *P. persica* genomes were constructed by MCScan (Tang et al., 2008a,b, 2009) following the protocol for the Plant Genome Duplication Database (Lee et al., 2012). To identify the syntenic regions among *P. armeniaca*, initially, potential homologous gene pairs were detected using the BLASTP algorithm (E < 1e^–5^, top five matches) between every two genomes. Second, the BLASTP results and the gene location information were input into MCScanX software (Wang et al., 2012b). *P. armeniaca* whole genome/segmental and tandem duplications were subsequently identified. All syntenic relationship and gene location data were presented using TBtools software (Chen et al., 2018).

### Structural Features of Putative PaAQPs

The molecular weight (Mw), isoelectric point (pI), and grand average of hydropathy (GRAVY) values were predicted via the ExPASy database (Swiss Institute of Bioinformatics, Lausanne, Switzerland) and ProtParam tool^9^ (Gasteiger et al., 2003). Transmembrane helical domains (TMHs) were assessed via TMHMM Sever 2.0^10^ (Kong et al., 2017). The subcellular localization of the proteins was predicted using WoLF PSORT^11^ (Horton et al., 2007) and PlantmPLoc^12^ (Chou and Shen, 2010). Functional prediction was performed on the basis of two NPA motifs, the ar/R filter (H2, H5, LE1 and LE2), and Froger’s positions (P1-P5), from the multiple sequence alignment results from ClustalW.

### Transient Expression of *PaAQPs* in *A. thaliana* Protoplasts

The *GFP* DNA fragment was amplified by PCR and subcloned into the pBI221 vector (Clontech, Mountain View, CA, United States). The coding regions of *PaPIP1-1*, *PaPIP2-3*, and *PaSIP1-3* cDNA were amplified and fused in frame to the 5′ end of the *GFP* DNA sequence. The six *PaAQP*-*GFP* constructs and the *GFP*-only control construct were introduced into *A. thaliana* protoplasts via a polyethylene glycol-mediated method described previously with some modifications (Sheen, 2001). For plasma membrane localization, the membrane-specific dye FM4-64 was used to stain the transfected protoplasts (Nelson et al., 2007). The fluorescence of FM4-64 and *GFP* in the cells was analyzed with a 551-nm helium-neon laser and a 488-nm argon laser, respectively, via confocal scanning microscopy (TCS SP8, Leica, Germany; *GFP* excitation at 488 nm and emission at 505–525 nm; FM4-64 excitation at 551 nm and emission at 498–588 nm).

### Exon-Intron Structure, 3D Structure, and Conserved Motif Distribution

The exon-intron organization was assessed via the Gene Structure Display Server^13^ based on annotated coding sequences and genome sequences. Conserved domains and motifs were analyzed with MEME^14^. The three-dimensional (3D) structures of the PaAQPs were generated from protein sequences submitted to the Phyre2 server^15^. The transmembrane helices and topology of the PaAQPs were analyzed via the MEMSAT-SVM prediction method on the Phyre2 server.

### *In silico* Analysis of Promoter Sequences and Protein–Protein Interaction Analysis

The promoter sequences (2.0 kb upstream region from the transcription start site) were extracted from the genomic DNA sequences of the *PaAQP* genes. Putative cis-acting regulatory elements of the promoter sequences were identified with the program PlantCARE online^16^. The prediction of interacting networks of proteins was generated from the STRING database^17^.

### cDNA-Library Preparation and Illumina Sequencing for Transcriptome Analysis

Total RNA was extracted from the ED, SP, and GS of the flower buds of two Youyi plants using a Plant RNA Extraction Kit (Autolab, China) following the manufacturer’s protocol. The concentration and quality of each RNA sample were determined using a NanoDrop 2000^TM^ microvolume spectrophotometer (Thermo Scientific, Waltham, MA, United States) and gel electrophoresis. Poly(A) mRNA was isolated from 10 mg of total RNA using magnetic oligo (dT) beads and then divided into short fragments by fragmentation buffer (Ambion, Austin, TX, United States). First-strand cDNA was synthesized with random hexamer primers, and then second-strand cDNA was synthesized using DNA polymerase I (New England Biolabs), RNase H (Invitrogen), buffer, and dNTPs. Short DNA fragments were purified with a QIAquick PCR Purification Kit (Qiagen, Inc., Valencia, CA, United States), subjected to end repair and poly(A) addition and ligated to sequencing adaptors. Suitable fragments (350–450 bp) were purified by agarose gel electrophoresis and gathered by PCR amplification. Six cDNA libraries (ED, SP, and GS of the flower buds of two Youyi plants) were sequenced by using the Illumina HiSeq^TM^ 2000 platform.

To facilitate assembly, the following criteria were used to filter low-quality reads:

1. Filter reads with adapter contamination.
2. Filter reads with unknown nucleotides >5%.
3. Filter reads in which more than 20% of bases showed a Q-value less than 20.

High-quality clean reads was mapped to the reference genome to calculate the expression level in Fragments Per Kilobase of transcript per Million fragments mapped (FPKM).

### Yeast Two-Hybrid Assays

Yeast two-hybrid (Y2H) assays were performed using the Matchmaker Gold Yeast Two Hybrid System (Clontech). The coding regions of the *PaAQP* genes (Supplementary Table 1) and predicted interacting proteins (Supplementary Table 2) were cloned into pGBKT7 and pGADT7 vectors to create different baits and prey, respectively. The different pairs of bait and prey constructs were then cotransformed into yeast strain Gold Y2H, and yeast cells were grown on plates lacking leucine and tryptophan (SD/–Leu/–Trp) for 3 days. Transformed colonies were plated onto quadruple dropout medium (SD/–Leu/–Trp/–His/–Ade) containing 100 μM aureobasidin A and 3 mg mL^–1^ X-α-Gal at 30°C to test for interactions between PaAQPs and predicted interacting proteins.

### Gene Expression Analysis and Quantitative Real-Time-PCR

To study the gene expression profiles of the *PaAQPs* in three periods of dormancy and germination in *P. armeniaca* ‘Youyi,’ RNA sequencing (RNA-seq) data were collected from the transcriptome sequencing data from flower buds collected during the ED stage, SP, and GS of the of two Youyi plants grown under the same conditions.

The bud and stem samples at each stage were collected according to Song et al. (2017). Flower buds were checked and did not burst under forced conditions on the sampling day of PD or ED period, and could burst under forced conditions on the sampling day of SP or GS period. When flower buds are in the SP period, flower buds are not covered by scales, and petals are not visible. While flower buds are in the GS period, they are not covered by scales, and petals are visible. The RNA-seq data of *P. armeniaca* ‘Youyi’ in this study were deposited in the NCBI Sequence Read Archive (SRA) database under number SRS1042411. On the basis of fragments per kilobase of transcript per million mapped reads (FPKM) values, heatmaps and hierarchical clusters were analyzed with HemI 1.0^18^. Quantitative real-time (qRT)-PCR detection was chosen to analyze the expression of *PaAQP* genes in flower buds and stems of three *P. armeniaca* plants exhibiting similar growth during the PD stage, ED stage, SP, and GS. Total RNA was extracted from flower buds and stems using the RNeasy Plant Mini Kit (Qiagen, Valencia, CA, United States), and reverse transcription reactions were performed using Superscript II reverse transcriptase (Invitrogen, Grand Island, NY, United States) according to the manufacturer’s protocols. The primers used for qRT-PCR were designed using Primer Premier 5.0 software (Premier Biosoft Int., Palo Alto, CA, United States), and the specific primer sequences are listed in Supplementary Table 3. qRT-PCR was performed with an ABI Prism 7500 sequence detector (Applied Biosystems, Foster City, CA, United States) and the SYBR ^®^ Premix Ex TaqTM Kit (TaKaRa, Tokyo, Japan) to measure transcript levels. *PaElf* was used as a reference gene in qRT-PCR detection. qRT-PCR analyses were performed in triplicate for each biological sample, and quantitative results were analyzed according to the 2^–ΔΔCT^ method (Livak and Schmittgen, 2001).

### Coexpression Network Analysis

The coexpression data for *PaAQPs* were obtained from RNA-Seq data (SRA database number SRS1042411). For *P. armeniaca* genome-wide coexpression network construction, transcriptome data from the flower buds of two *P. armeniaca* plants were used. Genes with Pearson correlation coefficients >0.876 were selected, and 1,500 genes were screened preliminarily. Among the 1,500 genes coexpressed with *PaAQPs*, 14 genes related to cold resistance and 16 genes associated with cold stress were selected to build the coexpression network of *PaAQPs*. A network diagram was generated according to Cytoscape software (The Cytoscape 1.1 Core was downloaded from^19^).

### Yeast Transformants and Low-Temperature Treatment

The coding DNA sequences (CDSs) of *PaPIP1-1*, *PaPIP1-3*, *PaPIP2-3*, or *PaTIP1-1* were inserted into the yeast expression vector pGAPZA. The recombinant vector pGAPZA:*PaAQP* was introduced into yeast strains. After identifying the positive colonies by PCR, the low-temperature treatment was conducted in solid and liquid Simmons Citrate-Ura (SC-U) media as described previously (Liu et al., 2015). Briefly, colonies of positive clones harboring the *PaAQP* gene or empty-vector (pGAPZA vector) were shaken and cultured at 200 rpm and 30°C for 24 h to induce PaAQP protein expression. A 1-mL aliquot of yeast culture liquid (containing the pGAPZA empty or pGAPZA:*PaAQP* recombinant vectors) was added to the strains under the low-temperature treatment (at an OD600 value of 1.0), which was then cultured at −20°C for 24 h. Afterward, the yeast liquid was diluted 1:10, 100, 1,000, and 10,000, followed by the sequential addition of 4 μL of the original yeast liquid and the diluted liquid to solid SC-U media (consisting of 2% galactose). After 48 h of culture at 30°C, the growth rate of the transformed yeast cells was observed and recorded. In addition, 1 mL of the aliquot yeast (containing the pGAPZA empty vector or pGAPZA:*PaAQP* recombinant vector) liquid at an OD600 value of 1.0 was removed and added to 10 mL of liquid SC-U medium (containing 2% galactose). After 24 h of −20°C treatment and 200 rpm at 30°C, the OD600 value of the transformed yeast cells was measured. A control group that was not stressed was also included.

### Plant Transformation and Low-Temperature Resistance Analysis

The *PaPIP1-3* and *PaTIP1-1* CDSs were cloned into a pBI121 plant expression vector. The pBI121:*PaPIP1-3* and pBI121:*PaTIP1-1* recombinant plasmids were introduced into *Agrobacterium tumefaciens* (LBA4404). Transformation into *Arabidopsis* was carried out via the floral-dip method (Clough and Bent, 2010). To test the effect of cold stress on seedling growth, the seeds were allowed to germinate under normal growth conditions and then transferred to growth chambers at 22, 16, and 16°C 16 h/4°C 8 h. After 10 days of treatment, the physiological indexes of transgenic *Arabidopsis* and wild type plants were determined. Superoxide dismutase (SOD) activity (Wang et al., 2012a) and malondialdehyde (MDA) and proline levels were measured as previously described (Bates et al., 1973). Each sample comprised at least three individual plants, and all physiological analysis experiments were conducted in triplicate.

### Statistical Analyses

The data concerning plant height, gene expression, OD600 values, etc., were assessed by one-way analysis of variance (ANOVA) followed by Tukey’s HSD *post hoc* analysis. All the tests were conducted in triplicate. In the figures, the lowercase letters indicate statistical significance based on one-way ANOVA (*P* < 0.05). Statistical analyses were performed with SPSS 16.0 software (SPSS, Inc., Chicago, IL, United States).

## Results

### Identification, Classification, and Properties of *PaAQP* Genes in *P. armeniaca*

**FIGURE 1:**
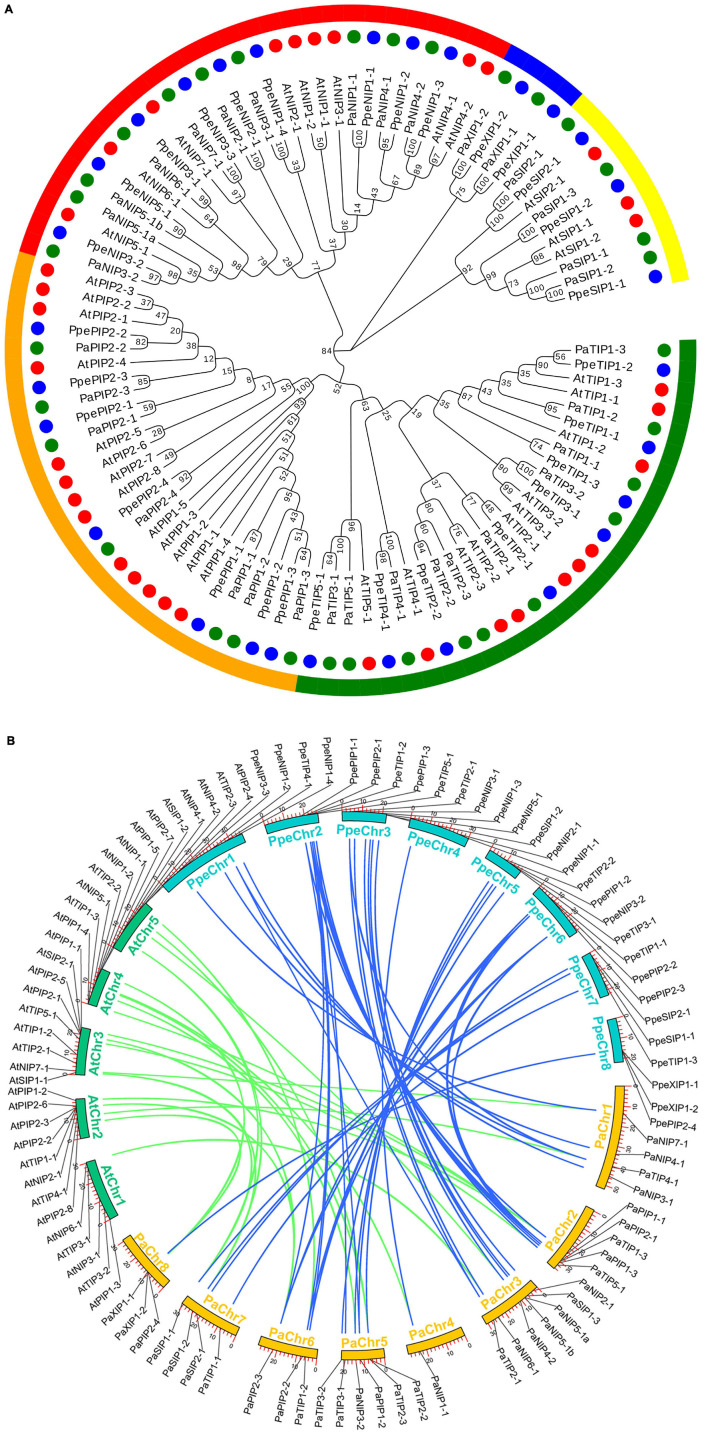
Phylogenetic relationships and microsynteny analyses of *AQP* genes among the *P. armeniaca*, *A. thaliana*, and *P*. *persica* genomes. **(A)** Deduced amino acid sequences were aligned using default parameters in ClustalW, and the phylogenetic tree was constructed via the maximum likely hood method with 1,000 bootstrap replicates with MEGA 7.0 software. Red, green, and blue circles represent the *A. thaliana*, *P. armeniaca*, and *P*. *persica AQP* gene family members. Yellow, blue, red, orange, and green arches represent the *SIP*, *XIP*, *NIP*, *PIP*, and *TIP AQP* gene family subgroups of *A. thaliana*, *P. armeniaca*, and *P*. *persica*. The scale bar indicates the distance calculated by way of multiple alignment. **(B)** The chromosome numbers of all three species are specified by different colors: blue, green, and yellow represent the *P. armeniaca*, *A. thaliana*, and *P*. *persica* chromosomes, respectively. The chromosome number is indicated on the inside with the chromosome sequence lengths in megabases. Gene pairs with syntenic relationships are linked by blue and green lines representing the microsyntenic regions between the *P. armeniaca* and *A. thaliana* chromosomes and the *P. armeniaca* and *P*. *persica* chromosomes, respectively.

Based on an extensive analysis of *AQP*-homologous sequences, a total of 33 putative full-length cDNA sequences of *AQPs* were identified from *P. armeniaca*. To investigate the phylogenetic relationship of AQP proteins among *P. armeniaca*, *A. thaliana*, and *P. persica*, a phylogenetic tree was constructed based on 33 protein sequences of *AQP* genes obtained from *P. armeniaca*, 35 sequences from *A. thaliana*, and 29 sequences from *P. persica* using the maximum likelihood method. The *PaAQPs* were classified into five subfamilies: *NIPs* (10 members), *SIPs* (4 members), *TIPs* (10 members), *XIPs* (2 members), and *PIPs* (7 members). The *NIP* subfamily included seven subgroups (one *NIP1*, one *NIP2*, two *NIP3s*, two *NIP4s*, two *NIP5s*, one *NIP6*, and one *NIP7*). The *TIP* subfamily was divided into five subgroups (three *TIP1s*, three *TIP2s*, two *TIP3s*, one *TIP4*, and one *TIP5*). The *SIP* subfamily was divided into two subgroups (three *SIP1s* and one *SIP2s*). The *PIP* subfamily was further clustered into three *PIP1* and four *PIP2* subgroups, and two members (*XIP1s*) belonged to the *XIP* subfamily (Figure 1A and Supplementary Table 1). All of these genes were grouped with *A. thaliana* and *P. persica AQPs*, confirming that they belonged to the *AQP* family.

Synteny analysis was carried out using MCScanX software, and syntenic relationships and whole genome sequences to visualize the locations of orthologous genes were presented using TBtools software. Microsynteny analysis was performed across the *P. armeniaca*, *A. thaliana*, and *P. persica* genomes. Between *P. armeniaca* and *A. thaliana*, 14 collinear blocks were identified, while 22 orthologous gene pairs were found (Figure 1B). Between *P. armeniaca* and *P. persica*, 30 collinear blocks were identified, with 41 orthologous gene pairs (Figure 1B). These results suggest that *P. armeniaca* and *P. persica* are more closely related than *P. armeniaca* and *A. thaliana*.

Bioinformatics analysis, CDS and amino acid sequence analysis, and ExPaSy database analysis showed that the Mw of the PaAQPs ranged from 21.94 to 35.37 kDa and that their pIs ranged from 5.32 to 10.00 (Table 1). The SIPs and TIPs were smaller (<28 kDa) than the NIPs, XIPs, and PIPs. The TIPs were acidic (except for PaTIP2-3), while the majority of the other subfamilies were alkaline. Most of the AQPs were predicted to have TMHs, while PaTIP2-2 and PaTIP2-3 had only five, PaNIP3-1 had seven, and PaNIP5-1b had eight TMHs (Table 1). The GRAVY scores of the PaAQP proteins ranged from 0.291 to 0.990, and among the subfamilies, the PIPs presented the lowest average GRAVY value (0.409) (Table 1).

### Chromosomal Distribution, Gene Structure, and Microsynteny Analysis

The *PaAQPs* showed less variation in transcript length (ranging from 657 to 987 bp) than in gene length (ranging from 1075 to 12,213 bp). The number of amino acids in the identified *PaAQPs* ranged from 219 (PaTIP2-2) to 329 (PaNIP2-1) (Table 1). The 33 identified *PaAQPs* were unevenly mapped on the eight chromosomes of *P. armeniaca.* Chromosome 3 contained the largest number (7; 21.21%) of *PaAQP* genes, followed by chromosomes 5 and 2, which contained six members (18.18%) and five members (15.15%), respectively. Both chromosomes 1 and 7 contained four members (12.12%). Chromosome 8 contained three members (9.09%), and chromosome 4 contained only one member (3.03%) (Supplementary Figure 1).

Gene duplication has long been considered one of the main forces in the evolution and expansion of a gene family (Crow and Wagner, 2006). Therefore, we investigated the different types of gene duplications in the *PaAQP* gene family. We found that 6 *AQP* genes clustered in 3 tandem repeat event regions in the *P. armeniaca* genome (Supplementary Figure 1). There were three pairs of tandem duplicated genes (*PaNIP5-1a* and *PaNIP5-1b*, *PaTIP2-2* and *PaTIP2-3*, *PaXIP1-1*, and *PaXIP1-2*) within the 1 Mb chromosomal region of chromosomes 3, 5, and 8. In addition, 6 pairs of WGD/segmental duplication genes (*PaTIP4-1* and *PaTIP2-1*, *PaTIP1-2* and *PaTIP1-3*, *PaSIP1-1*, and *PaSIP1-2*, triangle WGD/segmental duplication among *PaPIP2-1*, *PaPIP2-2*, and *PaPIP2-3*) were found within the *PaAQP* family (Supplementary Figure 1). Notably, all the identified segmental duplication gene pairs were distributed on different chromosomes in the *P. armeniaca* genome. However, the distribution of segmental replication genes on the chromosomes was uneven; there were three genes on chromosome 6, two on chromosomes 2 and 7, and only one on chromosome 1. Moreover, we also found that *PaPIP2-1* was the common duplicated gene between *PaPIP2-2* and *PaPIP2-3*. This particular gene replication event involved three genes: *PaPIP2-1*, which was located on chromosome 2, and *PaPIP2-2* and *PaPIP2-3* on chromosome 6. Additionally, the analysis revealed one particular WGD/segmental duplication event between chromosomes 2 and 6 involving four genes. *PaPIP2-1* combined with *PaTIP1-3* was reverse duplicated to *PaPIP2-2* combined with *PaTIP1-2*.

Exon-intron organization plays key roles in phylogenetic relationships and plant evolution (Wang et al., 2014; Jue et al., 2015). As shown in Supplementary Figure 2, the distribution of introns in the *PaAQP* genes ranged from one to five introns per gene. All *PaPIPs* presented four exons: *PaXIP1-1* had two exons, and *PaXIP1-2* had three exons. The majority of *PaSIPs* exhibited three exons; an exception involved *PaSIP1-3*, which had only one exon and was therefore intronless. Most *PaTIPs* had three exons, while *PaTIP1-1* had two and *PaTIP2-3* had five. A relatively diverse exon-intron pattern was detected for the *PaNIP* subfamily. The majority of *PaNIPs* had five exons, with the exceptions of *PaNIP3-2*, which had six exons, *PaNIP5-1a*, which had four exons, and *PaNIP3-1*, which had three exons. Exon-intron organization analysis revealed a conserved pattern of gene structure within the subfamilies of *PaAQPs.* The conserved exon-intron structure supports the phylogenetic relationships of *P. armeniaca* (Supplementary Figure 2).

### Characterization of Asn-Pro-Ala Motifs, Transmembrane Domains, and Conserved Motifs of PaAQPs

**FIGURE 2:**
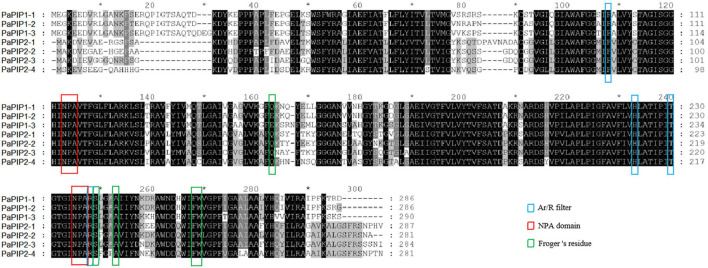
Multiple sequence alignment of the deduced amino acid sequences of PaPIPs. Amino acids at NPA domains, ar/R selectivity filters, and Froger’s residues were identified in seven *PIP* family members (3 *PIP1s* and 4 *PIP2s*) in *P. armeniaca*.

To better understand the possible physiological mechanisms of the PaAQPs, all 33 sequences were aligned, and the conserved residues (NPA motifs, ar/R selectivity filter, and Froger’s position) were analyzed (Figure 2, Table 2, and Supplementary Figures 3–6). The *P. armeniaca* PaAQPs displayed differences in NPA motifs and residues at the ar/R selectivity filters and Froger’s positions compared with those of other plant species. Most of the AQPs showed dual NPA motifs, with the exception of PaSIP2-1, which was found to harbor a single NPA motif. The majority of members from the PIP and TIP subfamilies contained a typical NPA motif, as observed for the *Beta vulgaris* counterpart (Kong et al., 2017), with the exception of PaTIP2-3, which showed an alanine (A)-to-serine (S) substitution in the first NPA motif and asparagine (N)-to-A and A-to-lysine (K) substitutions in the secondary NPA motif. The LPK (PaTIP2-3) motifs detected in *P. armeniaca* have not been reported in any other plant species. These changes may alter the substrate specificity of the AQPs in *P. armeniaca*. In the NIP subfamily, some members of the NIPs, such as PaNIP5-1a, PaNIP3-2, PaNIP5-1b, and PaNIP6-1, showed replacement of alanine (A) at the third residue of the first NPA motif by serine (S) and replacement of other alanine (A) residues by isoleucine (I) or valine (V). In the XIP subfamily, the first NPA motif showed an N-to-S substitution in PaXIP1-1 and a valine (V)-to-A substitution in PaXIP1-2, while the second NPA motif was conserved. All PIP subfamily members showed conserved ar/R filter residues, with phenylalanine (F) at H2, histidine (H) at H5, threonine (T) at LE1, and arginine (R) at LE2, which is typical of water-transporting AQPs. They also showed Q/E-S-A-F-W residues at Froger’s positions, as observed in species such as flax (*Linum usitatissimum*) (Shivaraj et al., 2017) and chickpea (*Cicer arietinum* L.) (Deokar and Bunyamin, 2016). In the TIP subfamily, the H2 position of the ar/R filter contained histidine (H), and the H5 position contained isoleucine (I), except in PaTIP1-1, where this position was occupied by valine (V), and PaTIP2-3, which did not exhibit a residue at this position. The LE1 and LE2 positions were found to be specific to each subgroup of PaTIP. The PaTIP1 subgroup was characterized by alanine (LE1) and valine (LE2) levels. The PaTIP2 subgroup was characterized by glycine (LE1) and arginine (LE2), except for PaTIP2-2, which contained glycine (LE2), and PaTIP2-3, which contained alanine (LE1) and leucine (LE2). The PaTIP3 and PaTIP4 subgroups were characterized by alanine (LE1) and arginine (LE2), with the exception of PaTIP3-1, which contained glycine (LE1) and cystine (LE2). PaTIP5 was also characterized by glycine (LE1) and cystine (LE2) and showed a conserved relationship with chickpea (Deokar and Bunyamin, 2016) and flax (Shivaraj et al., 2017). Among the NIPs, the ar/R selectivity filter and Froger’s positions presented multiple types. PaNIP1-1, PaNIP3-1, PaNIP4-1, and PaNIP4-2 showed W-R-A-F/V/I in the ar/R selectivity filter and F/L-S-A-Y-I/T/F residues at Froger’s positions, and PaNIP5-1a and PaNIP5-1b showed S-R/Q-G-V in the ar/R selectivity filter and F-T-A-Y-L residues at Froger’s positions. PaNIP2-1, PaNIP3-2, PaNIP6-1, and PaNIP7-1 showed G/A/T-R-G/A-F/I in the ar/R selectivity filter. The SIP family members showed A-V-P-N residues in the ar/R selectivity filter and M-A-A-Y-W at Froger’s positions, with the exception of PaSIP1-3, which contained isoleucine (H2), and PaSIP2-1, which showed T/H/G/S in the ar/R selectivity filter and F-V-A-Y-W at Froger’s positions. The ar/R selectivity filter of PaXIP1-1 and PaXIP1-2 was I/V/V/R, and Froger’s positions consisted of V-C-A-F-W, showing a conserved relationship with flax (Shivaraj et al., 2017).

**TABLE 2:** Amino acid composition of the NPA motifs, ar/R selectivity filter, and Froger’s residues of *PaAQP*.

Gene name	NPA (LB)	NPA (LE)	ar/R selectivity filters				Froger’s Residue				
			H2	H5	LE1	LE2	P1	P2	P3	P4	P5
*PaPIP1-1*	NPA	NPA	F	H	T	R	Q	S	A	F	W
*PaPIP1-2*	NPA	NPA	F	H	T	R	E	S	A	F	W
*PaPIP1-3*	NPA	NPA	F	H	T	R	E	S	A	F	W
*PaPIP2-1*	NPA	NPA	F	H	T	R	Q	S	A	F	W
*PaPIP2-2*	NPA	NPA	F	H	T	R	Q	S	A	F	W
*PaPIP2-3*	NPA	NPA	F	H	T	R	Q	S	A	F	W
*PaPIP2-4*	NPA	NPA	F	H	T	R	Q	S	A	F	W
*PaSIP1-1*	NPT	NPA	A	V	P	N	M	A	A	Y	W
*PaSIP1-2*	NPT	NPA	A	V	P	N	M	A	A	Y	W
*PaSIP1-3*	NPS	NPA	I	V	P	N	M	A	A	Y	W
*PaSIP2-1*	–	NPA	T	H	G	S	F	V	A	Y	W
*PaNIP1-1*	NPA	NPA	W	V	A	F	F	S	A	Y	I
*PaNIP2-1*	NPA	NPA	G	S	G	F	L	T	A	Y	V
*PaNIP3-1*	NPA	NPA	W	V	A	V	F	S	A	Y	T
*PaNIP3-2*	NPS	NPV	A	I	G	I	F	T	A	Y	L
*PaNIP4-1*	NPA	NPA	W	V	A	V	L	S	A	Y	F
*PaNIP4-2*	NPA	NPA	W	V	A	I	L	S	A	Y	I
*PaNIP5-1a*	NPS	NPI	S	I	G	V	F	T	A	Y	L
*PaNIP5-1b*	NPS	NPI	S	I	G	V	F	T	A	Y	L
*PaNIP6-1*	NPS	NPV	T	I	A	I	F	T	A	Y	L
*PaNIP7-1*	NPA	NPA	A	V	G	I	Y	S	A	Y	I
*PaTIP1-1*	NPA	NPA	H	V	A	V	T	S	A	Y	W
*PaTIP1-2*	NPA	NPA	H	I	A	V	T	S	A	Y	W
*PaTIP1-3*	NPA	NPA	H	I	A	V	T	S	A	Y	W
*PaTIP2-1*	NPA	NPA	H	I	G	R	T	S	A	Y	W
*PaTIP2-2*	NPA	NPA	H	I	G	G	T	R	L	−	−
*PaTIP2-3*	NPS	LPK	H	−	A	L	T	P	A	V	T
*PaTIP3-1*	NPA	NPA	N	V	G	C	V	A	A	Y	W
*PaTIP3-2*	NPA	NPA	H	I	A	R	T	A	A	Y	W
*PaTIP4-1*	NPA	NPA	H	I	A	R	T	S	A	Y	W
*PaTIP5-1*	NPA	NPA	N	V	G	C	V	A	A	Y	W
*PaXIP1-1*	SPV	NPA	I	V	V	R	V	C	A	F	W
*PaXIP1-2*	NPV	NPA	I	V	V	R	V	C	A	F	W

To further analyze the conservation, diversity, and relationship in each subfamily, motif scan analysis was conducted via the MEME program. Seventeen conserved motifs were identified in the PaAQP family, and most of the members in the same subfamily shared a similar number of motifs (Supplementary Figure 7). For example, all the members of PaPIPs had thirteen motifs, except for PaPIP1-2. All the members of PaTIP1s had eleven motifs, all the members of PaSIP1s had nine motifs, and all the members of PaNIP4s had eleven motifs. The results also showed that PaAQPs had similar functional motifs (motif 2) consisting of highly conserved regions (channel proteins of the major intrinsic protein (MIP) superfamily) with motif 4 from *B. vulgaris* BvAQP proteins. These results implied that PaAQPs may have a similar function to AQPs from other plants.

### Promoter Cis-Element Analysis

Promoter sequence analysis indicated that the *PaAQP* genes contained several development-related, stress-, and hormone-responsive cis-acting regulatory elements, including low-temperature-responsive elements (LTEs), light-responsive elements (GT1s, MREs, TCT-motifs and G-boxes), auxin-responsive elements (TGAs and AuxRR-cores), abscisic acid (ABA)-response elements (ABREs), dehydration-responsive elements (MBS), endosperm expression elements (AACA motifs and GCN4 motifs), and wound-responsive elements (WUN motifs) (Supplementary Figure 8 and Supplementary Table 4). Among the stress-related cis-acting elements, all *PaAQPs* (33 genes) exhibited AREs (essential for anaerobic induction). There were 17 low temperature-responsive (LTR) and 16 MBS (drought inducible) elements within 14 and 12 *PaAQP* promoters, respectively. This finding indicated that AQP proteins may play an important functional role in the stress response and tolerance in *P. armeniaca*. Among the hormone-related cis-acting elements, 76 ABREs (ABA responsive), 38 CGTCA motifs (methyl jasmonate (MeJA) responsive), and 37 TGACG motifs (MeJA responsive) were identified in the promoters of 23, 22, and 21 *PaAQPs*, respectively. Furthermore, large numbers of elements related to light responsiveness, zein metabolism regulation (O_2_ sites), meristem expression (CAT-boxes) and some elements related to flavonoid biosynthesis (MBSIs) and endosperm expression were detected, implying that *PaAQPs* might participate in developmental processes in *P. armeniaca*.

### Molecular Modeling of PaAQP

3D proteins of plant AQPs can be effectively used for understanding the substrate specificity and solute permeation rate of *AQP* genes, which will help to improve the understanding of the cold resistance of *PaAQPs* (Groot and Grubmüller, 2005). The structural properties of all 33 PaAQPs were displayed in homology-based tertiary (3D) protein models, which were predicted via the Phyre2 server display, and the results are shown in Supplementary Table 5. All 3D protein models were constructed with 100% confidence, and the residue coverage varied from 68 to 98%. The PaAQP 3D protein model contained a conserved hourglass-like structure consisting of an a-helical bundle forming five to eight TM helices (H1 to H8) and two short helices (HE and HB). Loops HE and HB were located close to each in the center of the membrane. These predicted 3D models showed the boron (B) and silicon (Si) substrate selectivity of the PaAQPs and provided an important basis for the protein functional analysis of the *AQP* genes of *P. armeniaca* (Deshmukh et al., 2013; Tombuloglu et al., 2016).

### Protein–Protein Interaction Network of PaAQPs

**FIGURE 3:**
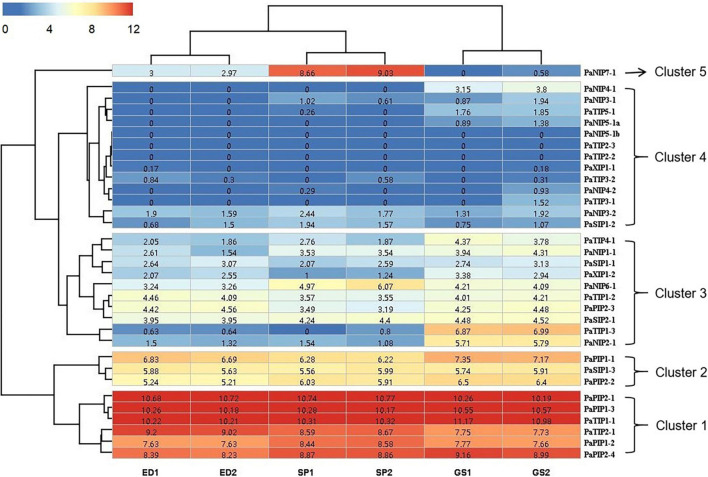
Expression profiles of *PaAQPs* at different developmental stages based on RNA-seq data. ED, SP, and GS indicate that the tested materials were collected during ecological dormancy, the sprouting period, and germination stage of *P. armeniaca*, respectively. The number in the middle of each box represents the FPKM.

**FIGURE 4:**
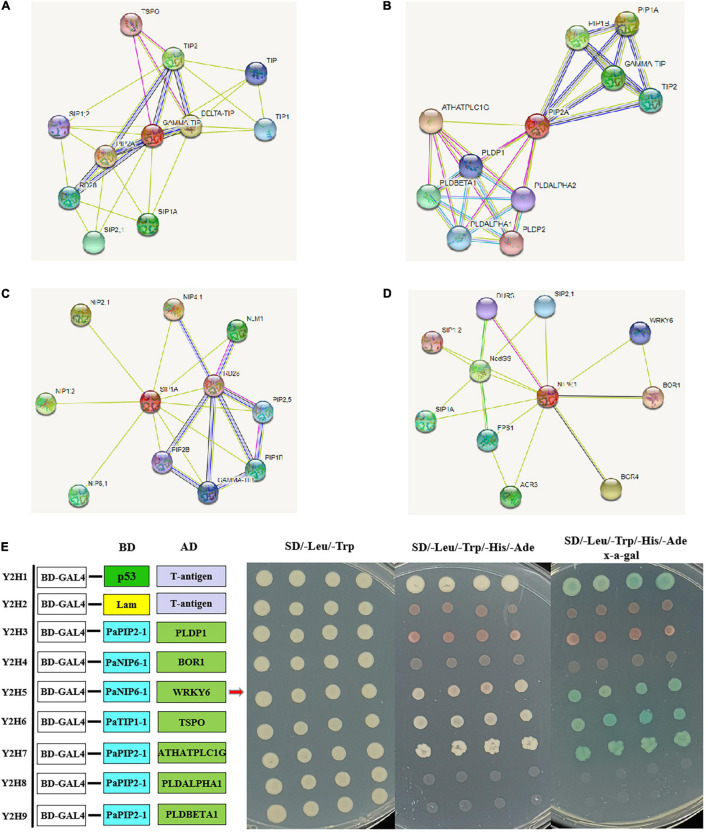
Prediction of the interaction network of PaAQP proteins based on the interactions of their orthologs in *A. thaliana*. **(A)** The interaction network of TIP. **(B)** The interaction network of PIP. **(C)** The interaction network of SIP. **(D)** The interaction network of NIP. **(E)** Y2H assays showing the protein interaction between PaNIP6-1 and WRKY6, PaTIP1-1 and TSPO, and PaPIP2-1 and ATHATPLC1G. The networks were generated from the STRING database. The red circles represent the queried protein, while the other circles are the interacting proteins. The annotation of the predicted interacting protein was derived from UniProtKB (https://www.uniprot.org/). The line thickness indicates the strength of the data support.

To further reveal the function of PaAQPs during the interaction with other proteins, PaTIP1-1, PaPIP1-3, PaSIP1-3, and PaNIP6-1, which showed high expression in each subfamily (Figure 3), were used to construct a protein-protein interaction network. As shown in Figures 4A–D and Supplementary Table 6, none of these tested PaAQPs shared the same interacting proteins. In addition, various proteins specifically interacted with TIP separately, such as zinc finger proteins, calcium-dependent protein kinases, and NAC domain-containing proteins. In particular, the interacting zinc finger proteins and NAC transcription factors can enhance cold tolerance in transgenic plants (Jung et al., 2013). Interestingly, TIP2 could interact with TIP and form homodimers. The proteins that interact with PIP include AGD2-like defense response protein, methylenetetrahydrofolate reductase family proteins, and Delta 1-pyrroline 1–5-carboxylate reductase. Specifically, SIP and NIP members could interact with each other. For example, NIP6-1 specifically interacted with SIP1-3, SIP2-1, and SIP1A, while four NIP members (NIP1-2, NIP2-1, NIP4-1, and NIP6-1) and three PIP members (PIP1B, PIP1B, and PIP2-5) could interact with SIP1A. Notably, NIP6-1 specifically interacted with WRKY6, which plays important roles in cold resistance, stress tolerance, growth and development (Wang et al., 2014). Y2H assays showed that yeast cells cotransformed with constructs PaNIP6-1-pGBKT7 and WRKY6-pGADT7, PaTIP1-1-pGBKT7 and TSPO-pGADT7, and PaPIP2-1-pGBKT7 and ATHATPLC1G-pGADT7 could support growth in the selection medium and b-galactosidase staining (Figure 4E). Thus, Y2H assays revealed the protein interaction between PaNIP6-1 and WRKY6, PaTIP1-1 and TSPO, and PaPIP2-1 and ATHATPLC1G. In particular, the *A. thaliana* gene encoding a phosphatidylinositol-specific phospholipase C AtPLC1 (ATHATPLC1G) was induced by low-temperature, dehydration, and salt stress, which might contribute to the adaptation of the plant to cold stresses (Hirayama et al., 1995).

### Expression Profiling of *PaAQPs* in the Dormancy and Sprouting Stages of *P. armeniaca*

To gain more information about the potential role of PaAQP proteins in *P. armeniaca*, we analyzed the expression of *PaAQPs* in the ED, SP, and GS by transcriptome sequencing. The RNA-seq data can be found in the NCBI SRA database under number SRS1042411. The expression levels of 33 *PaAQP* genes were clustered in a heatmap (Figure 3). We observed the expression of 30 *PaAQPs* (90.90%) in at least one of the developmental stages, and all of these *PaAQPs* showed differential expression in three stages of dormancy and sprouting periods. Among these genes, 23 *PaAQP* genes (75%) were expressed in all three periods of dormancy and germination, among which *PaPIP2-1*, *PaPIP1-3*, and *PaTIP1-1* exhibited the greatest expression levels in different periods. In addition, *PaTIP1-2* showed greater expression in the ED period than in the SP and GS periods, which may suggest that the *P. armeniaca* plant was in the ED period. Similarly, *PaNIP7-1* showed greater expression in the SP period than in the ED and GS periods and may be useful as a marker gene of entry into the SP. *PaTIP1-3* and *PaNIP2-1* exhibited greater expression levels in the GS than in the ED and SP, so these two genes might be useful for suggesting that the *P. armeniaca* plant is entering the GS. Interestingly, *PaNIP5-1b*, *PaTIP2-3*, and *PaTIP2-2* were not expressed in any of the three periods of dormancy and germination. In addition to these three genes, *PaNIP3-1*, *PaNIP4-1*, *PaNIP4-2*, *PaNIP5-1a*, *PaTIP3-1*, and *PaTIP5-1* were not expressed in the ED period of flower buds; and *PaNIP5-1a*, *PaNIP4-1*, and *PaTIP3-1* were not expressed in the SP of flower buds.

The heatmap demonstrated the clustering of four main clades. The *PaAQP* genes from clade 1 corresponded to 2 *TIP* members (*PaTIP1-1* and *PaTIP2-1*) and 4 *PIP* members (*PaPIP2-1*, *PaPIP1-3*, *PaPIP1-2*, and *PaPIP2-4*) displaying the greatest expression in three periods of dormancy and germination. The genes from clade 2 included 2 *PIP* members (*PaPIP1-1* and *PaPIP2-2*) and 1 *SIP* member (*PaSIP1-3*) that exhibited relatively high expression in these three periods, while those from clade 3 included 3 *NIP* members (*PaNIP1-1*, *PaNIP2-1*, and *PaNIP6-1*), 3 *TIP* members (*PaTIP1-2*, *PaTIP1-3*, and *PaTIP4-1*), 2 *SIP* members (*PaSIP1-1* and *PaSIP2-1*), 1 *PIP* member (*PaPIP2-3*), and 1 *XIP* member (*PaXIP1-2*) showing an intermediate level of expression, and clade 4 included 7 *NIP* members (*PaNIP3-1*, *PaNIP3-2*, *PaNIP4-1*, *PaNIP4-2*, *PaNIP5-1a*, *PaNIP5-1b*, and *PaNIP7-1*), 5 *TIP* members (*PaTIP2-2*, *PaTIP2-3*, *PaTIP3-1*, *PaTIP3-2*, and *PaTIP5-1*), 1 *SIP* member (*PaSIP1-2*), and 1 *XIP* member (*PaXIP1-1*) displaying the lowest expression in three developmental periods of flower buds.

### Expression Patterns of *PaAQP* Genes in Different Tissues During the Dormancy and Sprouting Stages of *P. armeniaca*

**FIGURE 5:**
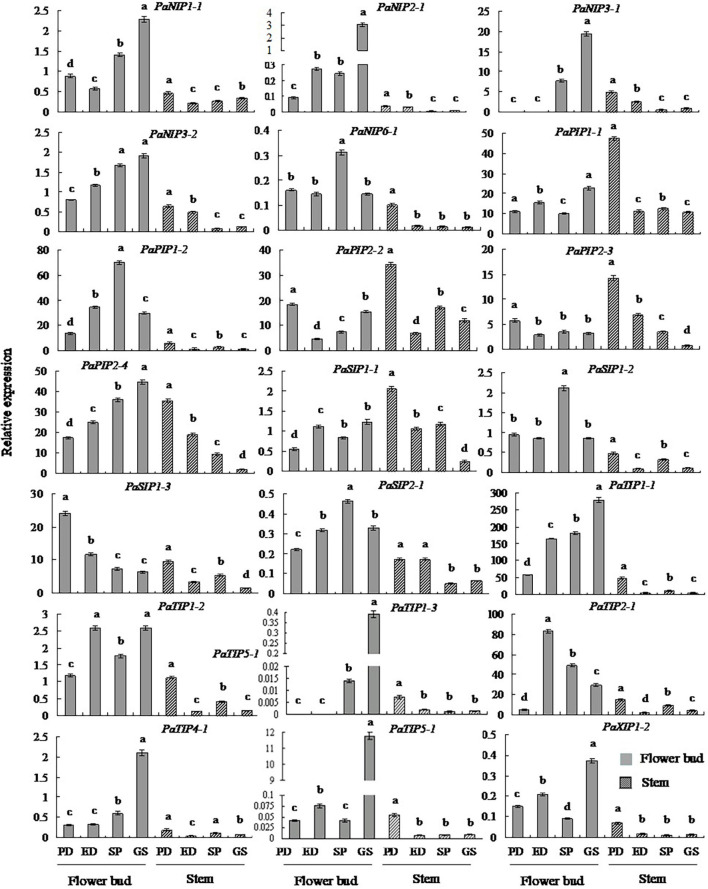
Changes in the transcript levels of 20 selected genes in different tissues during the dormancy and sprouting stages of *P. armeniaca*. The stem and flower buds were collected during the PD stage, ED stage, SP stage, and GS stage of *P. armeniaca* plants. All expression levels of *PaAQP* genes were normalized to the expression levels of *PaElf* (elongation factor-1α). The 2^–ΔΔCT^ method was used to analyze the relative gene expression. The data are means of three replicates ± SDs. The lowercase letters indicate statistical significance based on one-way ANOVA with Tukey’s HSD *post hoc* analysis.

To gain insight into potential functions, qRT-PCR was employed to determine the expression patterns of *PaAQP* genes in two tissues: flower buds and stems (Figure 5 and Supplementary Figure 9). All 20 key *PaAQP* genes were expressed in two tested tissues with different expression patterns. Nine genes (*PaPIP2-3*, *PaSIP1-1*, *PaSIP1-3*, *PaSIP2-1*, *PaTIP1-2*, *PaNIP1-1*, *PaNIP3-2*, *PaXIP1-2*, and *PaTIP4-1*) with stable expression patterns in all stages of dormancy and germination, five genes (*PaPIP1-1*, *PaPIP2-2*, *PaNIP2-1*, *PaNIP6-1*, and *PaTIP1-3*) with differential expression patterns, three highly expressed genes (*PaTIP1-1*, *PaTIP2-1*, and *PaPIP2-4*), and three genes with low expression (*PaNIP3-1*, *PaTIP5-1*, and *PaSIP1-2*) were selected for qRT-PCR analysis. The qPCR results for the 20 selected *PaAQPs* were generally consistent with the transcript-level changes determined by RNA-seq analysis in the dormancy and sprouting stages of flower buds, suggesting that the transcriptomic profiling data were likely reliable. However, moderate discrepancies in the transcript levels were detected in two *PaAQPs* compared with the RNA-seq data: *PaPIP2-3* and *PaSIP1-3* (Figure 5). The expression of *PaPIP2-3* and *PaSIP1-3* in the stems showed almost the same trends as in the flower buds in the four examined stages of *P. armeniaca. PaPIP1-1*, *PaPIP2-2*, *PaPIP2-3*, and *PaSIP1-1* showed the greatest expression during PD in the stem, and *PaPIP2-4*, *PaSIP2-1*, *PaXIP1-2*, *PaNIP1-1*, *PaNIP2-1*, *PaNIP3-1*, *PaTIP1-1*, *PaTIP1-3*, *PaTIP4-1*, and *PaTIP5-1* showed the greatest expression in the GS in the flower buds (Figure 5). These results suggested that the differential expression of *PaAQP* members during the dormancy and sprouting stages in different tissues could predict the different biological functions of these genes.

### Coexpression Network of PaAQPs in *P. armeniaca*

**FIGURE 6:**
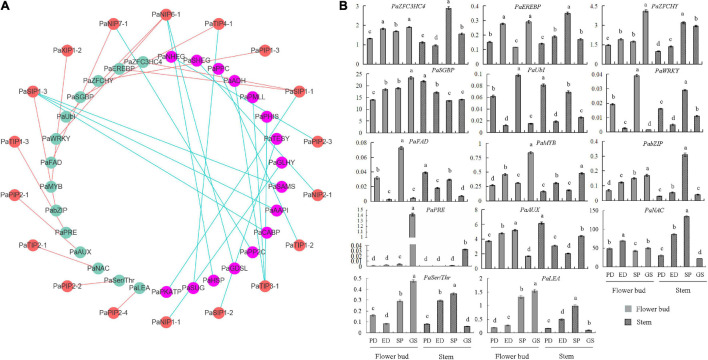
*PaAQP* coexpression network. **(A)** Among the 1,500 genes coexpressed with *PaAQPs*, 14 (green nodes) are involved in cold resistance based on previous research. In addition, the anticold ability of 16 genes (pink nodes) is not very clear. The red node is the *PaAQP* genes. Cyan lines represent *PaAQPs* coexpressed with 14 cold resistance genes, and red lines represent *PaAQPs* coexpressed with 16 cold stress-associated genes. **(B)** Transcript levels of 14 genes involved in cold resistance in stem and flower buds during the PD stage, ED stage, SP stage, and GS stage of *P. armeniaca*. All expression levels of the *PaAQP* genes were normalized to those of *PaElf*. Each bar represents the mean ± SD of three technical replicates. The different letters represent significant differences at *P* < 0.05 (one-way ANOVA).

To elucidate the molecular mechanism of *PaAQPs* in the dormancy and sprouting stages of *P. armeniaca*, a *PaAQP* coexpression network was constructed (Figure 6A and Supplementary Table 7). A total of 1,500 genes were identified as coexpressed with *PaAQPs*. Among these, 14 genes (0.93%) were associated with cold resistance. For example, C3HC4-type RING zinc finger proteins are involved in cold stress (Jung et al., 2013). MYB transgenic rice enhances tolerance to chilling stress and mediates ectopic expression of stress genes (Ma et al., 2009). bZIP transcription factors regulate freezing tolerance in *Arabidopsis* (Yao et al., 2020). Additionally, overexpression of the NAC transcription factor enhances the cold tolerance of *Medicago truncatula* (Qu et al., 2016). Serine/threonine protein kinases are involved in the cold stress response in the cyanobacterium *Synechocystis* (Zorina et al., 2014). In our research, zinc finger proteins, *MYBs*, *bZIPs*, *NACs*, and serine/threonine protein kinases, among others, were highly coexpressed with *PaAQPs* in different tissues during the dormancy and sprouting stages of *P. armeniaca* and may be involved in cold resistance in *P. armeniaca*.

To validate the network constructed by the core *PaAQPs*, changes in the expression of the 14 genes related to cold resistance in the *PaAQP* coexpression network were detected in the PD, ED, SP, and GS of flower buds and stems of *P. armeniaca* plants (Figure 6B). Among the 14 pairs of coexpressed genes, the expression trends of *PaSIP1-1* and *PaZFC3HC4*, *PaSIP1-1* and *PaEREBP* were consistent in the four periods (PD, ED, SP, and GS) of flower buds of *P. armeniaca*, whereas those of *PaTIP4-1* and *PaSGBP* were rarely consistent. Similarly, the expression was similar between *PaNIP6-1* and *PaUbI* and between *PaNIP6-1* and *PaWRKY*. Additionally, three pairs exhibited no differences in expression patterns: *PaXIP1-2* and *PaMYB*, *PaTIP1-3* and *PaPRE*, *PaPIP2-4* and *PaLEA*.

### Subcellular Localization of PaPIP1-1, PaPIP2-3, PaSIP1-3, PaXIP1-2, PaNIP6-1, and PaTIP1-1 Proteins

WoLF PSORT and Plant-mPLoc prediction were used to predict the subcellular localization of the PaAQPs. Almost all members of the PaPIP subfamilies were localized to the plasma membrane according to WoLF PSORT and Plant-mPLoc prediction (Table 1). However, the subcellular localizations predicted by Plant-mPLoc indicated that PaPIP2-2 was localized to the cell wall. The PaTIPs were predicted to localize to the vacuoles, and PaNIPs (except for PaNIP5-1a),PaSIPs, and PaXIPs were predicted to localize to the plasma membrane according to Plant-mPLoc prediction. However, the results predicted by WoLF PSORT were diverse and included localization to the nucleus, Golgi, cytosol, mitochondrion, chloroplast, and endoplasmic reticulum (Table 1). Genes with high expression in different periods of low temperature may play important roles in cold resistance during the dormancy and sprouting stages of *P. armeniaca*. To identify and gain insight into the subcellular localization of these highly expressed *PaAQP* genes in plant cells, chimeric *EGFP*-*PaPIP1-1*, *EGFP*-*PaPIP2-3*, *EGFP*-*PaSIP1-3*, *EGFP*-*PaXIP1-2*, *EGFP*-*PaNIP6-1*, and *EGFP*-*PaTIP1-1* cDNAs (representing genes with the greatest expression in each subgroup in flower buds in the dormancy and germination of *P. armeniaca*) were constructed and inserted downstream of the *CaMV35S* promoter. Transient expression analyses were performed using *A. thaliana* leaf mesophyll cells.

**FIGURE 7:**
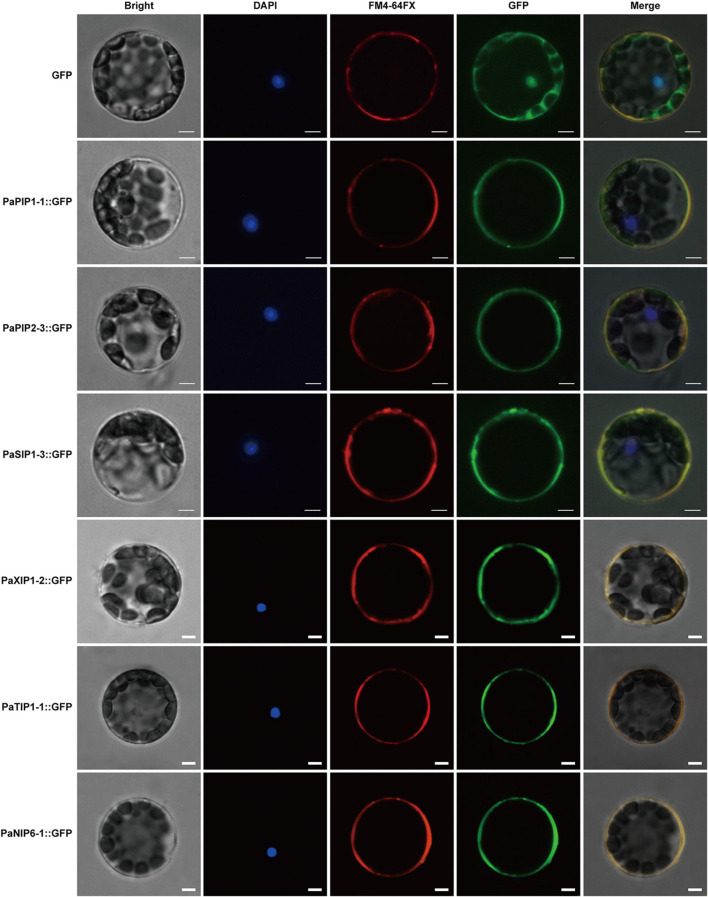
Localization of PaPIP1-1, PaPIP2-3, PaSIP1-3, PaXIP1-2, PaNIP6-1, and PaTIP1-1 in the cell plasma membrane. Bright, Bright-field images. DAPI, nuclei counterstained with 4′,6-diamidino-2-phenylindole (DAPI), a nuclear marker; FM4-64FX, FM4-64FX dye images, a plasma membrane-specific vital dye. Merge, overlap of *GFP* (green) and FM4-64FX (red) fluorescence. Row 1 shows the protoplasts expressing *GFP* alone, which was used as a control. Row 2 shows protoplasts expressing the *PaPIP1-1*:*GFP* fusion protein with FM4-64 fluorescence. Row 3 shows the protoplasts expressing the *PaPIP2-3*:*GFP* fusion protein with FM4-64 dye. Row 4 shows protoplasts expressing the *PaSIP1-3*:*GFP* fusion protein with FM4-64 fluorescence. Row 5 shows the protoplasts expressing the *PaXIP1-2*:*GFP* fusion protein with FM4-64 dye. Row 6 shows the protoplasts expressing the *PaNIP6-1*:*GFP* fusion protein with FM4-64 dye. Row 7 shows protoplasts expressing the *PaTIP1-1*:*GFP* fusion protein with FM4-64 dye. FM4-64FX shows a plasma membrane-specific dye. Bars = 5 μm.

Analyses of fusion protein localization by confocal microscopy showed that the green fluorescence of these proteins was confined to the plasma membrane (Figure 7). This membrane localization of the PaPIP1-1, PaPIP2-3, and PaSIP1-3 proteins was consistent with the location of PIP and SIP gene expression in other plants (Zou et al., 2015; Deokar and Bunyamin, 2016).

### The *PaPIP1-3* and *PaTIP1-1* Genes Conferred Low-Temperature Resistance to Yeast

**FIGURE 8:**
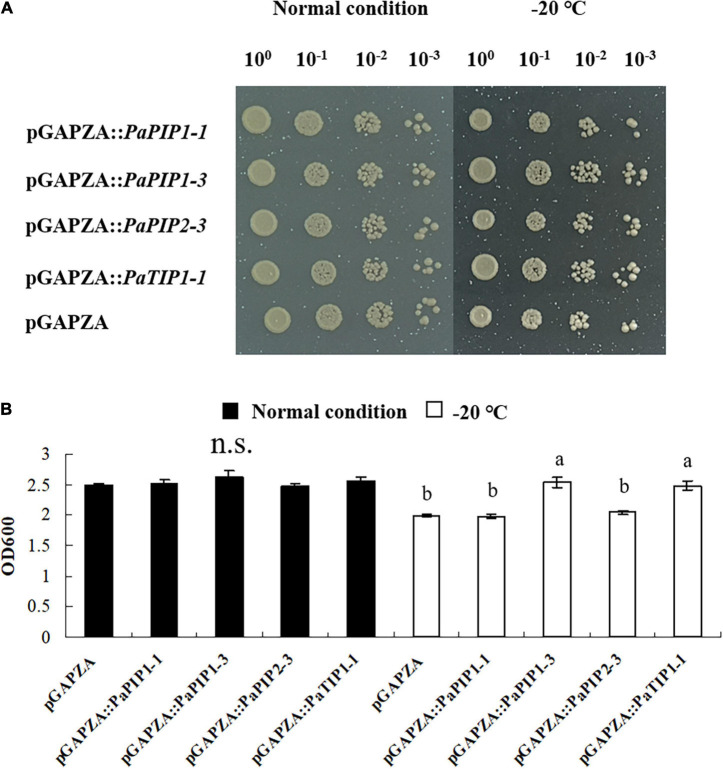
Overexpression of *PaPIP1-3* and *PaTIP1-1* increases cold tolerance and accumulation in yeast. **(A)** Growth of the GS115 yeast strain transformed with the empty vector pGAPZA or with pGAPZA harboring *PaPIP1-1*, *PaPIP1-3*, *PaPIP2-3*, or *PaTIP1-1*. **(B)** OD600 value of yeast transformants in response to low-temperature stress. The data are the means ± SDs of three replications. n.s. means not significant. The different lowercase letters indicate significant differences at *P* < 0.05 by ANOVA.

To analyze the function of *PaAQP* genes involved in low-temperature tolerance, four genes that showed high expression in flower buds and stems during the dormancy and sprouting stages of *P*. *armeniaca* were expressed in yeast strain GS115 to check their low-temperature stress resistance. The yeast strains transformed with the pGAPZA empty vector or various pGAPZA:*PaAQP* recombinant expression vectors were incubated in solid and liquid SC-U medium (2% galactose) for low-temperature stress treatment. The growth of yeast cells transformed with pGAPZA:*PaPIP1-3* and pGAPZA:*PaTIP1-1* on solid SC-U media consisting of 2 M sorbitol after −20°C low-temperature treatment grew much more than did the cells transformed with empty vector. Moreover, the colonies of yeast cells harboring the *PaPIP1-3* and *PaTIP1-1* genes were larger than those harboring empty vectors strains (Figure 8A). In 2 M liquid SC-U sorbitol media, the mean OD value of the empty-vector strains was 2.33, whereas the positive strains harboring *PaPIP1-3* and *PaTIP1-1* had OD values of 2.73 and 2.55, respectively. After the −20°C low-temperature treatment, the mean OD values were 1.99 vs. 2.54 and 2.48 (Figure 8B). These results suggested that the *PaPIP1-3* and *PaTIP1-1* genes improved the low-temperature stress resistance of yeast cells.

### Ectopic Expression of *PaPIP1-3* and *PaTIP1-1* in *A. thaliana* Conferred Cold Tolerance

**FIGURE 9:**
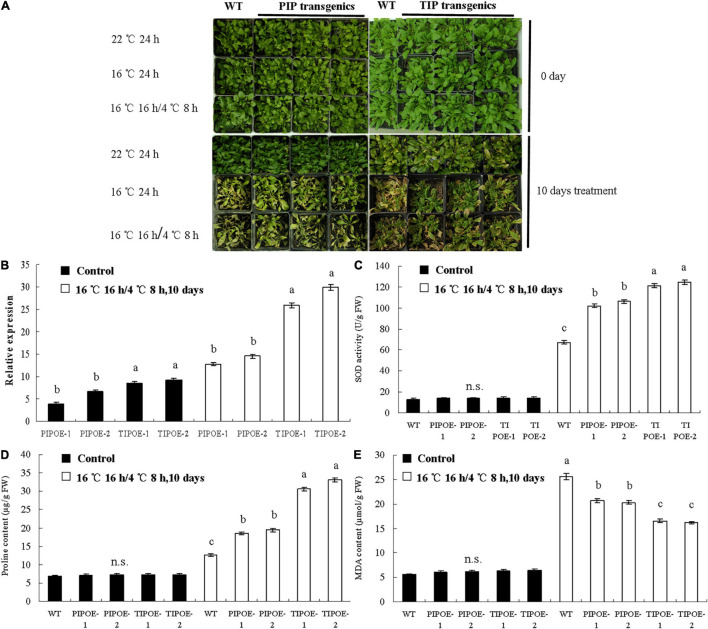
Cold tolerance analysis of wild-type and *PaPIP1-3-* or *PaTIP1-1*-OE plants. **(A)** Image of the wild-type and transgenic plants in growth chambers at 22, 16, and 16°C for 16 h/4°C 8 h. Two independent *PaPIP1-3* transgenic lines (PIPOE-1and PIPOE-2) and two independent *PaTIP1-1* transgenic lines (TIPOE-1and TIPOE-2), were identified by qRT-PCR. **(B)**. After 10 days of treatment, the SOD activity **(C)**, proline content **(D)**, and MDA content **(E)** of *PaPIP1-3* or *PaTIP1-1* transgenic *Arabidopsis* and wild-type *Arabidopsis* were determined. PIPOE- 1-, PIPOE- 2-, and *PaPIP1-3*-OE transgenic lines; TIPOE- 1-, TIPOE- 2-, and *PaTIP1-1*-OE transgenic lines. Actin was used as a control gene. The error bars represent the SEs of three replicates. n.s. means not significant. The different letters represent significant differences at *P* < 0.05 (one-way ANOVA).

To study the function of the *PaAQP* genes in cold stress, two genes (*PaPIP1-3* and *PaTIP1-1*) exhibiting cold stress resistance in yeast were introduced individually into *A. thaliana* plants. Normally growing wild-type *Arabidopsis* and *PaPIP1-3* or *PaTIP1-1* gene-carrying *Arabidopsis* were transferred to growth chambers at 22, 16, or 16°C 16 h/4°C 8 h. After 10 days of treatment, the wild-type plants showed significantly high sensitivity to cold stress with yellow leaves. The growth of *PaPIP1-3-* or *PaTIP1-1*-overexpressing (OE) *Arabidopsis* lines (PIPOE-1, PIPOE-2, TIPOE-1, and TIPOE-2) was better than that of the wild-type plants, demonstrating good cold resistance (Figure 9A). After low temperature treatment, two independent *PaPIP1-3* transgenic lines (PIPOE-1 and PIPOE-2) and two independent *PaTIP1-1* transgenic lines (TIPOE-1 and TIPOE-2), which with higher relative expression level than untreated transgenic lines, were identified by qRT-PCR (Figure 9B). We further examined the activities of SOD, the major ROS antioxidant enzyme in plants. The PIPOE-1 and PIPOE-2 lines and the TIPOE-1 and TIPOE-2 lines had significantly greater SOD activity than the wild-type line (Figure 9C). The superior performance of the *PaPIP1-3-* and *PaTIP1-1*-transformed plants was further validated by analyzing the proline and MDA contents subsequent to cold stress. The proline level was significantly greater in the transgenic leaves than in the wild-type leaves under the stress conditions, indicating superior biochemical capabilities of the transgenic plants (Figure 9D). Similarly, the MDA content was greatest in wild-type leaves of cold-stressed plants, showing that the greatest membrane damage had occurred in untransformed plants (Figure 9E). This research indicated that the target genes *PaPIP1-3* or *PaTIP1-1* were directly related to cold resistance, and the increase in *PaPIP1-3* or *PaTIP1-1* expression in *PaPIP1-3-* or *PaTIP1-1*-transformed plants could improve the physiological condition and cold resistance of transgenic plants.

## Discussion

Aquaporins are varied and are widely distributed and functionally diverse in plant genomes. These proteins play important roles in the transport of water and substrates in plants, in regulating plant development and growth, and in responses to drought, cold, or salt stresses (Prado and Maurel, 2013; Kong et al., 2017). A majority of the genome-wide analyses and functional studies of AQP genes conducted to date have focused on model plants such as *Z*. *mays*, *A*. *thaliana*, *O*. *sativa*, *N*. *tabacum*, and *Populus* (Chaumont et al., 2001; Gupta and Sankararamakrishnan, 2009; Nguyen et al., 2013). Very little is known about the AQPs of economic forest species such as *P. armeniaca*. To determine the evolutionary relationships and functional mechanisms of PaAQPs, a phylogenetic tree was built using AQPs from *A*. *thaliana* (in which a full set of *AtAQP* genes is well known) and *Prunus persica* (Deshmukh et al., 2015), which are closely related species of *P. armeniaca*. The number of *AQPs* identified in this study was the same as that reported in *Brachypodium distachyon* (Deshmukh et al., 2015), being slightly greater than the 30 *AQP* genes reported in *Elaeis guineensis* (Deshmukh et al., 2015) and *Vitis vinifera* (Deshmukh et al., 2015) and lower than the 33 genes found in rice (Sakurai et al., 2005), 35 genes found in *A. thaliana* (Zhang et al., 2013), and 37 genes found in *Ricinus communis* (Deshmukh et al., 2015). The number of *P. armeniaca AQPs* in the five subfamilies was also similar to their distribution in *Cajanus cajan* (Deshmukh et al., 2015). However, the numbers of *PIPs* and *TIPs* varied in the two genomes compared with those of the other subfamilies. Twelve and thirteen members of *PIPs* and *TIPs* have been reported in *C. cajan* (Deshmukh et al., 2015), while seven *SIPs* and ten *TIPs* have been detected in *P. armeniaca*. *PaTIPs* are divided into five subgroups, *TIP1*, *TIP2*, *TIP3*, *TIP4*, and *TIP5*, and this classification is consistent with those previously reported in species such as barley, sugar beet, rice, and maize (Tombuloglu et al., 2016; Kong et al., 2017).

The GRAVY score is an important property of AQPs that facilitates high water permeability (Murata et al., 2000), and the GRAVY scores of the PaAQP proteins can reflect their hydrophobic nature. The lowest average GRAVY value was found in the PIP subfamily, indicating good hydrophilicity of PaPIPs toward water molecules. Topological analysis predicted that almost all PaAQPs had six transmembrane helical domains (Table 2), which is consistent with the structure identified in other plants, such as flax (*L. usitatissimum*) (Shivaraj et al., 2017), *Hevea brasiliensis* (Zhi et al., 2016), and *B. vulgaris* (Kong et al., 2017). However, PaTIP2-2 and PaTIP2-3 showed a partial loss of TM6, and the effect of the missing protein domains on protein functions must be confirmed. The absence of the TM6 domain may affect the protein folding, transport activity, and subcellular localization of PaTIPs (Hu et al., 2012). Almost all PaTIPs were located in vacuoles, while PaTIP2-3 was localized in the plasma membrane based on Plant-mPLoc prediction.

An unrooted phylogenetic tree was constructed to analyze the evolutionary relationships based on the protein sequences of the PaAQPs, in which one PaSIP (PaSIP1-3), three PaNIPs (PaNIP3-2, PaNIP5-1a, and PaNIP5-1b), and one PaXIP (PaXIP1-2) aroused our attention and caused confusion in the classification and cluster analysis. SIP1-3 and NIP3-2 were not found in *A. thaliana*; PaSIP1-3 shared the greatest similarity of 61.41% with PtSIP1-3 (Supplementary Table 8), while for PaNIP3-2 and PaXIP1-2, the closest homolog of PaNIP3-2 was PpeNIP3-2 (Figure 1A), and PaXIP1-2 clustered closer to PpeXIP1-2 of *P*. *persica*. *PaNIP5-1a* and *PaNIP5-1b* are tandemly duplicated genes located at the same site on chromosome 3.

The number of *PaAQP* genes (33) in the *P. armeniaca* genome is lower than that in *A. thaliana* (35) and greater than that in *P. persica* (29). However, while the genome size of *P. armeniaca* (222 Mb) is slightly smaller than that of *P. persica* (226 Mb) (Verde et al., 2013), it is larger than that of *A. thaliana* (164 Mb). The 33 PaAQP proteins were categorized into five clades. We further observed that most of the *PaAQP* genes were closely related to the *AQP* genes in *P. persica*, consistent with the finding that *P. armeniaca* and *P. persica* diverged from a recent common ancestor. It is well known that gene duplication mechanisms (WGD/segmental duplication, tandem duplication) have a significant role in biological evolution (Panchy et al., 2016). In the present study, we observed that orthologous genes developed through segmental duplication and tandem duplication. Both styles of duplication have played a significant role in the expansion of *P. armeniaca AQP* family genes. The identification of orthologous *PaAQP* genes will further benefit the evolutionary history of this gene family.

Asn-Pro-Ala motifs, the ar/R selectivity filter, and Froger’s positions are considered to participate in transport activity and substrate selection. PaAQP functions can be speculated based on the amino acid residues of the proteins compared with the AQPs of other plants. The PaPIPs showed typical NPA motifs, F-H-T-R residues in the ar/R selectivity filter, and Q/E-S-A-F-W at Froger’s positions, which are highly conserved in the PIPs of other plants, such as flax (*L. usitatissimum*) (Shivaraj et al., 2017), chickpea (*C. arietinum* L.) (Deokar and Bunyamin, 2016) and *H. brasiliensis* (Murata et al., 2000). This composition of PIPs is considered to play a vital role in facilitating CO_2_ diffusion, regulating leaf and root hydraulics, and influencing plant photosynthesis (Gupta and Sankararamakrishnan, 2009). Therefore, the homologous PIPs of *P. armeniaca* may play similar roles in regulating plant growth and development, such as in photosynthetic physiology, plant hydraulics, and water and solute transport. Plant TIPs have been suggested to function as water transporters as efficiently as PIPs (Murata et al., 2000). For example, PaTIP1-1, BvTIP1-2, and BvTIP1-3 contain dual conserved NPA motifs, H-I/V-A-V at the ar/R selectivity filter and T-S-A-Y-W at Froger’s positions, which is the same composition observed for the *Citrus sinensis* AQPs CsTIP1s and CsTIP3s. Additionally, these proteins are predicted to function in the transport of urea and H_2_O_2_ (Hove and Bhave, 2011). Compared with other TIPs, PaTIP1-2, PaTIP1-3, and PaTIP2-1 showed H-I-A-V or H-I-G-R at the ar/R selectivity filter and at Froger’s positions and harbored T-S-A-Y-W residues at the P1-P5 positions; this structure type is considered to transport substances such as NH_4_, urea, and H_2_O_2_ (Hove and Bhave, 2011). These analyses suggest that PaTIPs play an important role in the transport of a wide range of molecules.

WoLF PSORT predicted that all the PaPIP members were localized in the plasma membrane, and plant-mPLoc prediction indicated that the PaNIPs (except for PaNIP5-1a) were also localized in the plasma membranes. These results were consistent with experimental findings for PIPs and NIPs of banana, flax, and other plant species (Maurel et al., 2008; Shivaraj et al., 2017). However, some tobacco NtPIP members show dual localization in the plasma membrane and inner chloroplast membrane (Uehlein and Kaldenhoff, 2008), and different localizations of a maize *PIP* gene (*ZmPIP1-2*) have been found in the endoplasmic reticulum and plasma membrane (Chaumont et al., 2000). NIPs also exhibit different subcellular localizations in the peribacteroid membrane, endoplasmic reticulum, or plasma membrane in other plants (Takano et al., 2006; Deokar and Bunyamin, 2016). According to our predictions, both PaNIP1-1 and PaNIP4-2 were localized to the plasma membrane and vacuoles. The reasons for the different localizations of these genes are not clear. The majority of SIPs have been shown to localize to the vacuoles and plasma membrane; however, grapevine and *A. thaliana* SIPs are predicted to be localized to the endoplasmic reticulum (Ishikawa et al., 2005; Noronha et al., 2014; Kong et al., 2017). In addition to differences between species and protein sequences, many experiments have shown that external environmental conditions such as drought and salinity, but not cold, influence the characteristics of the subcellular localization of PaAQPs (Boursiac et al., 2008; Deokar and Bunyamin, 2016). These results indicate that the subcellular localization of plant AQPs is complex and regulated by environmental conditions.

*Aquaporin* genes are expressed in different plant tissues and organs, and their spatiotemporally specific expression is highly correlated with different developmental stages (Alexandersson et al., 2005; Giorgio et al., 2016). The expression patterns of *AQPs* in various tissues as well as under different stresses may provide an efficient basis for identifying their molecular functions. qRT-PCR results showed that *PaAQPs* were expressed in all tissues tested, but their transcript levels largely differed in various organs (Figure 5), suggesting that the functions of *PaAQPs* might vary. For example, the expression levels during the endodormancy stage (*PaPIP2-3*, *PaPIP2-2*, and *PaSIP1-3* are high) indicated that the effects of growth inhibition were controlled by other internal factors resulting from insufficient low-temperature accumulation. High expression levels during the ecodormancy stage (*PaTIP1-2* and *PaTIP2-1*) could reflect the activation of growth through release from endogenous inhibitors but maintained suppression due to unsuitable conditions (insufficient high temperature accumulation). Expression during SP (*PaSIP1-2*, *PaNIP3-2*, and *PaNIP6-1* are high) and GS (*PaPIP1-1*, *PaPIP2-4*, *PaSIP1-1*, *PaSIP2-1*, *PaXIP1-2*, *PaNIP2-1*, *PaNIP1-1*, *PaNIP3-1*, *PaTIP1-1*, *PaTIP1-3*, and *PaTIP4-1* are high) showed morphological growth resumption, not (endo)dormancy release. Moreover, *PaPIP1-1*, *PaPIP2-2*, *PaPIP2-3*, and *PaSIP1-1* were highly expressed in stems, where they may contribute to the storage of energy and materials needed for growth, development, and germination.

In the *PaAQP* coexpression network, similar expression patterns of gene pairs (such as *PaSIP1-1* and *PaZFC3HC4*, *PaSIP1-1* and *PaEREBP*, *PaNIP6-1* and *PaUbI* and *PaNIP6-1* and *PaWRKY*) during the dormancy and sprouting stages suggested that *PaAQPs* cooperated with various vital cold stress- or flower bud dormancy/germination-related genes to regulate cold tolerance, bud dormancy, and bud break. For example, a novel zinc-finger protein (homologous gene of *PaZFC3HC4*) mediates ABA-regulated seed dormancy in *Arabidopsis* (He and Gan, 2004), and an ubiquitin-conjugating enzyme (homologous gene of *PaUbI*) has been found to be involved in tuber dormancy breaking (Liu et al., 2012). A total of 68 *AP2/ERF* genes were first identified in dormant Chinese cherry flower buds. Some *PpcAP2/ERF* TFs (homologous genes of *PaEREBP*) participate in and influence the dormancy transition through their responses to low temperature (Zhu et al., 2021). Chen et al. (2016) found that the expression levels of six *WRKY* genes increased during endodormancy and decreased during ecodormancy, indicating that these six WRKY genes may play a role in dormancy in peach. However, several coexpression pairs, such as *PaSIP1-3* and *PabZIP* and *PaTIP2-1* and *PaNAC*, exhibited different expression regularities, implying that these gene pairs may have different roles and functions in different periods during the dormancy and sprouting stages of *P. armeniaca.*

*Aquaporins* regulate long-distance water transport between cells and can mediate the osmotic pressure of the cytoplasm to appropriately regulate plant growth and development, responses to environmental stress, and stomatal movement, among others. *PaTIPs* can mediate rapid water transport between cells, remove accumulated H_2_O_2_, and improve water transport activity, which is necessary to end dormancy (Erez et al., 1998; Yooyongwech et al., 2008). In our study, several *PaTIPs*, such as *PaTIP1-1*, *PaTIP1-2*, and *PaTIP4-1*, showed greater transcript levels in all four dormancy and sprouting stages of *P. armeniaca*. *PaTIP1-2* showed greater expression in the ED period than SP and GS, which may represent a response to cold stress to aid in the removal of the accumulated induced H_2_O_2_, thereby promoting the end of dormancy. In particular, overexpression of *PaTIP1-1* increased cold tolerance and accumulation in yeast (Figure 8), which may help to improve water transport activity and promote normal growth under low-temperature stress. Previous research has indicated that *AtPIP2-5* and *AtPIP2-6* are upregulated by cold stress in *A. thaliana* plants (Alexandersson et al., 2005). Transgenic banana plants overexpressing *MusaPIP1-2* show a high tolerance to cold conditions (Shekhawat and Ganapathi, 2013). *A. thaliana* plants overexpressing *AtPIP1-4* or *AtPIP2-5* display greater cold tolerance than wild-type plants (Afzal et al., 2016). In our study, *PaPIP1-2* and *PaPIP2-4* exhibited high expression in the dormancy and sprouting stages in the flower buds of *P*. *armeniaca*. Interestingly, yeast cells expressing *PaPIP1-3* and *PaTIP1-1* exhibited markedly enhanced growth compared with that of yeast cells transformed with the empty vector on SG-U medium with low-temperature treatment (Figure 8), and *PaPIP1-3* or *PaTIP1-1* overexpressed in *Arabidopsis* showed enhanced cold tolerance by improving antioxidative enzyme activities (Figure 9). These results may suggest that PaPIPs present stronger cold resistance and play a vital role in the maintenance of normal growth and development under cold conditions.

## Conclusion

Overall, this work presents the first genome-wide study of the *P. armeniaca AQP* gene family, in which 33 non-redundant *PaAQP* genes were identified in the *P. armeniaca* genome and phylogenetically clustered into five distinct subfamilies. Synteny analysis identified 14 collinear blocks containing *AQP* genes between *P. armeniaca* and *A. thaliana* and 30 collinear blocks between *P. armeniaca* and *P. persica.* The chromosome localization, gene structure, protein characteristics, conserved functional motifs, and homology-based 3D models of the *PaAQP* genes were further examined. Cis-motif analysis of the 2.0-kb sequences upstream of the *AQP* genes indicated the existence of several light-, hormone-, and stress-responsive and developmental stage-specific elements in the *PaAQPs*. The subcellular localization of PaPIP1-1, PaPIP2-3, PaSIP1-3, PaXIP1-2, PaNIP6-1, and PaTIP1-1 was examined, and the green fluorescence of the recombinant proteins was observed in the plasma membrane of isolated protoplasts. *In silico* analysis of protein-protein interactions suggested the association of PaAQP proteins with specific cold tolerance and development pathways. Gene expression profiling of *PaAQP* showed that *PaPIP1-3*, *PaPIP2-1*, and *PaTIP1-1* were highly expressed in flower buds during the dormancy and sprouting stages of *P. armeniaca.* Significantly, *PaPIP1-3* and *PaTIP1-1* conferred low-temperature stress resistance in a yeast expression system. Overexpression of *PaPIP1-3* and *PaTIP1-1* in *Arabidopsis* plants increased antioxidative enzyme activities and improved transgenic plant tolerance to cold stress. This study provides an important resource for the future functional analysis of PaAQPs and a theoretical basis for the breeding of new varieties via the genetic engineering of *P. armeniaca*.

## Data Availability Statement

The datasets presented in this study can be found in online repositories. The names of the repository/repositories and accession number(s) can be found in the article/Supplementary Material.

## Author Contributions

SL and T-NW conceived and designed the research. SL, YX, and SW performed all the experiments. YZ, GZ, XZ, and JL contributed reagents, materials, and analysis tools. JZ, XG, LW, and WM analyzed the data. SL wrote the manuscript. All authors read and approved the final manuscript.

## Conflict of Interest

The authors declare that the research was conducted in the absence of any commercial or financial relationships that could be construed as a potential conflict of interest.

## Publisher’s Note

All claims expressed in this article are solely those of the authors and do not necessarily represent those of their affiliated organizations, or those of the publisher, the editors and the reviewers. Any product that may be evaluated in this article, or claim that may be made by its manufacturer, is not guaranteed or endorsed by the publisher.

## Footnotes

1
http://www.arabidopsis.org

2
https://phytozome.jgi.doe.gov/pz/portal.html

3
http://pfam.xfam.org/family/PF00230

4
http://hmmer.org/

5
http://www.ncbi.nlm.nih.gov/Structure/cdd/wrpsb.cgi

6
http://smart.embl-heidelberg.de/

7
https://www.arabidopsis.org/

8
http://www.ebi.ac.uk/clustalw/

9
http://web.expasy.org/protparam/

10
http://www.cbs.dtu.dk/services/TMHMM/

11
http://www.genscript.com/wolf-psort.html

12
http://www.csbio.sjtu.edu.cn/bioinf/plant-multi/

13
http://gsds.cbi.pku.edu.cn/

14
http://meme-suite.org/tools/meme

15
http://www.sbg.bio.ic.ac.uk/phyre2/html/page.cgi?id=index

16
http://bioinformatics.psb.ugent.be/webtools/plantcare/html/

17
https://string-db.org/?tdsourcetag=s_pctim_aiom

18
http://hemi.biocuckoo.org/down.php

19
http://www.cytoscape.org/

## Supplementary Material

Supplementary Figure 1 Distribution of *PaAQP* genes on the eight *P. armeniaca* chromosomes. Thirty-three *PaAQP* genes are distributed across all eight *P. armeniaca* chromosomes. The right side of the chromosomal bar represents the *PaAQP* genes, and the corresponding positions on the chromosome (megabase pairs; Mb) are given on the left side. The *PaNIP* genes are in black, the PaPIP genes are in red, the *PaSIP* genes are in purple, the *PaXIP* genes are in green, and the *PaTIP* genes are in magenta. Tandemly duplicated genes are underlined. Whole genome/segmental duplications are lined and underlined.

Supplementary Figure 2 Exon-intron structures of the 33 *PaAQP* genes. The yellow boxes denote exons, and the lines denote introns. The exon-intron structure was determined via GSDS 2.0 (http://gsds.cbi.pku.edu.cn/) on the basis of gene and CDSs.

Supplementary Figure 3 Protein sequence alignment of PaNIPs identified in *P. armeniaca* showing the amino acids at NPA domains, ar/R filters, and Froger’s residues.

Supplementary Figure 4 Protein sequence alignment of PaTIPs identified in *P. armeniaca* showing the amino acids at NPA domains, ar/R filters, and Froger’s residues.

Supplementary Figure 5 Protein sequence alignment of PaSIPs identified in *P. armeniaca* showing the amino acids at NPA domains, ar/R filters, and Froger’s residues.

Supplementary Figure 6 Protein sequence alignment of PaXIPs identified in *P. armeniaca* showing the amino acids at NPA domains, ar/R filters, and Froger’s residues.

Supplementary Figure 7 Distribution of conserved motifs in the *PaAQP* proteins. The motifs were identified via the online MEME program. The different colored boxes indicate different motifs at the corresponding positions.

Supplementary Figure 8 Various *cis*-acting elements in the promoter of *PaAQP* genes. The numbers of ARE (AAACCA), ABRE (ACGTG), MBS (CAACTG), MRE (AACCTAA), and other *cis*-acting elements in the promoter region of *P. armeniaca PaAQP* genes from each *AQP* family. The cis-acting elements were determined in the 2-kb promoter region upstream of the translation initiation site using the PLACE database.

Supplementary Figure 9 qRT-PCR analysis of the relative expression of 20 genes during the dormancy and sprouting stages of *P. armeniaca*.

Supplementary Table 1 Complete coding sequences and corresponding amino acid sequences of the *AQP* genes of *P. armeniaca*.

Supplementary Table 2 The coding regions of *WRKY6*, *TSPO*, and *ATHATPLC1G* can interact with *PaAQP* genes.

Supplementary Table 3 Primers used in the qRT-PCR analysis of the *PaAQP* genes, and cold resistance genes coexpressed with *PaAQP* genes.

Supplementary Table 4 Analysis of cis-acting elements in the 2,000-bp sequence upstream of the translation initiation codon of *PaAQP* genes.

Supplementary Table 5 Predicted 3D structure and transmembrane region of the 33 PaAQP proteins.

Supplementary Table 6 Predicted functional partners of TIP, PIP, SIP, and NIP. Function of the top 10 highest scoring interacting proteins in each subfamily.

Supplementary Table 7 Genes related to cold resistance in the *PaAQP* coexpression network.

Supplementary Table 8 Amino acid sequences of *AQP* genes in poplar.
