# Supplementary figures and images for: Genome-Wide Identification and Function of Aquaporin Genes During Dormancy and Sprouting Periods of Kernel-Using Apricot (Prunus armeniaca L.)
Source: Front Plant Sci. 2021 Oct 4;12:690040. doi: 10.3389/fpls.2021.690040 (PMC8520955; doi:10.3389/fpls.2021.690040)

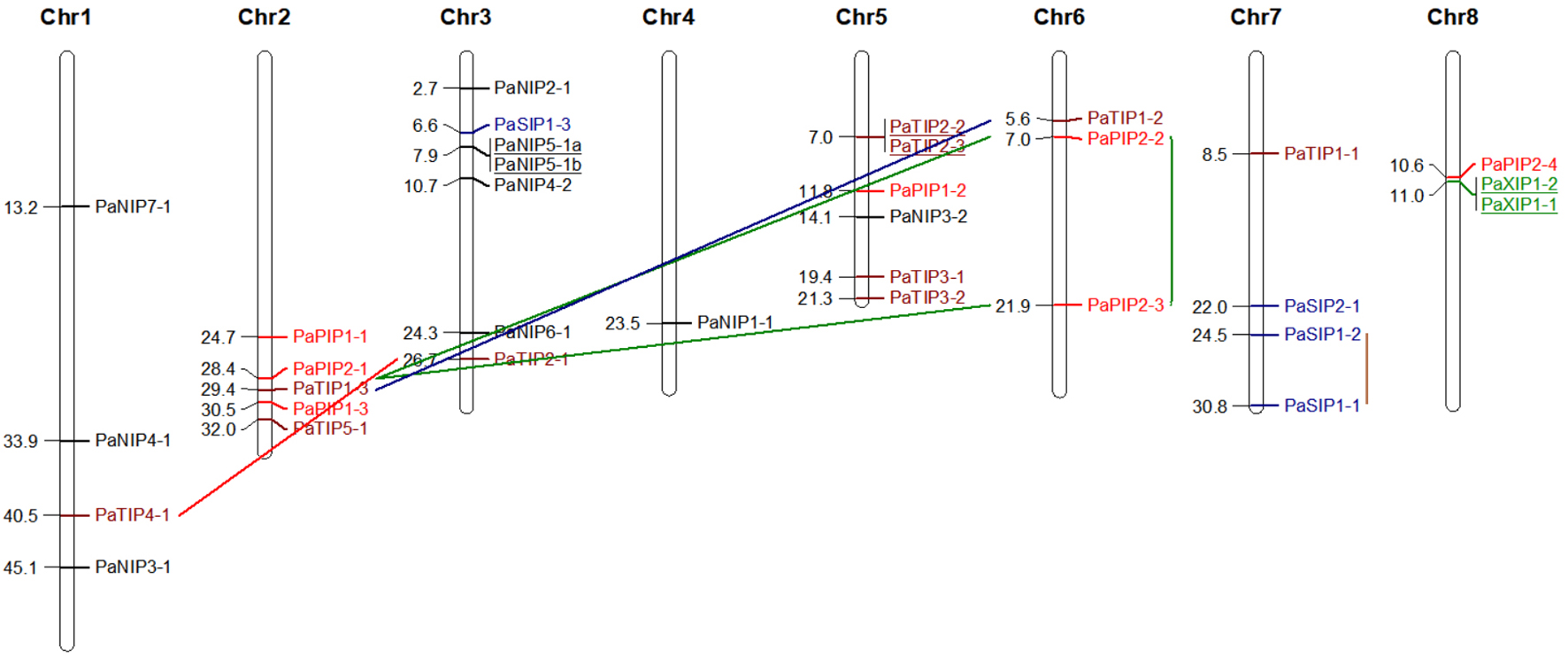

Supplement: Supplementary Figure 1 — Distribution of PaAQP genes on the eight P. armeniaca chromosomes. Thirty-three PaAQP genes are distributed across all eight P. armeniaca chromosomes. The right side of the chromosomal bar represents the PaAQP genes, and the corresponding positions on the chromosome (megabase pairs; Mb) are given on the left side. The PaNIP genes are in black, the PaPIP genes are in red, the PaSIP genes are in purple, the PaXIP genes are in green, and the PaTIP genes are in magenta. Tandemly duplicated genes are underlined. Whole genome/segmental duplications are lined and underlined. [file Image_1.jpg]

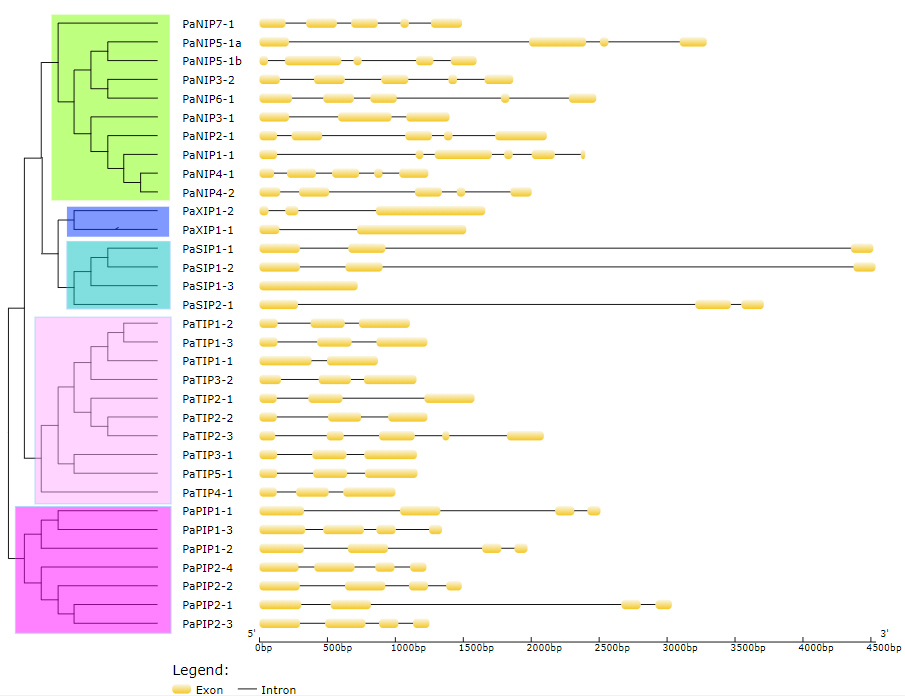

Supplement: Supplementary Figure 2 — Exon-intron structures of the 33 PaAQP genes. The yellow boxes denote exons, and the lines denote introns. The exon-intron structure was determined via GSDS 2.0 (http://gsds.cbi.pku.edu.cn/) on the basis of gene and CDSs. [file Image_2.jpg]

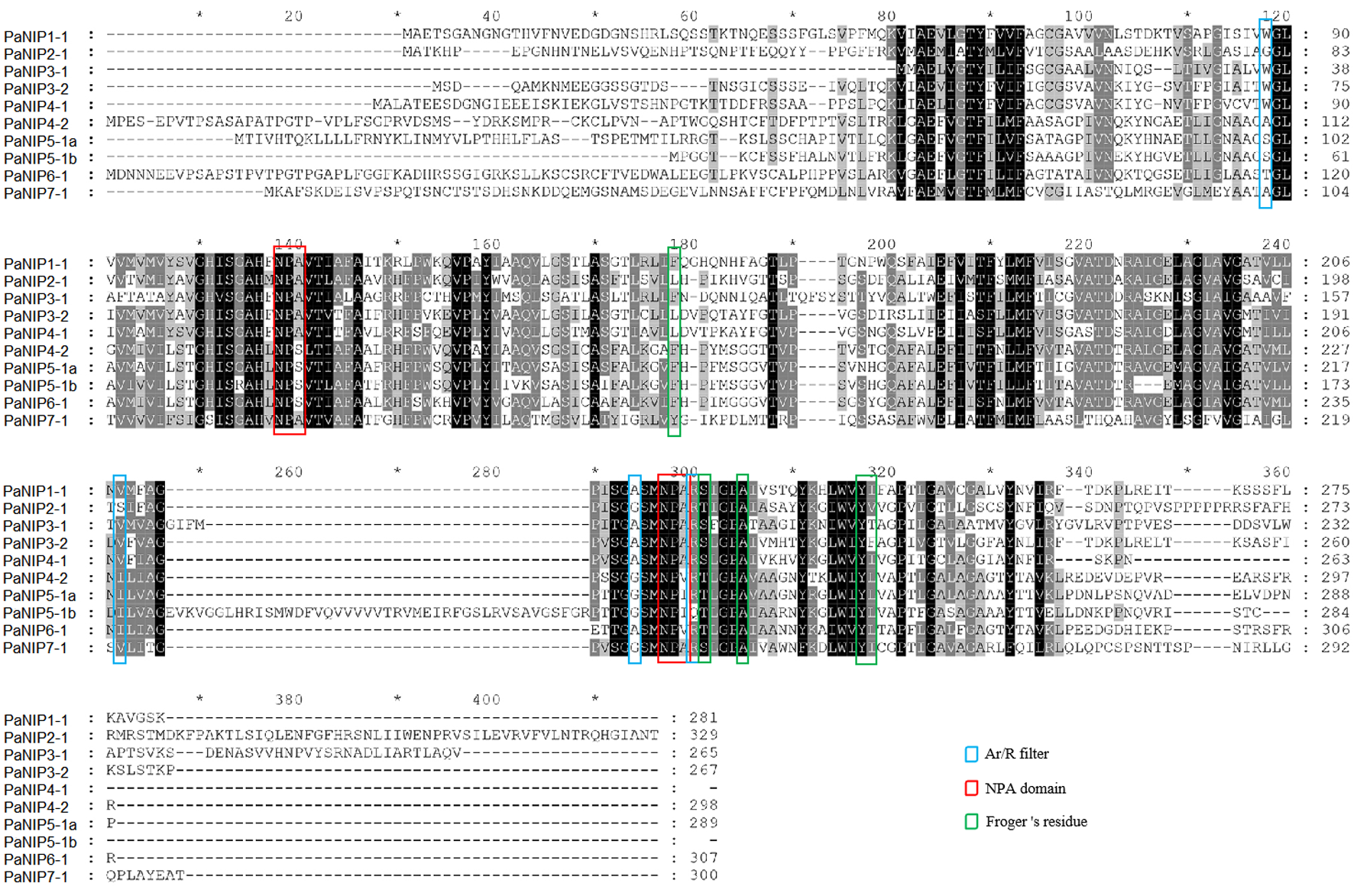

Supplement: Supplementary Figure 3 — Protein sequence alignment of PaNIPs identified in P. armeniaca showing the amino acids at NPA domains, ar/R filters, and Froger’s residues. [file Image_3.tif]

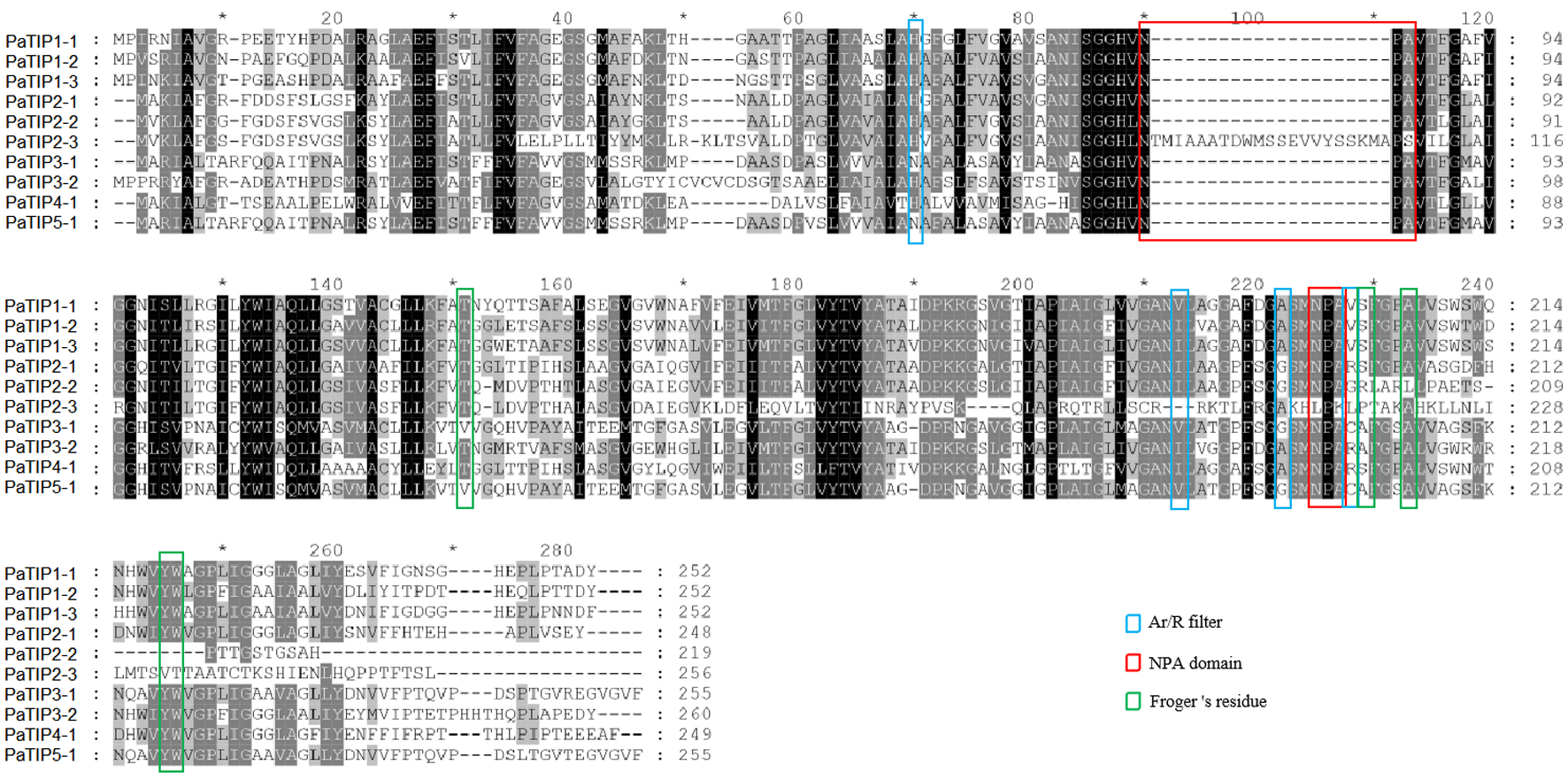

Supplement: Supplementary Figure 4 — Protein sequence alignment of PaTIPs identified in P. armeniaca showing the amino acids at NPA domains, ar/R filters, and Froger’s residues. [file Image_4.tif]

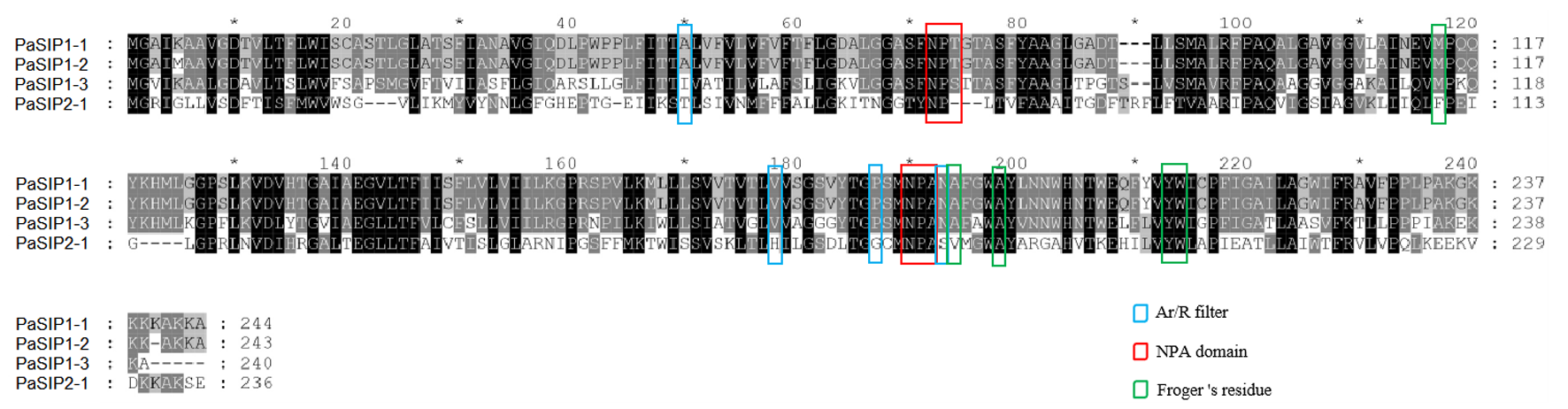

Supplement: Supplementary Figure 5 — Protein sequence alignment of PaSIPs identified in P. armeniaca showing the amino acids at NPA domains, ar/R filters, and Froger’s residues. [file Image_5.tif]

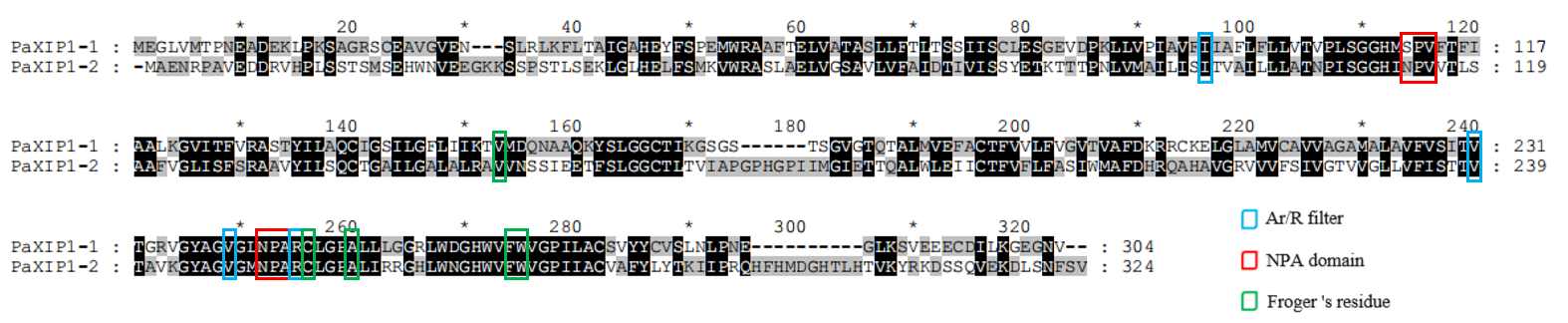

Supplement: Supplementary Figure 6 — Protein sequence alignment of PaXIPs identified in P. armeniaca showing the amino acids at NPA domains, ar/R filters, and Froger’s residues. [file Image_6.png]

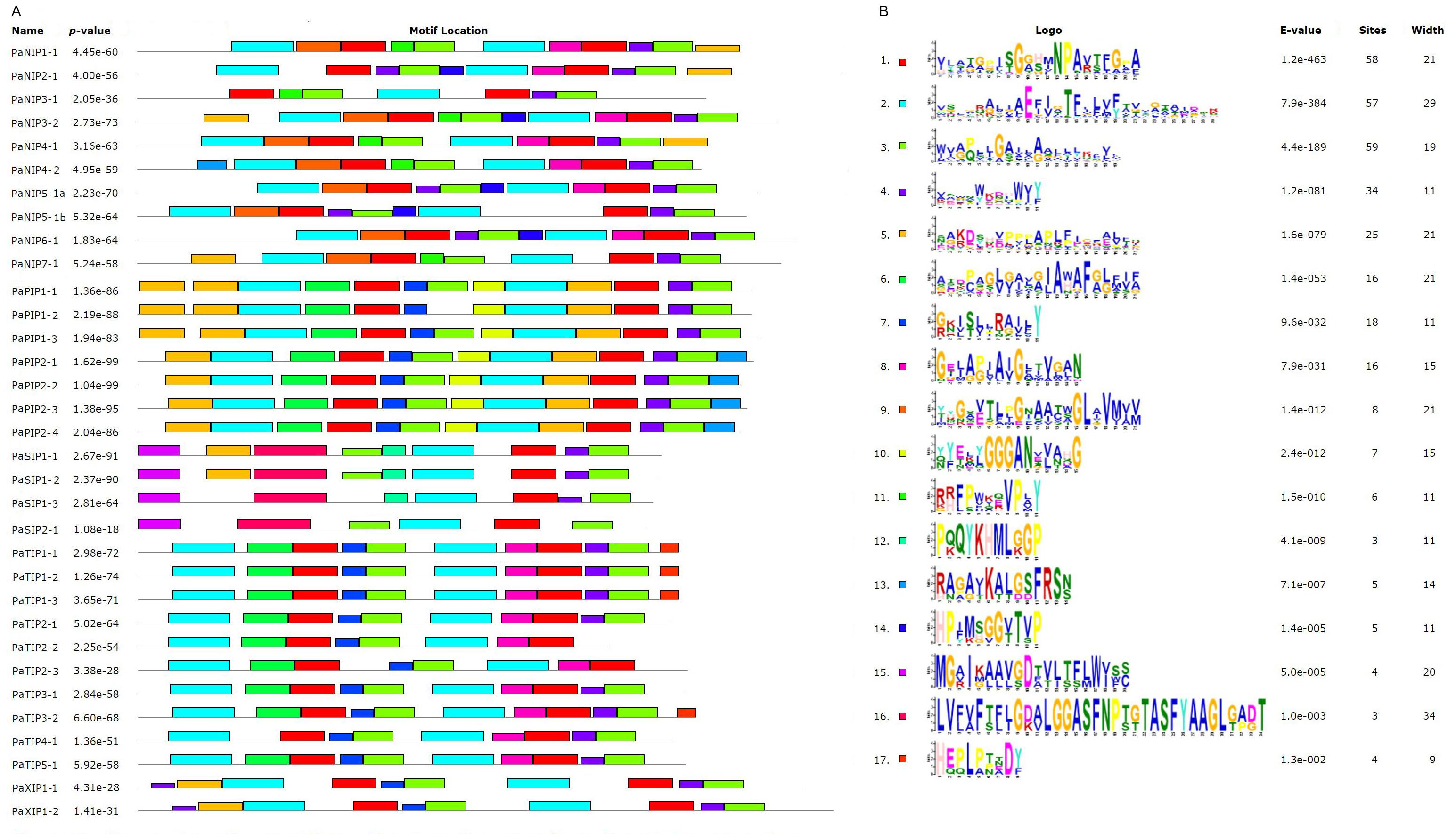

Supplement: Supplementary Figure 7 — Distribution of conserved motifs in the PaAQP proteins. The motifs were identified via the online MEME program. The different colored boxes indicate different motifs at the corresponding positions. [file Image_7.jpg]

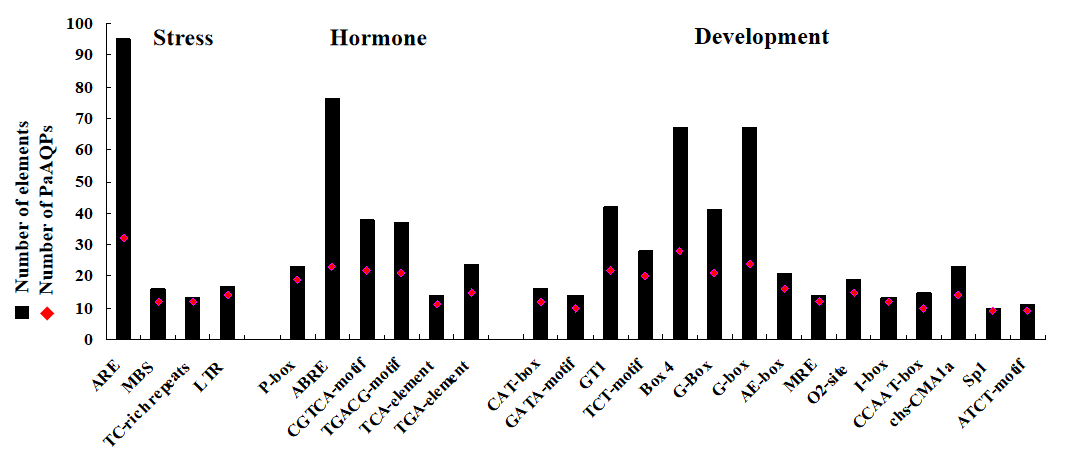

Supplement: Supplementary Figure 8 — Various cis-acting elements in the promoter of PaAQP genes. The numbers of ARE (AAACCA), ABRE (ACGTG), MBS (CAACTG), MRE (AACCTAA), and other cis-acting elements in the promoter region of P. armeniaca PaAQP genes from each AQP family. The cis-acting elements were determined in the 2-kb promoter region upstream of the translation initiation site using the PLACE database. [file Image_8.png]

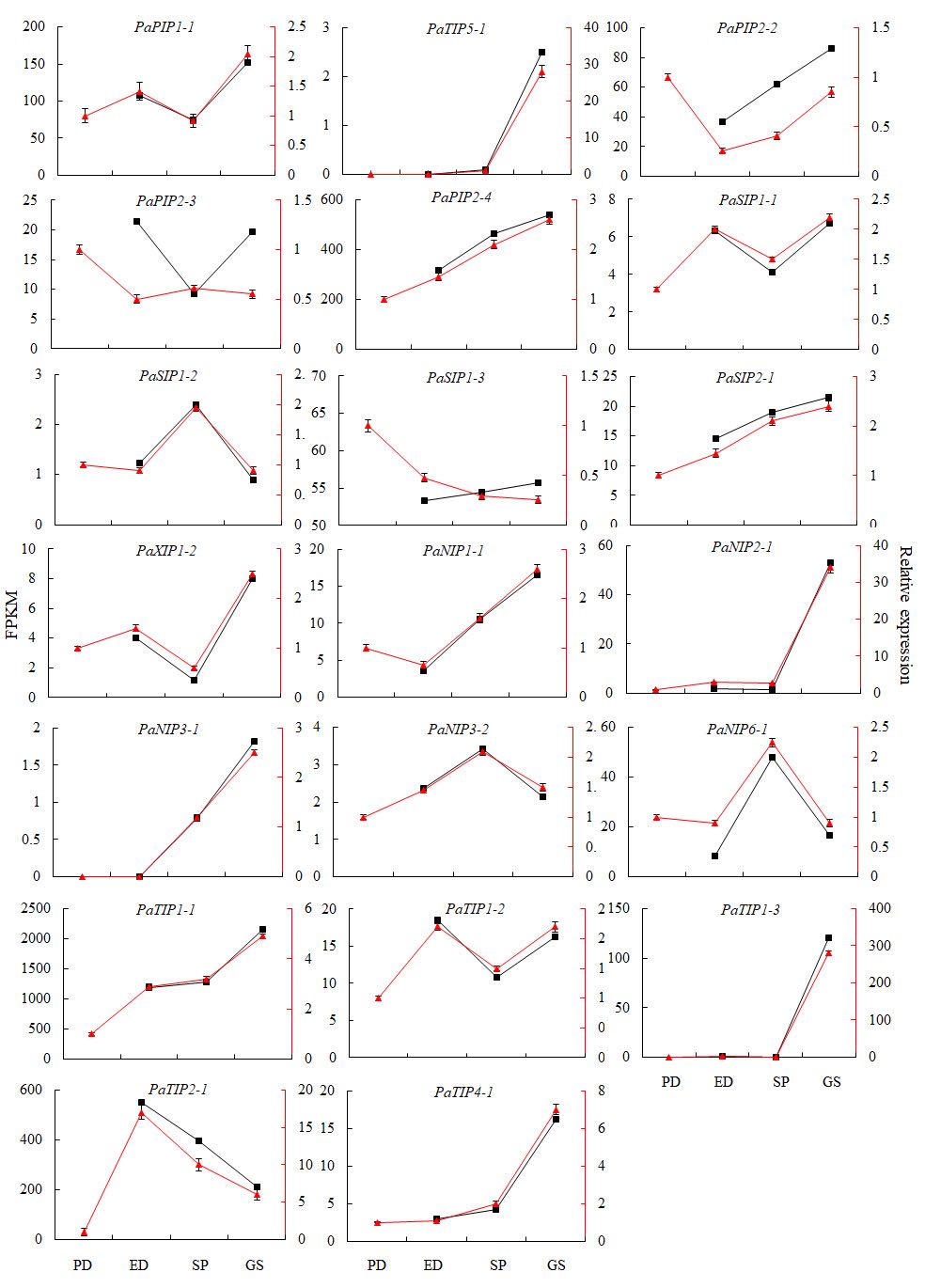

Supplement: Supplementary Figure 9 — qRT-PCR analysis of the relative expression of 20 genes during the dormancy and sprouting stages of P. armeniaca. [file Image_9.jpg]
