# Supplementary material for: Genome-Wide Identification and Function of Aquaporin Genes During Dormancy and Sprouting Periods of Kernel-Using Apricot (Prunus armeniaca L.)
Source: Front Plant Sci. 2021 Oct 4;12:690040. doi: 10.3389/fpls.2021.690040 (PMC8520955; doi:10.3389/fpls.2021.690040)
Supplement: Supplementary Table 2 — The coding regions of WRKY6, TSPO, and ATHATPLC1G can interact with PaAQP genes. [file Table_2.doc]

[**Table S2**](https://www.ncbi.nlm.nih.gov/pmc/articles/PMC3510131/figure/fig1/) **The coding regions of *WRKY6*, *TSPO*, and *ATHATPLC1G* can interact with *PaAQP* genes.**

*WRKY6*

>PaLWMG0404022400.01.T01 predict

ATGTTTCCGGGTATTGAATTCCCCGTCAAACTTGGCGGCAGGGAAGACCAGCTGGCTGTTCCACAGCCATCCATTCATGACAACAATAATCGTGTGGTTGTGGATGAGGTCGACTTCTTTTCCGATCGTAAGAATAAGCACAATACAACCACCACTGATGATCATCATCAGGATATGAAATCCAACGGTACAATCAGCGTCAAGAAGGAGAATTGGACTGGCTTGGATGTAAATACTGGCCTGCACCTTGTTACTGCTAACACCGGAAGTGATCAGTCAATGGTTGATGATGGGATTTCATCCGATGTGGATAACAAAAGAGCAAAGAATCATGAGCTGGCACAGTTGCAAGTGGAGCTCCAACGTATGAATTCTGAAAATCTGAGGCTGAAAGAAATGCTTGGTCAGGTGACCAACAACTACAGTGCTCTGCAGATGCATGTTGCGGCTGTAATGCAACAGCAGCAGCAGCAAAATCACACAGCCGTGGCTGATCAAAGCTCTCAATTAAACCATGATCAGAATGTTGAAGCAAAGTCTGATCAAGAGAAGAAACAAGGGTTGGTGCCAAGGCAGTTTCTAAACTTGGGCCCCAGAGCCACAGCCGAGACCGATGACCAAGTTTCCAATTCTTCATCAGAAGCCAGAACCCGATCGGCTTCACCTCGGAACATTAATGAAGCTGCATCATCAAAAGATCATCACCTGAAGAAAAATGATCATCCGATTGGTCCATTGGATCCCGAAAACTCTAACAATTTCAGGGATGGTAAAAGGGTTGGAAGGGAAGAGAGTCCTGAATCGGAATCACAAGGCTGGGTGCCCAACAAGGCTCCTAAGCTCAGCAACTCTGCAGCTAATAAGCCAATTGATCAATCCACCGAGGCCACCATGAGGAAAGCCCGCGTATCGGTTCGAGCCAGATCAGAAGCTCCCATGATTACTGATGGATGTCAATGGCGAAAATACGGACAAAAGATGGCAAAAGGAAACCCATGTCCTCGTGCATATTATAGGTGTACCATGGCTGTTGGTTGTCCTGTTCGCAAACAAGTACAACGTTGTGCTGAGGACAGAACCATATTGATCACAACATATGAAGGCAACCATAACCACCCTCTGCCTCCAGCTGCAATGGCCATGGCATCAACCACAACAGCAGCAGCAAGCATGTTGCTTTCGGGCTCCATGTCAAGCGCAGATGGGATAATGAACCCAAACAATTTGCTAGCCAGAGCAATTCTTCCATGCTCATCAAGTGTGGCAACAATCTCAGCCTCAGCTCCTTTCCCTACTGTCACATTGGACCTCACCACCACCCCATTACAGTTCCAAAGACCACAGTCCCAATTTCAAGTCCCTTTCCCAGGGCAACAGCCACAACTGCCTCAAGTTTTTGGACAGGCCCTTTATAACCAATCCAAGTTTTCTGGGCTTCAGCTATCCCAGGATTTAATGGGGTCTAATTCCCAACAGCAGCAGCAACATTTGCCGCATCAAACTCAGTCTGCCTCGTTCGCTGACACAGTGAGTGCCGCTACAGCTGCCATAACTGCCGACCCAACTTTCACTGCTGCTCTTGCGGCCGCCATCACCTCCATCATCGGTGGAGGTCATCCGAACAACAACAACAACAGCGCCTCGACGACCTCCAACAACAGCAATGGAGGCAACAACAACAGCAACAGCAAAATGAGCGGCTTCCCAGGACATTAA

Primers:

WRKY6-AD-F： TATGGCCATGGAGGCCAGTGAATTCATGTTTCCGGGTATTGAATTCC

WRKY6-AD-R： TCTGCAGCTCGAGCTCGATGGATCCTTAATGTCCTGGGAAGCCGCT

*TSPO*

>PaLWMG0809736000.01.T01 predict

ATGGATTCCCAGAACCTCAAACAACGCAGAACAGACGATCCCGACACCGCTGCAGCCACCACCACCATGAGCAAGAGCAACTACAAAAGCAAAAAGGATATGAGAATGGACATGGCCAAACGCGGCCTTAGGTCATTGGCCATAGCGGTTGCAATCCCTGTCTCTCTCCACCTGCTTGCTATTTACTTGGGCTCCACCGACAACTACCCCACCGGCTCCTCCAAGCCCTTCTGGTTCCCGCCCTTGTGGGCCCTGCGCTTCATTTGCATGGCTTCCAGTTTCTTGATGGGTCTCTCGGCTTGGCTTGTGTGGGCTGACGGTGGCTTCCACAAGAATCCCACGGCTCTGCCTCTTTATCTGGCCCAGCTAGGATTGAATTTGATTTGGGATCCGATTGTGTTCGGGGCGGGAGCGCCTTGGGTCGGGTTGCTGGTGTCCATGGGGATGTTTGGGACTATGGTTGCCTGCACTCGGGTGTTCAAGAGTATCAATCCAGTTGCAGGTGATCTGATGAAGCCCACTTTGGCATGGGTTGCCTTTTTGGCCATTGTAAATCTTAAGCTTGTGTTCCATTGA

Primers:

TSPO -AD-F： TATGGCCATGGAGGCCAGTGAATTCATGGATTCCCAGAACCTCAAAC

TSPO -AD-R： TCTGCAGCTCGAGCTCGATGGATCCTCAATGGAACACAAGCTTAAG

*ATHATPLC1G*

>PaLWMG0202254500.01.T01 predict

ATGGGGGATAGCAGCTACAACTACAAGATGTTCAAGTGCTTCAACAGGAAGTTCAAGGCCAGCGAGGGGGGCCCACCGCCCGACGTCAAGGCCATCTTCTCCAAGTTCGCGCAGGATGGGGACTTCATGTCCGTGGACCAGTTCCGGAGCTTTCTGGTGGAGCACCAGGGCGAGCCGGCCGACGCCATCACCCTCTCCGACGCCCACCGCATCCTCCAGGAGTTCTCGCGGAGCCACCCGGGCGAGGCCGCCCACCACCACCAGCACGTGCGGGGCCTCACCCTTGAGGATTTCTTCAATTTCCTGTTTCTAGAAGAACCCATGAAAAAACAGATACACCATGACATGACTGCTCCAATATCACATTACTTCATATACACAGGGCACAATTCCTACCTTACTGGGAATCAACTTAGTAGTGACTGTAGTGATGTCCCAATCATCAAGGCATTGGAAAAAGGTGTCAAAGTTATTGAACTTGATTTATGGCCAAATTCTGCCAAAGATGATGTTCAAGTTCTTCATGGAAGGACCTTGACCACTCCTGTAACATTGGTCAAATGCTTGAAGTCCATAAAAGAGCATGCTTTTGTTAAATCTCCATACCCCGTCGTTATTACTTTCGAAGACCACCTTACTCCCAAGCTTCAGGCTAAAGTTGCAGAGATGGTTATCCAAACATTTGGAGACACGCTGTATTATCCGGAGGCAGAAGACCAGATGGTAGAGTTCCCATCACCTGAATCTTTAAAAAATCGAATTATTATCTCAACAAAACCACCAAAAGAAGGCAGTGGATCAAAGGATTCATCTGAAGAAGATGAAAAGAGTGGGAGTGATGGTGAGCCAGCTATTGACAAGGCCAATGCAGCCGAAGCCAAATCATTCCAACAATCAGCACCTGAGTACAAACGTCTTATAACAATTCATGCTGGAAAACCTTCGGGTGAATTGAAGGATGCTCTTGCTGTTGGTGATAAAGTTCGACGTCTTAGTTTGAGCGAACAGAAACTTGAAAAGGCTGCTGAGGATCACGGAACTGATGTCCTAAGGTTCACAAGTAAGAATATTCTAAGGGTGTACCCGAAAGGAACTCGATTCACCTCTGGGAATTACAAACCACATGTTGGGTGGATGCATGGAGCTCAAATGGTTGCATTTAATATGCAGGGAAATGACAAATACAATTGGTTGATGCATGGGATGTTTAGAGCCAACGGAGGATGTGGTTATGTAAAAAAGCCTGACTTTTTGATGCACAAGGGTCCAAATGATGAGGTTTTTGATCCTAAAAAGACCTTGACAGTGAAAAAGACATTGAAGGTAAAAGTATACATGGGAACTGGATGGCACTTGGATTTTAGCCGAACACACTTTGATTCCTTCTCCCCACCAGACTTTTACACAAAGGTTTATATTGTTGGAGTGCCAGCTGATTGTGGCAAGCAAAAAACAAAGATAATTGAGGATGAATGGATACCTGTTTGGGATGAAGAGTTTACATTCCCTCTAACCATTCCAGAGCTTGCCATACTTCGAATCGAAGTTCGAGAGTATGACAGGTCTGAGAAAGATGACTTTGGCGGGCAAAACTGTTTGCCCGTCTCCGAGCTAAAGCCAGGGATTAGGGCAGTGCCCCTTTATGATAAAAAGGGAGAGAAATTCAAATCTGTAAAGCTTCTAATGCGGTTTCAGTTTGTGTGA

Primers:

ATHATPLC1G-AD-F :TATGGCCATGGAGGCCAGTGAATTCATGGGGGATAGCAGCTACAAC

ATHATPLC1G-AD-R:TCTGCAGCTCGAGCTCGATGGATCCTCACACAAACTGAAACCGCA
