# Supplementary material for: Genome-Wide Identification and Function of Aquaporin Genes During Dormancy and Sprouting Periods of Kernel-Using Apricot (Prunus armeniaca L.)
Source: Front Plant Sci. 2021 Oct 4;12:690040. doi: 10.3389/fpls.2021.690040 (PMC8520955; doi:10.3389/fpls.2021.690040)
Supplement: Supplementary Table 3 — Primers used in the qRT-PCR analysis of the PaAQP genes, and cold resistance genes coexpressed with PaAQP genes. [file Table_3.docx]

**Table S3 Primers used in the qRT-PCR analysis of the *PaAQP* genes, and cold resistance genes coexpressed with *PaAQP* genes.**

| **Gene name** | **Gene code** | **Forward primers (5'→3')** | **Reverse primers (5'→3')** |
| --- | --- | --- | --- |
| *PaPIP1-1* | PaLWMG0202050500.01 | CTGTTTGAGCCTGGTGAGC | CAAGCAATGCCCTGAATA |
| *PaPIP2-2* | PaLWMG0605030800.01 | GGCTCGCAAGGTTTCAC | CTCCACCGCCATACTCC |
| *PaPIP2-3* | PaLWMG0605338300.01 | AGCCAATCTGATGGAGAC | TGACCAGTGACACCTTCCTAG |
| *PaPIP2-4* | PaLWMG0806733800.01 | CTGCCTATTGGGTTTGC | TCAGTTGGTTGGGTTGC |
| *PaSIP1-1* | PaLWMG0706367400.01 | TTGTAATCATACTCAAGGGTCC | GGGGCAAATCCAATAAAC |
| *PaSIP1-2* | PaLWMG0706293200.01 | TTGTAATCATACTCAAGGGTCC | GGGGCAAATCCAATAAAC |
| *PaSIP1-3* | PaLWMG0302672200.01 | GCAGTTCTGACTTCCCTG | ACCCAAAACCTTGCCTAT |
| *PaSIP2-1* | PaLWMG0706248900.01 | TCTTCTTTGCCTTGTTGG | CCCTGGGATGTTTCTGG |
| *PaNIP1-1* | PaLWMG0403890000.01 | ACCAACCAAGAATCAAGC | ACCATCACAACCAGTCCC |
| *PaNIP2-1* | PaLWMG0302537400.01 | CAGAGCCTGGAAACCACAAC | TCATCTCCGCCATCACCTTT |
| *PaNIP3-1* | >PaLWMG0101288800.01.P01 | GTTTTGGCCCTGCTACTGTC | ACCCTGAGAACACCGTACAC |
| *PaNIP3-2* | PaLWMG0504454700.01.P01 | GGAGCTTTCCATCCGTACATG | CAGCAGTGACAACAAACAGGA |
| *PaNIP6-1* | PaLWMG0303063200.01 | ACCAGAAGACACAAGGCTCA | GGTGACAGATGGGTTGAGGT |
| *PaTIP1-1* | PaLWMG0705957400.01 | GTAGCCGTCAGTGCCAACA | GAGCAAATGCGGAGGTAG |
| *PaTIP1-2* | >PaLWMG0604986400.01.P01 | GCTTGCTTGCTTCTCAGGTT | CACAATCTCCAGCACCACAG |
| *PaTIP1-3* | >PaLWMG0202209500.01.P01 | GGTAAGGAAACGGCAGCATT | ACACTGTGTAAACCAAGCCG |
| *PaTIP2-1* | PaLWMG0303130000.01 | TGTCCTCACTGGCATCTTC | GGATGGGAATTGTCAAGC |
| *PaTIP4-1* | >PaLWMG0101145200.01.P01 | GTGAGCTGGAACTGGACTGA | TGGGGAGATGAGTTGTTGGT |
| *PaTIP5-1* | >PaLWMG0203269600.01 | TCTCAAATGGTGGCCTCTGT | CCTTCCAAAACTGATGCCCC |
| *PaXIP1-2* | PaLWMG0806748700.01 | TGTTGAGGAAGGGAAGAAG | TTAGTGGCAAGGAGGAGAA |
| *PaElf* | PaLWMG0403535600.01.T01 | ATTGGAGGTGGAGAGTTCGG | TGCAAGAGTCCTCCCATCC |
| *PaEREBP* | PaLWMG0201851100.01 | CCCTTTCATGGCTCCTCTGA | AGGCTGTTGTTGTTGTTGCT |
| *PaZFC3HC4* | PaLWMG0201851100.01 | CTGTTGCCTGATCCAATGGG | GCTGCCAGTTTTGTGTCTCA |
| *PaZFCHY* | PaLWMG0705957200.01 | CGCCCATTATAGACGGAGGT | ACCCTTTTCACATCATGGCG |
| *PaSGBP* | PaLWMG0203256100.01 | TGTGGAGCAAGCTTTCATGG | GTTGTCCTCGGATCTGCACT |
| *PaUbI* | PaLWMG0303274500.01 | GCTTGACGCCAATCTACAACA | GGCTTCCCAAGTTTGCACAA |
| *PaWRKY* | PaLWMG0605388600.01 | AACACCAGCCCACCTATGAA | GGCACCACATCTTGAAGCAA |
| *PaFAD* | PaLWMG0302976300.01 | GTTTCAAGCTCCCCAAGTGG | GGGCTATGTGGGTCTCTCTC |
| *PaMYB* | PaLWMG0202529800.01 | AAAGTTGTGCAAGGCCGATT | TTCTCCATCCAAGCCTCCTG |
| *PabZIP* | PaLWMG0201730400.01 | GCGAATGTGGCATCTCAGTT | TGGTATAGGTTGGCTTGGCA |
| *PaPRE* | PaLWMG0807000300.01 | AGAGGAATTGGCTGTTGGGT | CTCACGTTTGGGATTTGGCA |
| *PaAUX* | PaLWMG0403933100.01 | TCGGCTTATGATCTCCCACC | CTTCCCGCAGTTTGTGTTCA |
| *PaNAC* | PaLWMG0504758600.01 | GAGCAGTTCCAGAAGCAAGC | CCTCCTCCTTGTCCTTCTCC |
| *PaSer/Thr* | PaLWMG0405627800.01 | TCAAGACAGTCCCCAGGTTC | CAAGTTTGGTTAGTGCGGCT |
| *PaLEA* | PaLWMG0605060700.01 | ACCTCTCTGCCGACTTTGAT | CCCCAGAAAATGCCAGTGTC |
| *PaPIP1-3* | PaLWMG0202248600.01 | TGGGAGCCAACAAGTTTTCG | ATCCCAGCCCTGTAAAACGA |
| *AtActin* |  | GAGCTATATATTCGCACATGTACTCG | GATACAGAAGATTCGAGAAGCAGC |
