# Supplementary material for: Genome-Wide Identification and Function of Aquaporin Genes During Dormancy and Sprouting Periods of Kernel-Using Apricot (Prunus armeniaca L.)
Source: Front Plant Sci. 2021 Oct 4;12:690040. doi: 10.3389/fpls.2021.690040 (PMC8520955; doi:10.3389/fpls.2021.690040)
Supplement: Supplementary Table 5 — Predicted 3D structure and transmembrane region of the 33 PaAQP proteins. [file Table_5.doc]

**Table S5 Predicted 3D structure and transmembrane region of the 33 PaAQP proteins.**

| **Hit** | **Gene name** | **Gene code** | **Confidence (%)** | **Aligment Coverage (%)** | **3D Image** | **TM helix** |
| --- | --- | --- | --- | --- | --- | --- |
| [c2w2eA](http://www.sbg.bio.ic.ac.uk/phyre2/phyre2_output/9a51566108fba258/summary.html#c2w2eA_) | PaPIP1-1 | >PaLWMG0202050500.01.P01 | 100 | 85 | 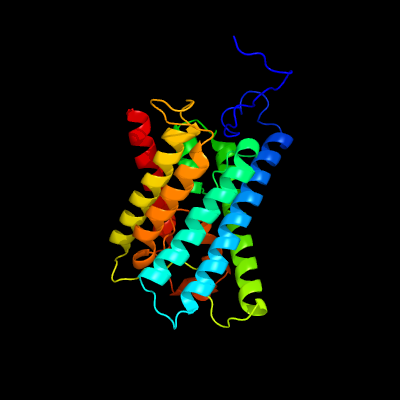 | 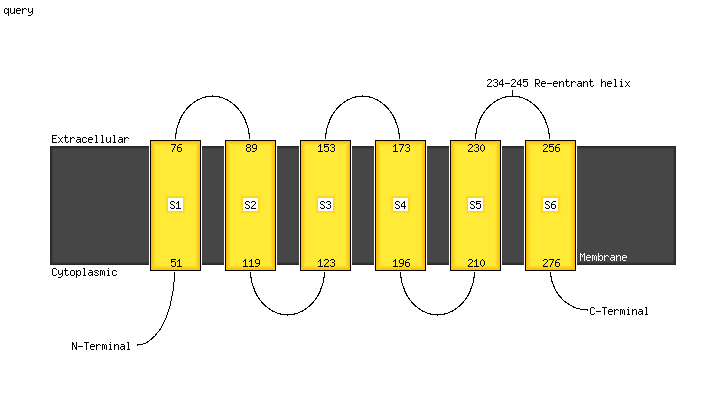 |
| [c2w2eA](http://www.sbg.bio.ic.ac.uk/phyre2/phyre2_output/3bd6f9913e8a4858/summary.html#c2w2eA_) | PaPIP1-2 | >PaLWMG0504379100.01.P02 | 100 | 86 | 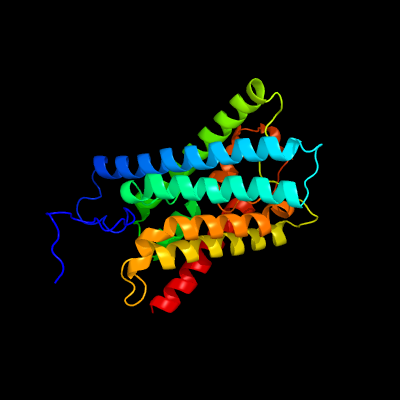 | 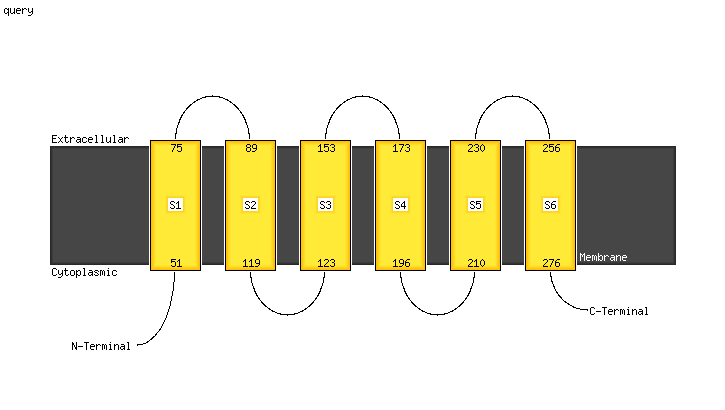 |
| [c2w2eA](http://www.sbg.bio.ic.ac.uk/phyre2/phyre2_output/2c8aa4d276298a12/summary.html#c2w2eA_) | PaPIP1-3 | >PaLWMG0202248600.01.P01 | 100 | 87 | 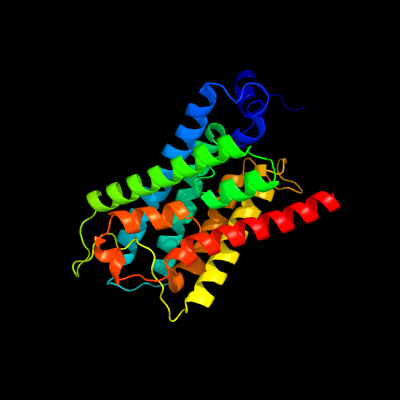 | 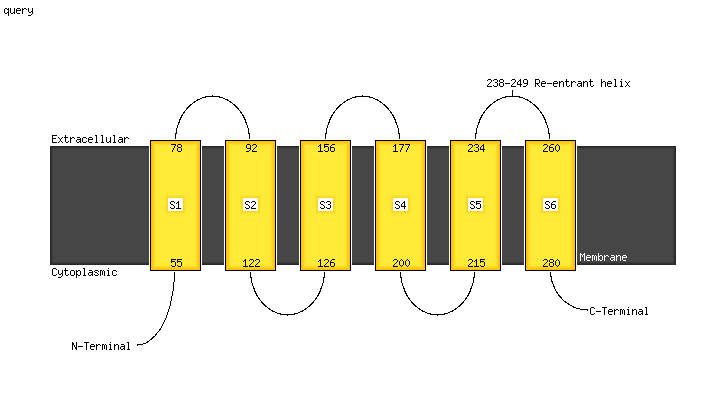 |
| [c2w2eA](http://www.sbg.bio.ic.ac.uk/phyre2/phyre2_output/5b360c3060701bfe/summary.html#c2w2eA_) | PaPIP2-1 | >PaLWMG0202173700.01.P01 | 100 | 88 | 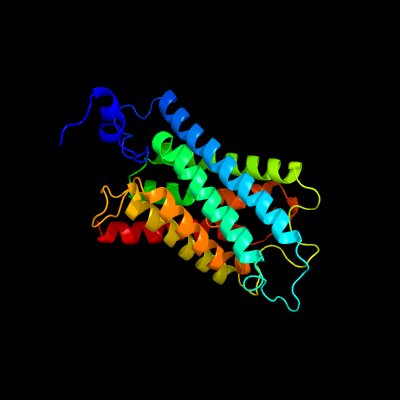 | 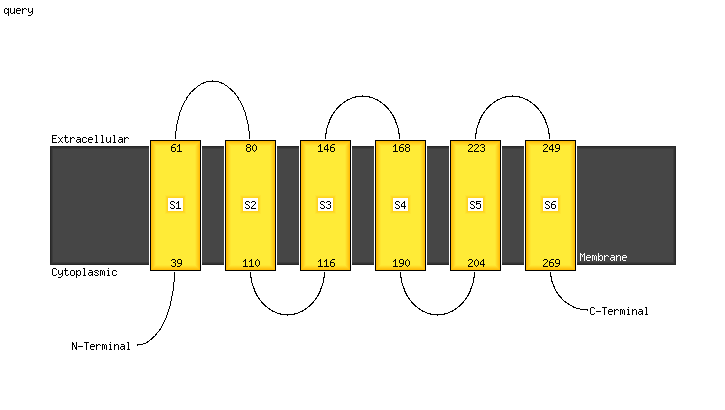 |
| [c2w2eA](http://www.sbg.bio.ic.ac.uk/phyre2/phyre2_output/dc29c95733700b32/summary.html#c2w2eA_) | PaPIP2-2 | >PaLWMG0605030800.01.P01 | 100 | 88 | 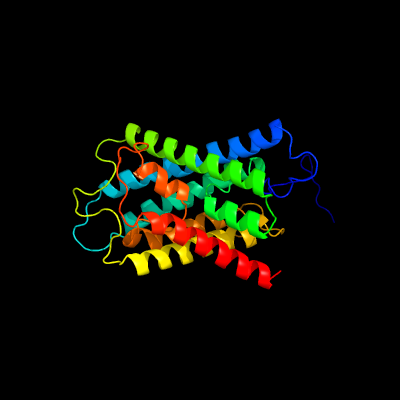 | 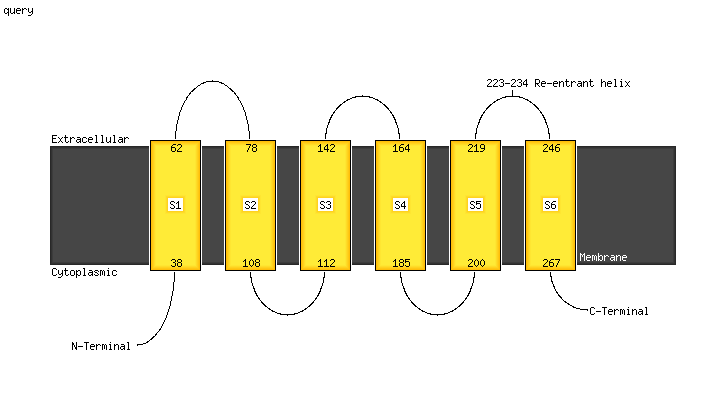 |
| [c2w2eA](http://www.sbg.bio.ic.ac.uk/phyre2/phyre2_output/513d88cd79b0df41/summary.html#c2w2eA_) | PaPIP2-3 | >PaLWMG0605338300.01.P01 | 100 | 87 | 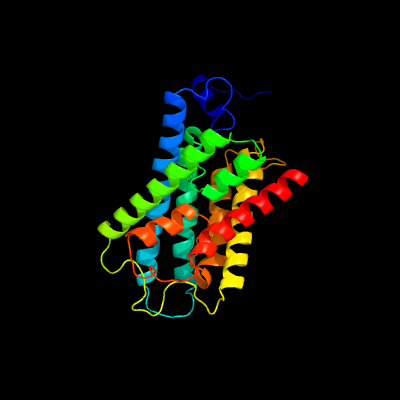 | 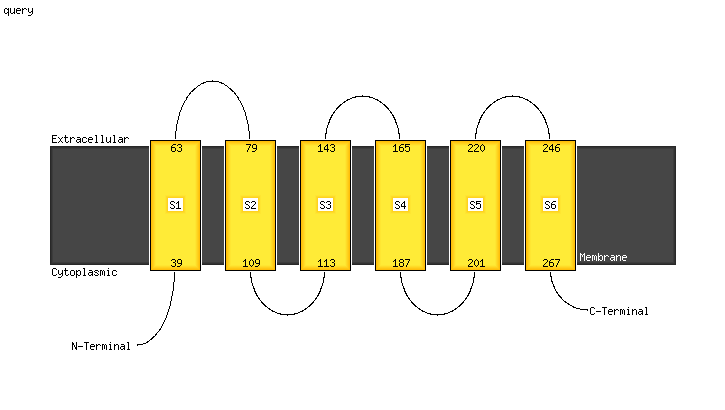 |
| [c2w2eA](http://www.sbg.bio.ic.ac.uk/phyre2/phyre2_output/2bd405bb986b878f/summary.html#c2w2eA_) | PaPIP2-4 | >PaLWMG0806733800.01.P02 | 100 | 88 | 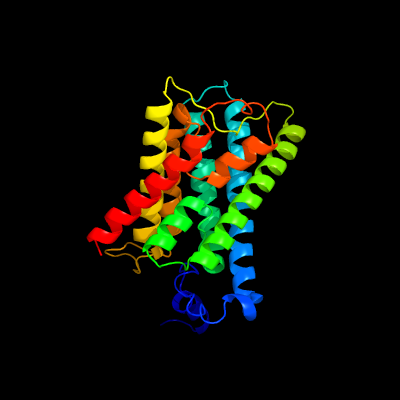 | 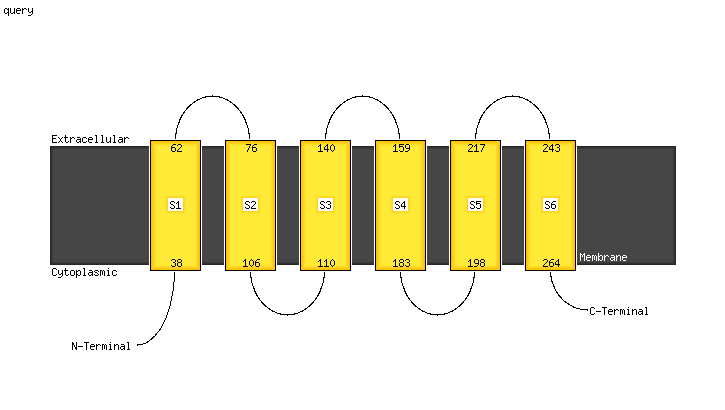 |
| [c2f2bA](http://www.sbg.bio.ic.ac.uk/phyre2/html/flibview.cgi?pdb=c2f2bA_) | PaSIP1-1 | >PaLWMG0706367400.01.P01 | 100 | 93 | 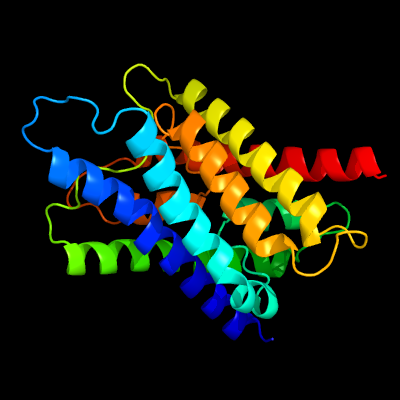 | 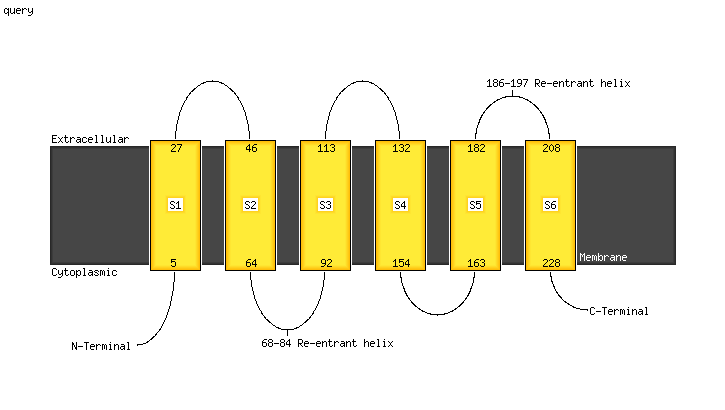 |
| [c2w2eA](http://www.sbg.bio.ic.ac.uk/phyre2/phyre2_output/41f1789285a9dd40/summary.html#c2w2eA_) | PaSIP1-2 | >PaLWMG0706293200.01.P01 | 100.0% | 84% | 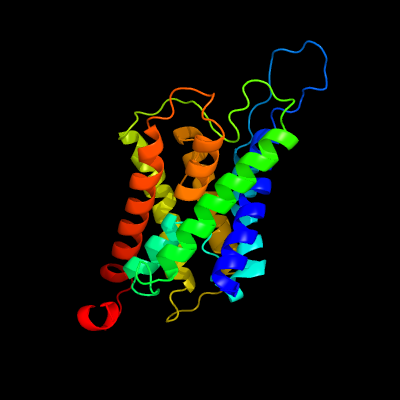 | 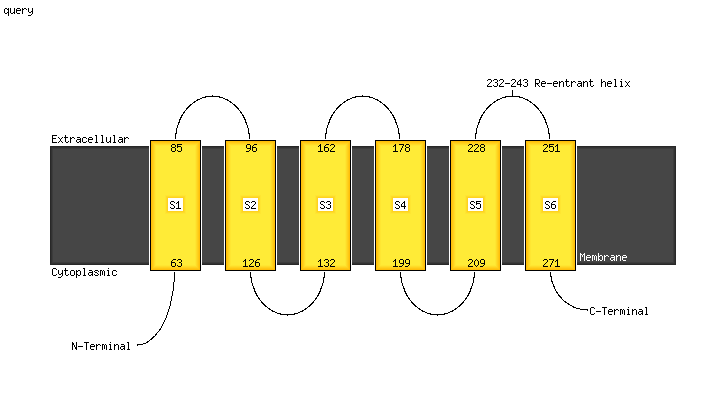 |
| [c2f2bA](http://www.sbg.bio.ic.ac.uk/phyre2/phyre2_output/ec20060960738b7d/summary.html#c2f2bA_) | PaSIP1-3 | >PaLWMG0302672200.01.P01 | 100 | 95 | 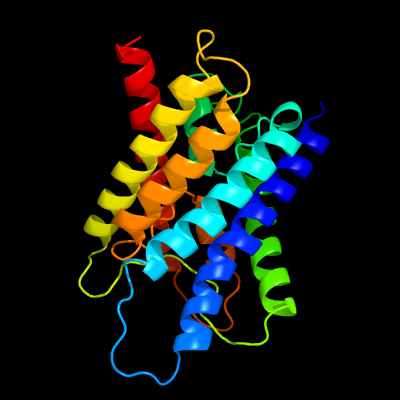 | 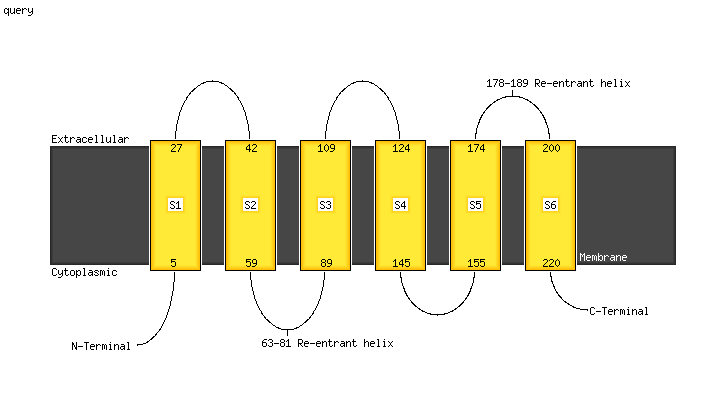 |
| [c2w2eA](http://www.sbg.bio.ic.ac.uk/phyre2/phyre2_output/e2c61ef5c10058aa/summary.html#c2w2eA_) | PaSIP2-1 | >PaLWMG0706248900.01.P01 | 100 | 94 | 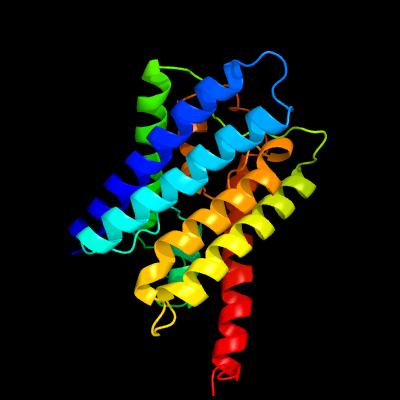 | 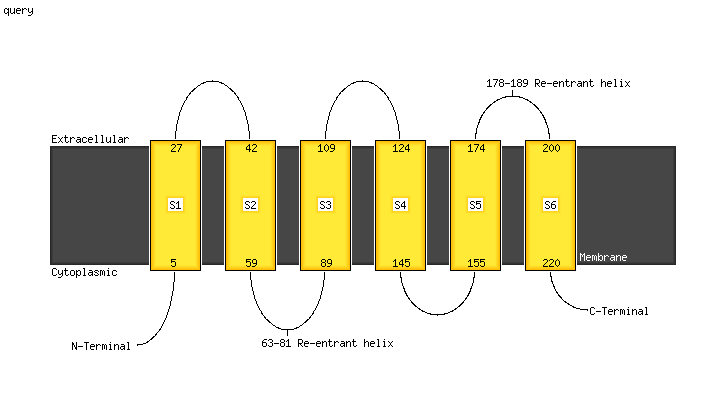 |
| [c6pojA_](http://www.sbg.bio.ic.ac.uk/phyre2/phyre2_output/3e426a524589ba7e/summary.html#c6pojA_) | PaXIP1-1 | >PaLWMG0806748900.01 | 100 | 80 | 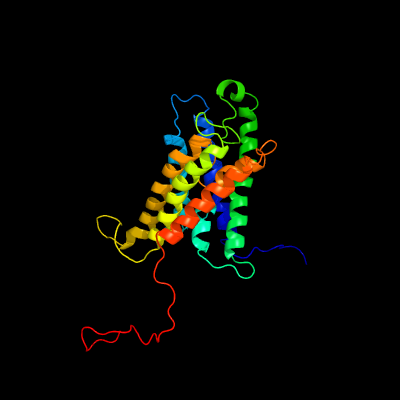 | 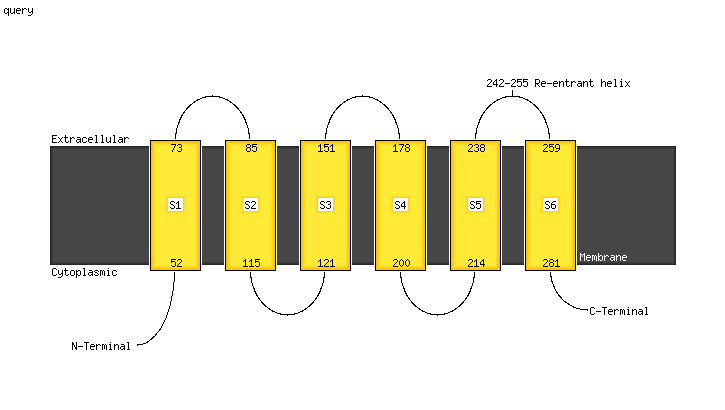 |
| [c2b6pA](http://www.sbg.bio.ic.ac.uk/phyre2/phyre2_output/0871985762963062/summary.html#c2b6pA_) | PaXIP1-2 | >PaLWMG0806748700.01.P01 | 100 | 89 | 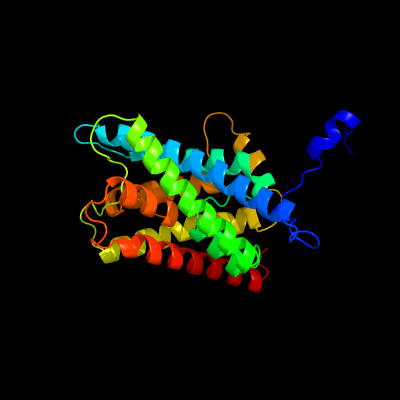 | 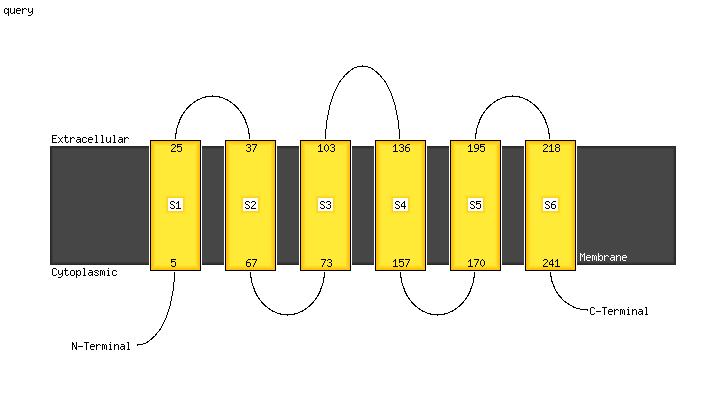 |
| [c2w2eA](http://www.sbg.bio.ic.ac.uk/phyre2/phyre2_output/3e06e1f15e798508/summary.html#c2w2eA_) | PaNIP1-1 | >PaLWMG0403890000.01.P01 | 100 | 88 | 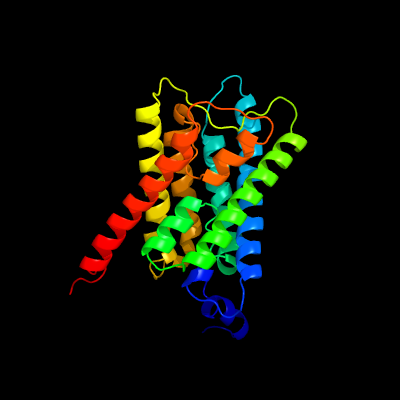 | 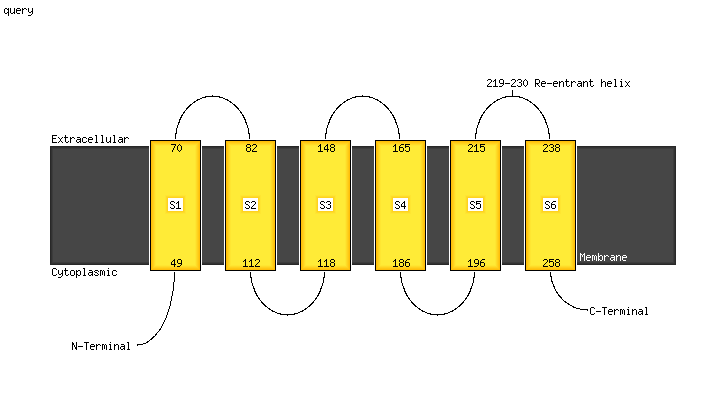 |
| [c2w2eA](http://www.sbg.bio.ic.ac.uk/phyre2/phyre2_output/c1c42d1be6c5e48c/summary.html#c2w2eA_) | PaNIP2-1 | >PaLWMG0302537400.01.P01 | 100 | 76 | 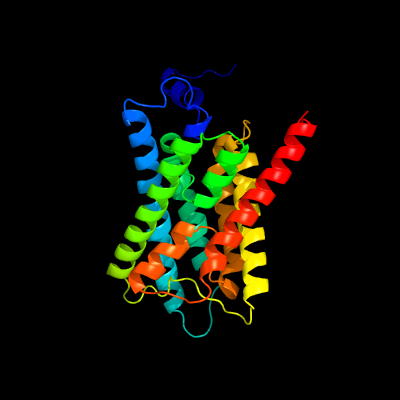 | 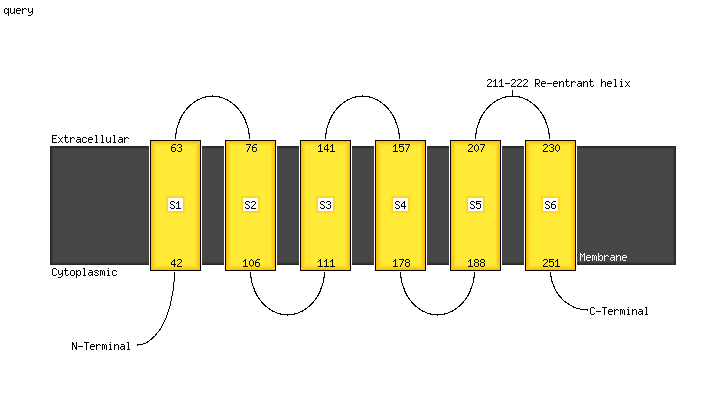 |
| [c2b6pA](http://www.sbg.bio.ic.ac.uk/phyre2/phyre2_output/9f6851e8d955c8a1/summary.html#c2b6pA_) | PaNIP3-1 | >PaLWMG0101288800.01.P01 | 100 | 93 | 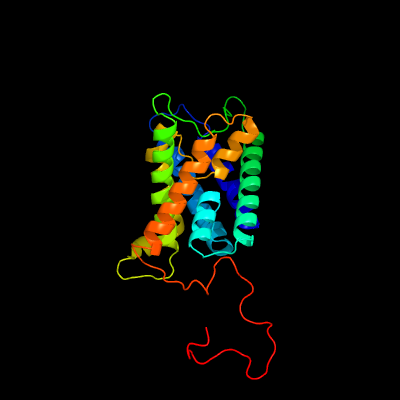 | 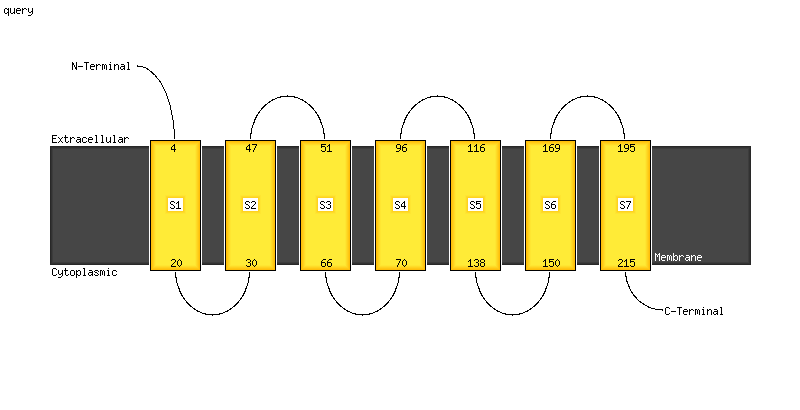 |
| [c5i32A](http://www.sbg.bio.ic.ac.uk/phyre2/phyre2_output/bc4a784d504378c7/summary.html#c5i32A_) | PaNIP3-2 | >PaLWMG0504454700.01.P01 | 100 | 83 | 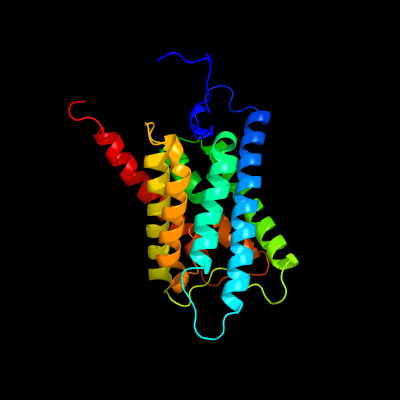 | 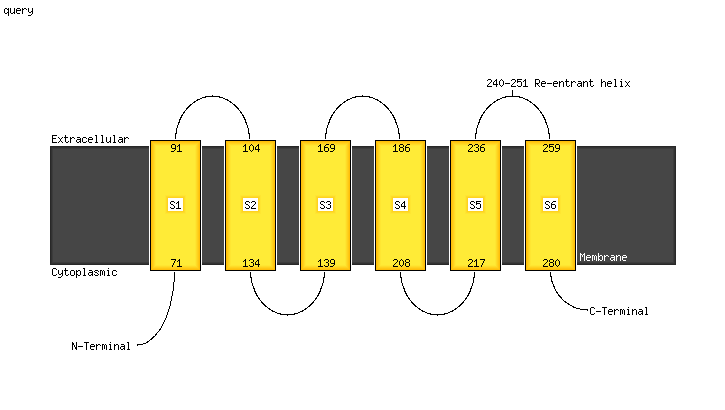 |
| [c2w2eA](http://www.sbg.bio.ic.ac.uk/phyre2/phyre2_output/390ff591889bee5a/summary.html#c2w2eA_) | PaNIP4-1 | >PaLWMG0100895200.01.P01 | 100 | 91 | 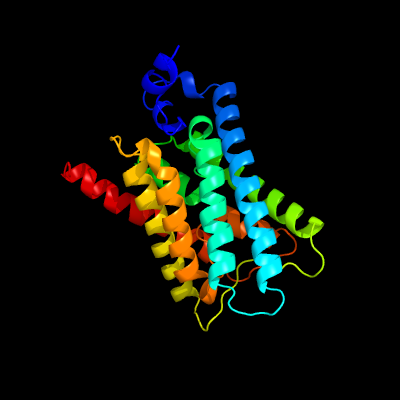 | 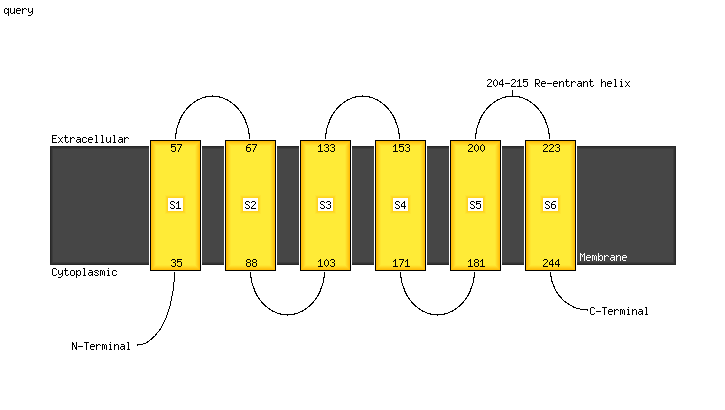 |
| [c2w2eA](http://www.sbg.bio.ic.ac.uk/phyre2/phyre2_output/5f8140416fdd7207/summary.html#c2w2eA_) | PaNIP4-2 | >PaLWMG0304019700.01.P01 | 100 | 93 | 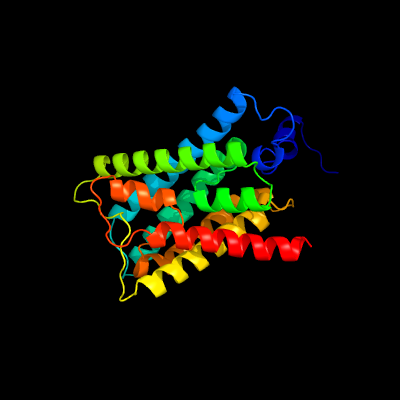 | 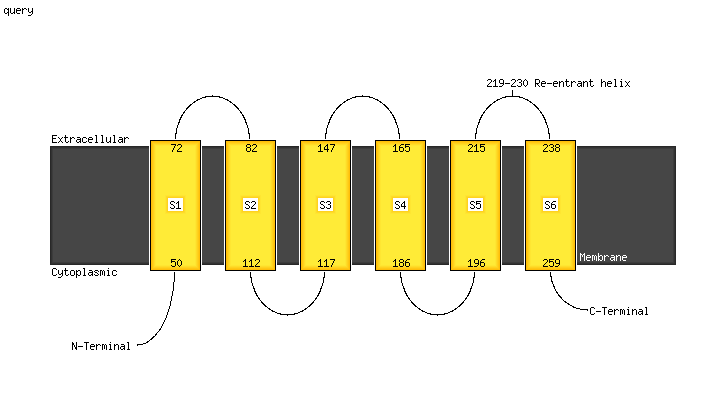 |
| [c2w2eA](http://www.sbg.bio.ic.ac.uk/phyre2/phyre2_output/47a60716237b3f23/summary.html#c2w2eA_) | PaNIP5-1a | >PaLWMG0303878600.01.P01 | 100 | 87 | 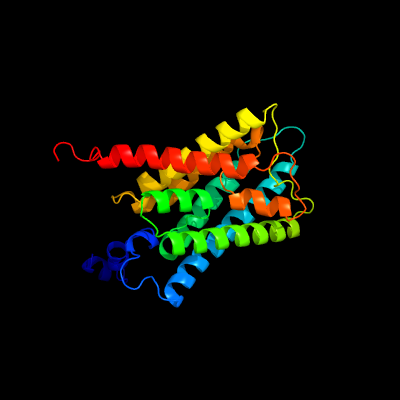 | 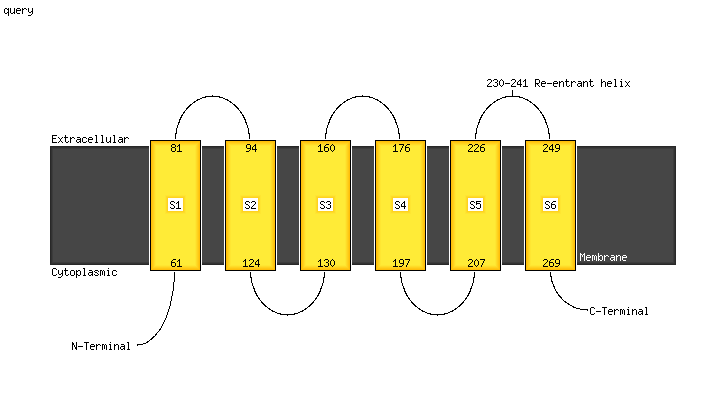 |
| [c2w2eA](http://www.sbg.bio.ic.ac.uk/phyre2/phyre2_output/d87407f37957e931/summary.html#c2w2eA_) | PaNIP5-1b | >PaLWMG0302726000.01.P01 | 100 | 82 | 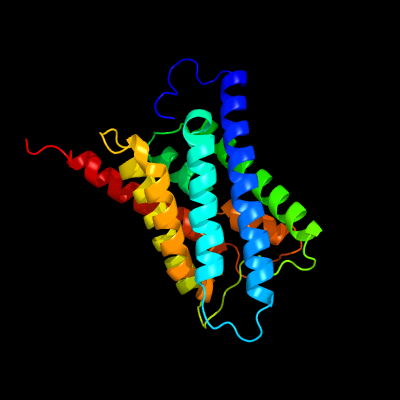 | 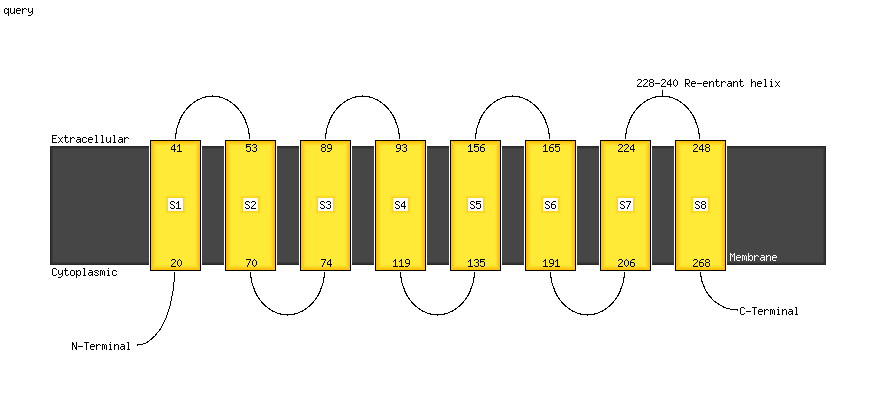 |
| [c2w2eA](http://www.sbg.bio.ic.ac.uk/phyre2/phyre2_output/0a12c9cb2b69d21e/summary.html#c2w2eA_) | PaNIP6-1 | >PaLWMG0303063200.01.P01 | 100 | 80 | 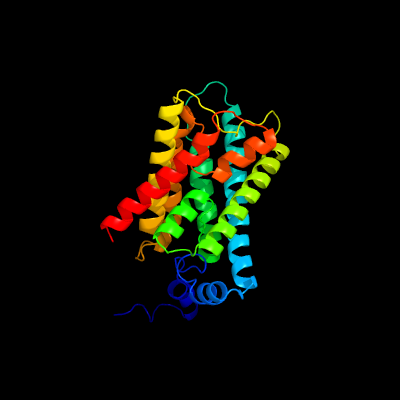 | 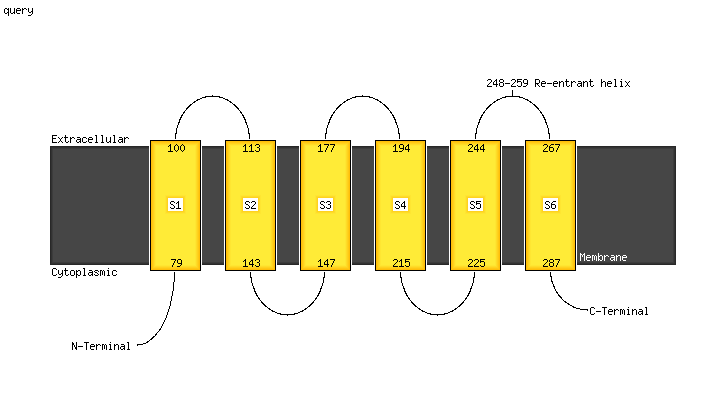 |
| [c2w2eA](http://www.sbg.bio.ic.ac.uk/phyre2/phyre2_output/41f1789285a9dd40/summary.html#c2w2eA_) | PaNIP7-1 | >PaLWMG0100384100.01.P01 | 100 | 84 | 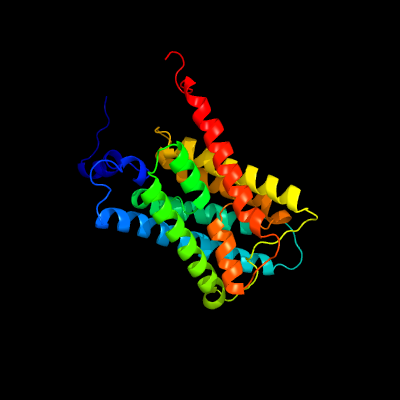 | 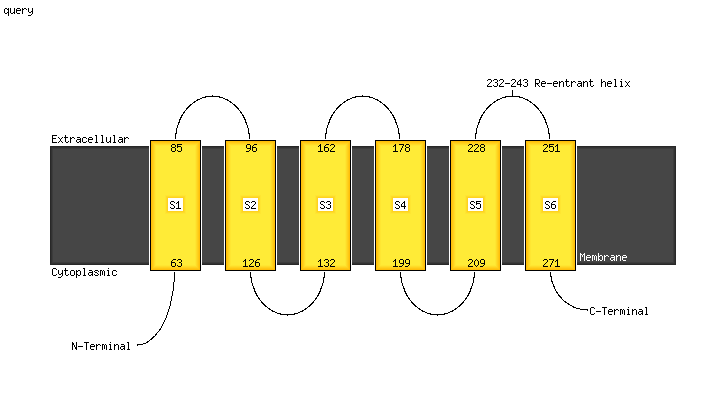 |
| [c5i32A](http://www.sbg.bio.ic.ac.uk/phyre2/phyre2_output/19d87ceafb741db2/summary.html#c5i32A_) | PaTIP1-1 | >PaLWMG0705957400.01.P01 | 100 | 93 | 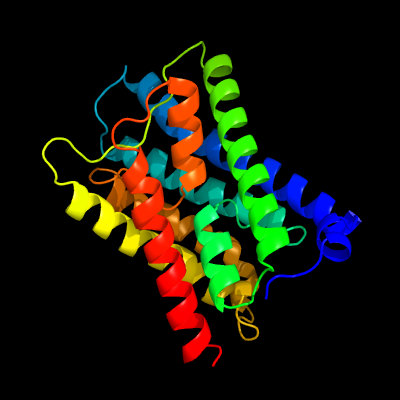 | 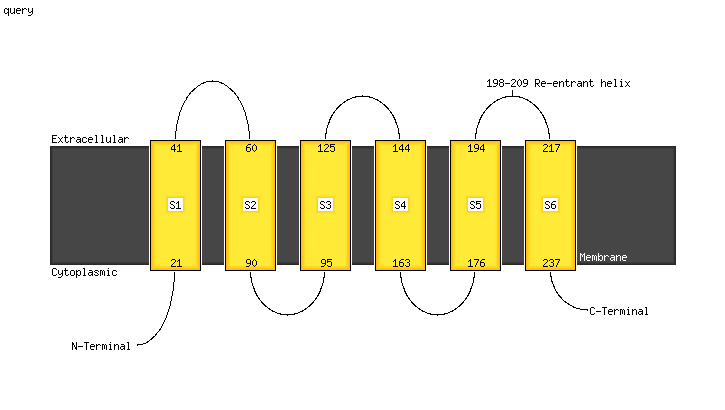 |
| [c5i32A](http://www.sbg.bio.ic.ac.uk/phyre2/phyre2_output/50448b16d136ce63/summary.html#c5i32A_) | PaTIP1-2 | >PaLWMG0604986400.01.P01 | 100 | 93 | 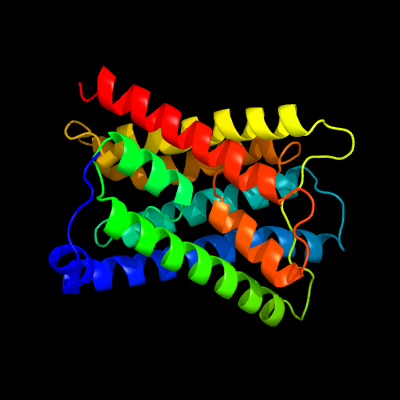 | 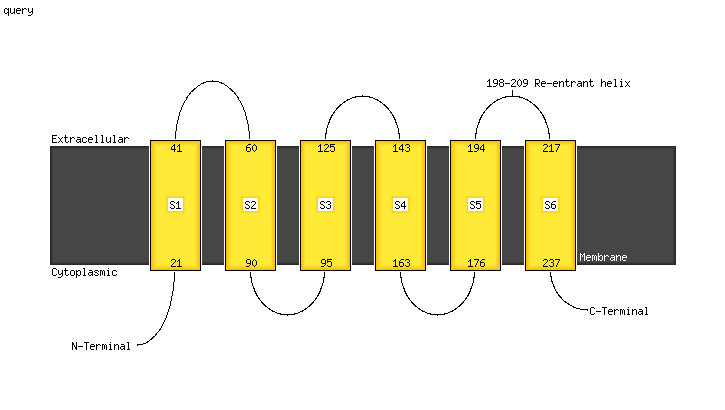 |
| [c5i32A](http://www.sbg.bio.ic.ac.uk/phyre2/phyre2_output/066618b57fb8b4ec/summary.html#c5i32A_) | PaTIP1-3 | >PaLWMG0202209500.01.P01 | 100 | 93 | 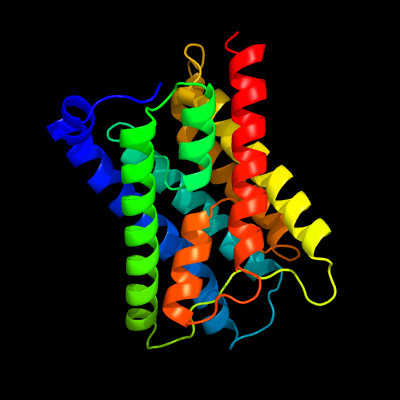 | 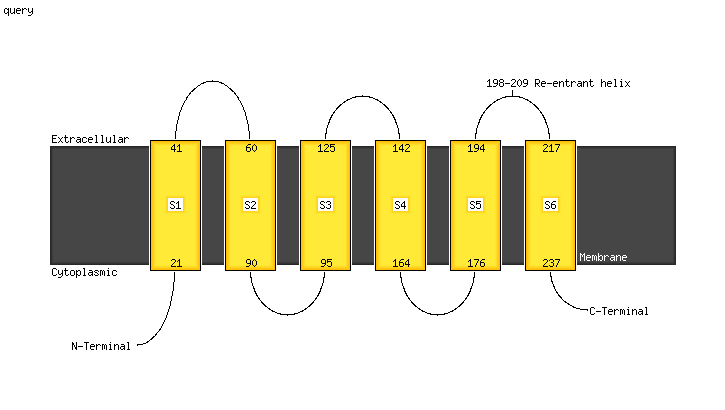 |
| [c5i32A](http://www.sbg.bio.ic.ac.uk/phyre2/phyre2_output/0ed7bb099a7a8df0/summary.html#c5i32A_) | PaTIP2-1 | >PaLWMG0303130000.01.P01 | 100 | 95 | 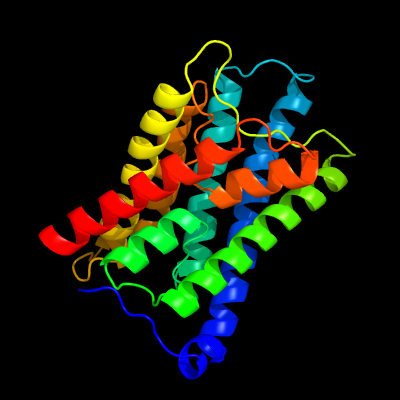 | 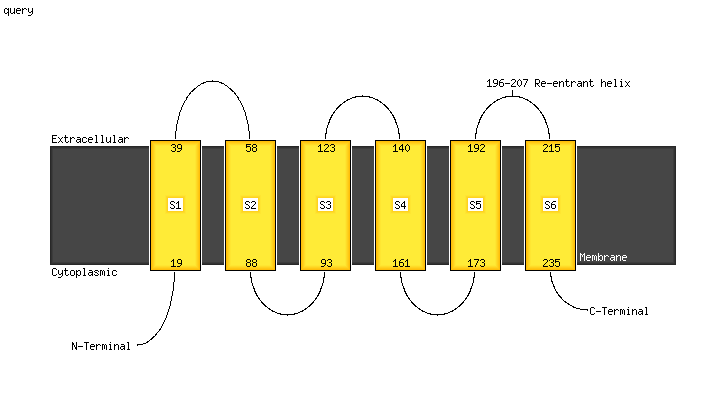 |
| [c5i32A](http://www.sbg.bio.ic.ac.uk/phyre2/phyre2_output/403af3aa5ee292a3/summary.html#c5i32A_) | PaTIP2-2 | >PaLWMG0504292200.01.P01 | 100 | 98 | 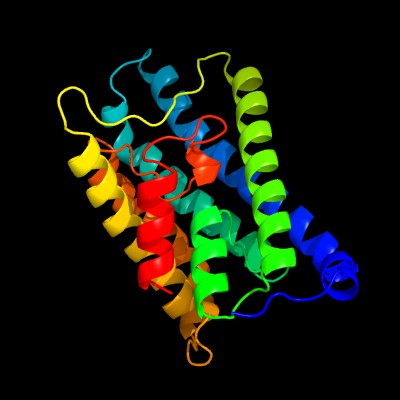 | 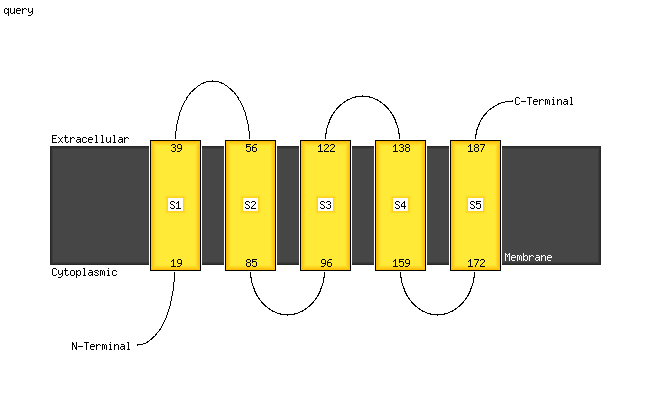 |
| [c5i32A](http://www.sbg.bio.ic.ac.uk/phyre2/phyre2_output/9e6e891c91f05396/summary.html#c5i32A_) | PaTIP2-3 | >PaLWMG0506397400.01.P01 | 100 | 68 | 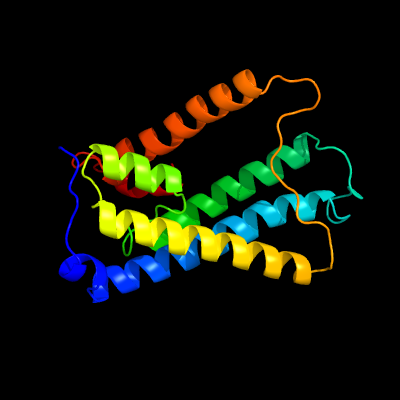 | 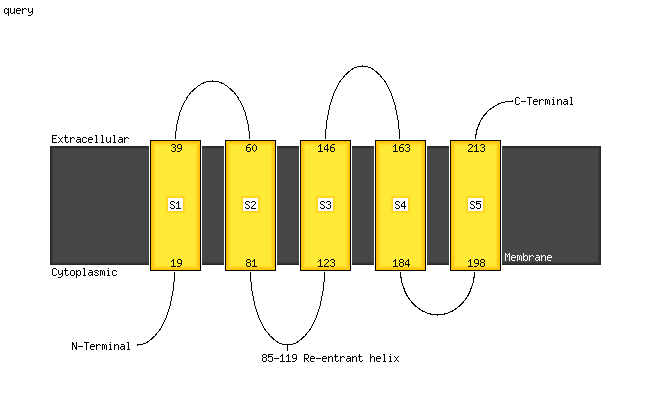 |
| [c5i32A](http://www.sbg.bio.ic.ac.uk/phyre2/phyre2_output/bc4a784d504378c7/summary.html#c5i32A_) | PaTIP3-2 | >PaLWMG0504762300.01.P01 | 100 | 90 | 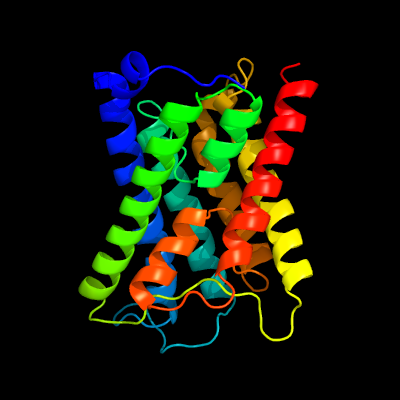 | 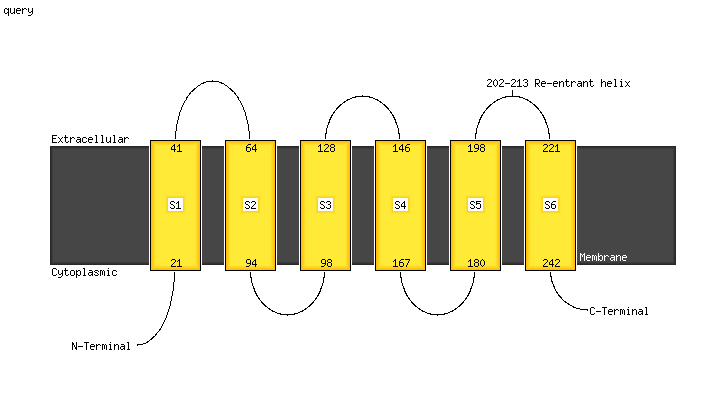 |
| [c5i32A](http://www.sbg.bio.ic.ac.uk/phyre2/phyre2_output/9c99f202108e7341/summary.html#c5i32A_) | PaTIP4-1 | >PaLWMG0101145200.01.P01 | 100 | 93 | 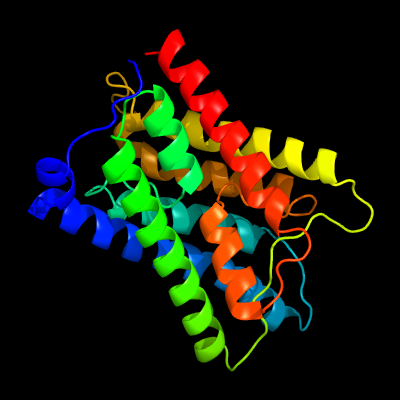 | 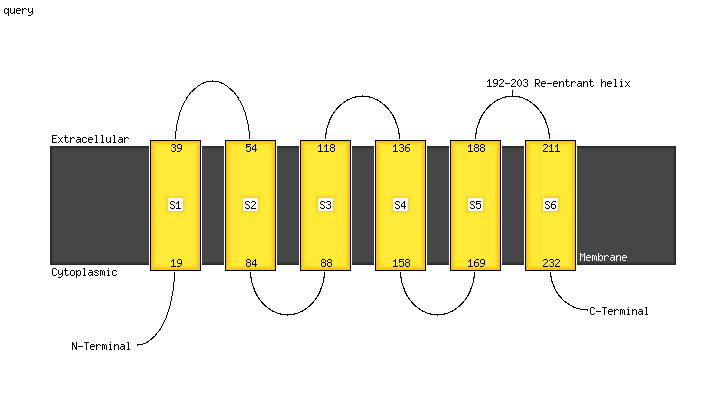 |
| [c5i32A](http://www.sbg.bio.ic.ac.uk/phyre2/phyre2_output/8352ac7d1390fe6e/summary.html#c5i32A_) | PaTIP5-1 | >PaLWMG0203269600.01.P01 | 100 | 91 | 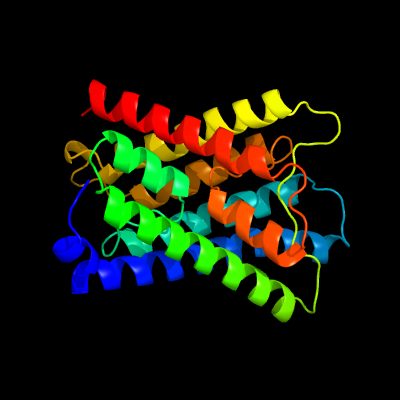 | 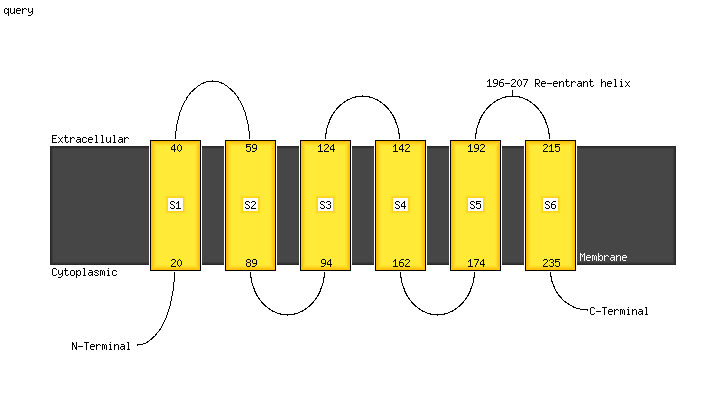 |
| [c5i32A](http://www.sbg.bio.ic.ac.uk/phyre2/phyre2_output/e04dd8c3404e62de/summary.html#c5i32A_) | PaTIP5-2 | >PaLWMG0506979500.01.P01 | 100 | 90 | 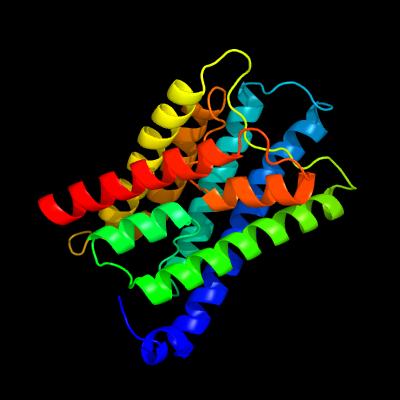 | 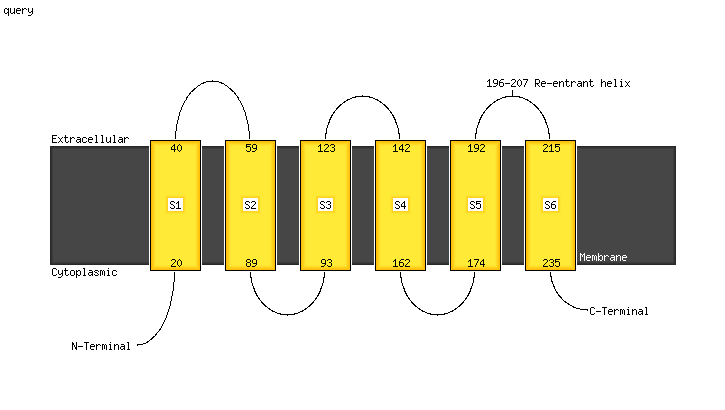 |
