# Supplementary material for: Genome-Wide Identification and Function of Aquaporin Genes During Dormancy and Sprouting Periods of Kernel-Using Apricot (Prunus armeniaca L.)
Source: Front Plant Sci. 2021 Oct 4;12:690040. doi: 10.3389/fpls.2021.690040 (PMC8520955; doi:10.3389/fpls.2021.690040)
Supplement: Supplementary Table 6 — Predicted functional partners of TIP, PIP, SIP, and NIP. Function of the top 10 highest scoring interacting proteins in each subfamily. [file Table_6.doc]

[**Table S6**](https://www.ncbi.nlm.nih.gov/pmc/articles/PMC3510131/figure/fig1/) **Predicted functional partners of TIP, PIP, SIP, and NIP. Function of the top 10 highest scoring interacting proteins in each subfamily.**

1. Predicted functional partners of PaTIP1-1:

| Name | Function | Method | | | | Score |
| --- | --- | --- | --- | --- | --- | --- |
|  |  | Cooccurence | Coexpression | Textmining | Homology |  |
| PIP2A | A member of the plasma membrane intrinsic protein subfamily PIP2. localizes to the plasma membrane and exhibits water transport activity in Xenopus oocyte. | √ | √ | √ | √ | 0.793 |
| DELTA-  TIP | Delta tonoplast integral protein; Aquaporin required to facilitate the transport of water from the vacuolar compartment to the cytoplasm. Does not promote glycerol permability. Its function is impaired by Hg(2+). | √ | √ | √ | √ | 0.759 |
| TIP2 | Tonoplast intrinsic protein 2; Water channel required to facilitate the transport of water across cell membrane. May be involved in the osmoregulation in plants under high osmotic stress such as under a high salt condition. | √ | √ | √ | √ | 0.733 |
| SIP1A | Small and basic intrinsic protein 1A; Belongs to a family of plant aquaporins. Similar to yeast and radish aquaporins. Located on ER; Belongs to the MIP/aquaporin (TC 1.A.8) family. SIP (TC 1.A.8.10) subfamily. |  |  | √ |  | 0.710 |
| SIP2;1 | Small and basic intrinsic protein 2;1; One of the Major Intrinsic Proteins(MIPs) which facilitate the passive transport of small molecules across membranes.Belongs to a family of plant aquaporins.Similar to yeast and radish aquaporins. Located on ER. |  |  | √ |  | 0.707 |
| RD28 | Aquaporin-like superfamily protein; A member of the plasma membrane intrinsic protein PIP2. functions as aquaporin and is involved in dessication; Belongs to the MIP/aquaporin (TC 1.A.8) family. PIP (TC 1.A.8.11) subfamily. | √ | √ | √ | √ | 0.697 |
| TIP1 | Ankyrin repeat family protein with DHHC zinc finger domain; Encodes a protein containing ankyrin and DHHC-CRD domain. Acts to restrict the size of the swelling that forms at the beginning of root hair cell growth, possibly by a mechanism that requires RHD1. Mutant displays defects in both root hair and pollen tube growth. |  |  | √ |  | 0.692 |
| TIP | NAC domain-containing protein 91; Transcription activator essential for the anti-viral defense called virus basal resistance response pathway. Not involved in HRT-mediated hypersensitive response (HR) and resistance to TCV. Binds DNA non specifically. Activated by proteolytic cleavage through regulated intramembrane proteolysis (RIP) (By similarity). |  |  | √ |  | 0.644 |
| SIP1;2 | Aquaporin-like superfamily protein; Belongs to a family of plant aquaporins.Similar to yeast and radish aquaporins. Located on ER; Belongs to the MIP/aquaporin (TC 1.A.8) family. SIP (TC 1.A.8.10) subfamily. |  |  | √ |  | 0.621 |
| TSPO | AtTSPO is related to the bacterial outer membrane tryptophan-rich sensory protein (TspO) and the mammalian mitochondrial 18 kDa Translocator Protein (18 kDa TSPO), members of the TspO/MBR domain-containing membrane proteins. Mainly detected in dry seeds, but can be induced in vegetative tissues by osmotic or salt stress or abscisic acid treatment. Located in endoplasmic reticulum and the Golgi stacks; Belongs to the TspO/BZRP family. |  |  |  |  | 0.561 |

1. Predicted functional partners of PaPIP2-1:

| Name | Function | Method | | | | Score |
| --- | --- | --- | --- | --- | --- | --- |
|  |  | Cooccurence | Coexpression | Textmining | Homology |  |
| ATHATPLC1G | Phosphatidylinositol-specific phospholipase C is induced to a significant extent under various environmental stresses, such as dehydration, salinity, and low temperature. May play a role in secondary ABA response. |  |  | √ |  | 0.899 |
| PIP1A | A member of the plasma membrane intrinsic protein subfamily PIP1. localizes to the plasma membrane and exhibits water transport activity in Xenopus oocyte. expressed ubiquitously and protein level decreases slightly during leaf development; Belongs to the MIP/aquaporin (TC 1.A.8) family. PIP (TC 1.A.8.11) subfamily. | √ | √ | √ | √ | 0.864 |
| PIP1B | A member of the plasma membrane intrinsic protein subfamily PIP1. localizes to the plasma membrane and exhibits water transport activity in Xenopus oocyte. expressed ubiquitously and protein level decreases slightly during leaf development; Belongs to the MIP/aquaporin (TC 1.A.8) family. | √ | √ | √ | √ | 0.838 |
| GAMMA-TIP | Gamma tonoplast intrinsic protein; Water channel required to facilitate the transport of water, diffusion of amino acids and/or peptides from the vacuolar compartment to the cytoplasm. Does not promote glycerol permeability. May play a role in the control of cell turgor and cell expansion. Its function is impaired by Hg(2+). May be involved in a vesicle-based metabolite routing through or between pre-vacuolar compartments and the central vacuole. Transports urea in yeast cells in a pH-independent manner. Transports H(2)O(2) in yeast cells. | √ | √ | √ | √ | 0.793 |
| PLDBETA1 | Phospholipase D beta 1; Hydrolyzes glycerol-phospholipids at the terminal phosphodiesteric bond to generate phosphatidic acids (PA). Plays an important role in various cellular processes, including phytohormone action, vesicular trafficking, secretion, cytoskeletal arrangement, meiosis, tumor promotion, pathogenesis, membrane deterioration and senescence. Involved in regulating stomatal movement and plant-water status. Can use phosphatidylserine (PS) and phosphatidylethanolamine (PE) as substrates only in the presence of PIP2.. |  |  | √ |  | 0.776 |
| TIP2 | Tonoplast intrinsic protein 2; Water channel required to facilitate the transport of water across cell membrane. May be involved in the osmoregulation in plants under high osmotic stress such as under a high salt condition. Transports urea in yeast cells in a pH-independent manner. Transports H(2)O(2) in yeast cells. | √ | √ | √ | √ | 0.774 |
| PLDALPHA1 | Phospholipase D alpha 1; Hydrolyzes glycerol-phospholipids at the terminal phosphodiesteric bond to generate phosphatidic acids (PA). Plays an important role in various cellular processes, including phytohormone action and response to stress, characterized by acidification of the cell. Involved in wound induction of jasmonic acid. May be involved in membrane lipid remodeling. Probably involved in freezing tolerance by modulating the cold-responsive genes and accumulation of osmolytes. |  |  | √ |  | 0.771 |
| PLDP1 | Phospholipase D zeta 1; Hydrolyzes glycerol-phospholipids at the terminal phosphodiesteric bond to generate phosphatidic acids (PA). Phosphatidylcholine-selective. Regulates root- hair morphogenesis. Contributes to the supply of inorganic phosphorus for cell metabolism and diacylglycerol moieties for galactolipid synthesis in phosphorus-starved roots. Involved in root elongation during phosphate limitation. |  |  | √ |  | 0.764 |
| PLDALPHA2 | Phospholipase D alpha 2; Hydrolyzes glycerol-phospholipids at the terminal phosphodiesteric bond to generate phosphatidic acids (PA). Plays an important role in various cellular processes, including phytohormone action and response to stress, characterized by acidification of the cell. |  |  | √ |  | 0.763 |
| PLDP2 | Phospholipase D zeta 2; Encodes a member of the PXPH-PLD subfamily of phospholipase D proteins. Regulates vesicle trafficking. Required for auxin transport and distribution and hence auxin responses. This subfamily is novel structurally different from the majority of plant PLDs by having phox homology (PX) and pleckstrin homology (PH) domains. Involved regulating root development in response to nutrient limitation. Plays a major role in phosphatidic acid production during phosphate deprivation. Induced upon Pi starvation in both shoots and roots. |  |  | √ |  | 0.749 |

1. Predicted functional partners of PaSIP1-3:

| Name | Function | Method | Score |
| --- | --- | --- | --- |
|  |  | Textmining |  |
| NIP4;1 | NOD26-like intrinsic protein 4;1; Potential aquaporin, which may facilitate the transport of water and small neutral solutes across cell membranes. | √ | 0.865 |
| NIP2;1 | NOD26-like intrinsic protein 2;1; Low water transport activity in yeast cells; Belongs to the MIP/aquaporin (TC 1.A.8) family. NIP (TC 1.A.8.12) subfamily | √ | 0.814 |
| NIP1;2 | NOD26-like intrinsic protein 1;2; Encodes an aquaporin homolog. Functions in arsenite transport and tolerance.When expressed in yeast cells can conduct hydrogen peroxide into those cells; Belongs to the MIP/aquaporin (TC 1.A.8) family. NIP (TC 1.A.8.12) subfamily | √ | 0.809 |
| NLM1 | NOD26-like major intrinsic protein 1; An aquaporin whose expression level is reduced by ABA, NaCl, dark, and dessication. is expressed at relatively low levels under normal conditions. Also functions in arsenite transport and tolerance | √ | 0.784 |
| NIP6;1 | NOD26-like intrinsic protein 6;1; Encodes a protein with boron transporter activity. It helps to preferentially direct boron to young developing tissues in the shoot, such as immature leaves, under low boron conditions. This boron channel appears to be impermeable to water, unlike the closely related NIP5;1 boron transporter. This protein also allows the transport of glycerol, urea, and formimide but not larger uncharged solutes such as arabitol and sucrose when it is expressed heterologously; Belongs to the MIP/aquaporin (TC 1.A.8) family. NIP (TC 1.A.8.12) subfamily | √ | 0.769 |
| PIP1B | A member of the plasma membrane intrinsic protein subfamily PIP1. localizes to the plasma membrane and exhibits water transport activity in Xenopus oocyte. expressed ubiquitously and protein level decreases slightly during leaf development. | √ | 0.768 |
| PIP2;5 | Plasma membrane intrinsic protein 2;5; Aquaporins facilitate the transport of water and small neutral solutes across cell membrane. | √ | 0.739 |
| GAMMA-TIP | Gamma tonoplast intrinsic protein; Water channel required to facilitate the transport of water, diffusion of amino acids and/or peptides from the vacuolar compartment to the cytoplasm. Does not promote glycerol permeability. May play a role in the control of cell turgor and cell expansion. Its function is impaired by Hg(2+). May be involved in a vesicle-based metabolite routing through or between pre-vacuolar compartments and the central vacuole. Transports urea in yeast cells in a pH-independent manner. | √ | 0.710 |
| PIP2B | Plasma membrane intrinsic protein 2; Water channel required to facilitate the transport of water across cell membrane. Plays an predominant role in root water uptake process in conditions of reduced transpiration, and in osmotic fluid transport. Its function is impaired by Hg(2+). Inhibited by cytosolic acidosis which occurs during anoxia in roots; Belongs to the MIP/aquaporin (TC 1.A.8) family. | √ | 0.698 |
| RD28 | Aquaporin-like superfamily protein; A member of the plasma membrane intrinsic protein PIP2. functions as aquaporin and is involved in dessication; Belongs to the MIP/aquaporin (TC 1.A.8) family. | √ | 0.695 |

(4) Predicted functional partners of PaNIP6-1:

| Name | Function | Method | | Score |
| --- | --- | --- | --- | --- |
|  |  | Coexpression | Textmining |  |
| BOR1 | HCO3- transporter family; Boron transporter. Protein accumulates in shoots and roots under conditions of boron deficiency and is degraded within several hours of restoring boron supply. Localized to the plasma membrane under B limitation, and to the cytoplasm after B application before degradation. Protein is transferred via the endosomes to the vacuole for degradation. Localized to the inner plasma membrane domain in the columella, lateral root cap, epidermis, and endodermis in the root tip region, and in the epidermis and endodermis in the elongation zone. | √ | √ | 0.951 |
| BOR4 | HCO3- transporter family; Efflux-type boron transporter polarly localized in roots. Boron is essential for maintaining the integrity of plants cell walls; Belongs to the anion exchanger (TC 2.A.31.3) family. | √ | √ | 0.836 |
| NodGS | Glutamate-ammonia ligases;catalytics;glutamate-ammonia ligases; Its function is described as glutamate-ammonia ligase activity, catalytic activity; Involved in nitrogen compound metabolic process, N-terminal protein myristoylation, nitrogen fixation, metabolic process, glutamine biosynthetic process; Expressed in 23 plant structures; Expressed during 13 growth stages; Contains the following InterPro domains: Glutamine synthetase, catalytic domain (InterPro:IPR008146), Glutamine synthetase, beta-Grasp (InterPro:IPR008147), Glutamine synthetase/guanido kinase, catalytic domain, etc. |  | √ | 0.835 |
| ACR3 | ACT domain-containing protein ACR3; May bind amino acids. |  | √ | 0.793 |
| SIP1A | Small and basic intrinsic protein 1A; Belongs to a family of plant aquaporins. Similar to yeast and radish aquaporins. Located on ER; Belongs to the MIP/aquaporin (TC 1.A.8) family. |  | √ | 0.769 |
| FPS1 | Farnesyl pyrophosphate synthase 1, mitochondrial; Catalyzes the sequential condensation of isopentenyl pyrophosphate with the allylic pyrophosphates, dimethylallyl pyrophosphate, and then with the resultant geranylpyrophosphate to the ultimate product farnesyl pyrophosphate. |  | √ | 0.738 |
| SIP2;1 | Small and basic intrinsic protein 2;1; One of the Major Intrinsic Proteins(MIPs) which facilitate the passive transport of small molecules across membranes.Belongs to a family of plant aquaporins.Similar to yeast and radish aquaporins. |  | √ | 0.708 |
| WRKY6 | WRKY family transcription factor; Transcription factor involved in the control of processes related to senescence and pathogen defense. Interacts specifically with the W box (5’- (T)TGAC[CT]-3’), a frequently occurring elicitor-responsive cis- acting element. Activates the transcription of the SIRK gene and represses its own expression and that of the WRKY42 genes. Modulates phosphate homeostasis and Pi translocation by regulating PHO1 expression; Belongs to the WRKY group II-b family. |  | √ | 0.677 |
| DUR3 | Solute:sodium symporters;urea transmembrane transporters; High-affinity urea-proton symporter involved in the active transport of urea across the plasma membrane into root cells. May play an important role in urea uptake by plant cells at low external urea concentrations. |  | √ | 0.627 |
| SIP1;2 | Aquaporin-like superfamily protein; Belongs to a family of plant aquaporins.Similar to yeast and radish aquaporins. Located on ER; Belongs to the MIP/aquaporin (TC 1.A.8) family. |  | √ | 0.625 |
