# Supplementary material for: Genome-Wide Identification and Function of Aquaporin Genes During Dormancy and Sprouting Periods of Kernel-Using Apricot (Prunus armeniaca L.)
Source: Front Plant Sci. 2021 Oct 4;12:690040. doi: 10.3389/fpls.2021.690040 (PMC8520955; doi:10.3389/fpls.2021.690040)
Supplement: Supplementary Table 8 — Amino acid sequences of AQP genes in poplar. [file Table_8.docx]

**Table S8 Amino acid sequences of *AQP* genes in poplar*.***

>PtPIP1;1 Potri.010G191900

MEGKEEDVRLGANKFNERQPLGTAAQSQDDKDYKEPPPAPLFEPSELTSWSFYRAGIAEFMATFLFLYITVLTVMGVFKDTTKCTTVGIQGIAWAFGGMIFALVYCTAGISGGHINPAVTFGLFLARKLSLTRAVFYMLMQCLGAICGAGVVKGFYGKKNYELLNGGANMVSPGYTKGDGLGAEIVGTFVLVYTVFSATDAKRSARDSHVPILAPLPIGFAVFLVHLATIPITGTGINPARSLGAAIIFNKDKAWDDHWIFWVGPFIGAALAALYHQVVIRAIPFKK

>PtPIP1;2 Potri.008G065600

MEGKEEDVRLGANRFNERQPIGTAAQSLDDKDYKEPPPAPLFEPGELTSWSFYRAGIAEFMATFLFLYITVLTVMGVVKDQTKCTTVGIQGIAWAFGGMIFALVYCTAGISGGHINPAVTFGLFLARKLSLTRAVFYMVMQCLGAICGAGVVKGFYGKTNYELHNGGANMVAHGYTKGDGLGAEIVGTFILVYTVFSATDAKRSARDSHVPILAPLPIGFAVFLVHLATIPITGTGINPARSLGAAIIFNKDSAWDDHWIFWVGPFIGAALAALYHQVVIRAIPFKK

>PtPIP1;3 Potri.003G128600

MEGKEEDVKLGANKFSERQPIGTSAQTDKDYKEAPPAPLFEPGELKSWSFYRAGIAEFIATFLFLYITVLTVMGVTKPGTSKCSTVGIQGIAWAFGGMIFALVYCTAGISGGHINPAVTFGLFLARKLSLTRAVFYIIMQCLGAICGAGVVKGLQGSHNYELQGGGANVVNHGYTKGDGLGAEIVGTFVLVYTVFSATDAKRNARDSHVPILAPLPIGFAVFLVHLATIPITGTGINPARSLGAAIIFNKDHAWDDHWIFWVGPFIGAALAAVYHQIVIRAIPFKSRA

>PtPIP1;4 Potri.006G098100

MEEGEEDVKVGANRYGEGQPIGTAAQTQHGKDYTEPPPAPLYQPGEWLSWSFYRAGIAEFVATFLFLYITVLTVMGVARSSTKCSTVGIQGIAWAFGGMIFVLVYCTAGISGGHINPAVTFGLLLARKLTLTRAVFYMIMQCLGAICGAGVVKGFQKSPYEILGGGANTVSTGYSKGSGLGVEILGTFVLVYTVFSATDAKRSARDSHVPVLAPLPIGFAVFLVHLATIPITGTGINPARSLGAALIYNKDKAWDDHWIFWVGPFIGAALASLYHQIVIRAIPFKSK

>PtPIP1;5 Potri.016G113300

MEGREEDVRVGANKYGERQPIGTAAQAQDVKDYTDPPPAPLFEPGELSSWSFYRAGIAEFVATFLFLYITVLTVMGVAKSPTKCSTVGIQGIAWAFGGMIFALVYCTAGISGGHINPAVTFGLLLARKLSLTRAVFYMLMQCLGAICGAAVVKAFQKSQYEMLGGGANTVSTGYAKGSGLGAEIVGTFVLVYTVFSATDAKRNARDSHVPILAPLPIGFAVFLVHLATIPITGTGINPARSLGAALIYNKDQAWDDHWIFWVGPFIGAALASLYHQIVIRAIPFKSK

>PtPIP2;1 Potri.009G136600

MSKDVIEEGQTHTKDYVDPPPAPLFDVGELKLWSFFRALIAEFIATLLFLYVTVATVIGHKKNQDACGGVGLLGIAWAFGGMIFILVYCTAGISGGHINPAVTFGLLLARKVSLIRAVGYMVAQCLGAVCGVGLVKAFMKPYYNSLGGGANMVAPGYSTGTAVGAEIIGTFVLVYTVFSATDPKRSARDSHIPVLAPLPIGFAVFMVHLATIPITGTGINPARSFGAAVIINDKKAWDDHWIFWVGPFVGALAAAAYHQYILRAGAIKALGSFRSHPTN

>PtPIP2;2 Potri.004G176300

MSKEVSEVGQTHGKDYVDPPPAPLLDLGELKLWSFYRALIAEFIATLLFLYVTVATVIGHKSNKDPCDGVGLLGIAWAFGGMIFILVYCTAGISGGHINPAVTFGLFLARKVSLIRAVAYMVAQCLGAICGVGLVKAFMKKNYNSLGGGANTVAMGYNTGTALGAEIIGTFVLVYTVFSATDPKRSARDSHVPVLAPLPIGFAVFMVHLATIPITGTGINPARSFGAAVIFNNEKAWDDHWIFWVGPFVGALAAAAYHQYILRAAAIKALGSFRSNPAN

>PtPIP2;3 Potri.010G222700

MAKDMEVAEAGSFSAKDYHDPPPAPLFDAKELTKWSFYRALIAEFIATLLFLYITVLTVIGYKSQIDGSADSCGGVGILGIAWAFGGMIFVLVYCTAGISGGHINPAVTFGLFLARKVSLIRAVMYMVAQCLGAICGVGLVKAFQKSYYKKYGGGANTLADGFSTGTGLGAEIIGTFVLVYTVFSATDPKRSARDSHVPVLAPLPIGFAVFMVHLATIPITGTGINPARSLGAAVIYNQDKAWDGHWIFWVGPFAGAAIAAFYHQFILRAGAVKALGSFRSAQRF

>PtPIP2;4 Potri.008G039600

MAKDTEVAEAGSFSAKDYQDPPPAPLIDAEELTKWSFYRALIAEFIATMLFLYITVLTVIGYKSQIDGNADPCGGVGILGIAWAFGGMIFVLVYCTAGISGGHINPAVTFGLFLARKVSLIRAVMYMVAQCAGAICGVGLVKAFQKSYYTKYNGGANVLADGYSTGTGLGAEIIGTFVLVYTVFSATDPKRSARDSHVPVLAPLPIGFAVFMVHLATIPITGTGINPARSFGAAVIYNNKKAWHDQWIFWAGPFIGAAIAAFYHQFILRAGAIKALGSFRSNPNV

>PtPIP2;5 Potri.006G128000

MGKDIEVGGEFSAKDYHDPPPAPLIDAEELTQWSLYRAIIAEFIATLLFLYITVLTVIGYKSQTDTTKNSDACGGVGILGIAWAFGGMIFVLVYCTAGISGGHINPAVTFGLFLARKVSLVRAVLYMVAQCLGAICGCGLVKAFQKSYYTKYGGGVNELATGFSKGTGLGAEIIGTFVLVYTVFSATDPKRNARDSHVPVLAPLPIGFAVFMVHLATIPITGTGINPARSFGAAVIYNEDKAWDDHWIFWVGPFIGAAIAALYHQYVLRAAAVKALGSFRSSSNI

>PtPIP2;6 Potri.006G128200

MGKDVEVRGEFIAKDYHDPPPAPLIDAEELTQWSLYRAIIAEFIATLLFLYITVLTVIGYKSQTDTTKNSDACGGVGILGIAWAFGGMIFVLVYCTAGISGGHINPAVTFGLFLARKVSLVRAVLYMVAQCLGAICGCGLVKAFQKSYYTKYGGGANELATGFSKGTGLGAEIIGTFVLVYTVFSATDPKRNARDSHVPVLAPLPIGFAVFMVHLATIPITGTGINPARSFGAAVIYNKDKAWDDHWIFWVGPFIGAAIAALYHQYVLRAAAVKALGSFRSSSNI

>PtPIP2;7 Potri.016G089500

MGKDIEVGGEFSAKDYHDPPPAPLIDAEEITQWSFYRAIIAEFVATLLFLYITVLTVIGYKSQTDVNKNGDECGGVGILGIAWAFGGMIFILVYCTAGISGGHINPAVTFGLFLARKVSLVRAILYMVAQCLGAICGCGLVKAFQKSYYTNYGGGANGLANGYSKGTGLGAEIIGTFVLVYTVFSATDPKRNARDSHVPVLAPLPIGFAVFMVHLATIPITGTGINPARSFGAAVIFNKEKAWDDHWIFWVGPFIGAAIAALYHQFILRAAAVKSLGSFRSSPNI

>PtPIP2;8 Potri.009G013900

MAKDIEVAEHGETVKDYQDPPPAPLIDAEELGQWSFYRALIAEFIATLLFLYVTVLTVIGYKSQTDPDKGLDACGGVGILGIAWAFGGMIFVLVYCTAGISGGHINPAVTFGLFLARKVSLIRAVLYMVAQCLGAICGCGLVKAFQKSYYNHYGGGANELQEGYNKGTGLGAEIIGTFVLVYTVFSATDPKRNARDSHVPVLAPLPIGFAVFMVHLATIPITGTGINPARSFGAAVIFNQSKAWDDHWLFWVGPFIGAAIAAFYHQFILRAAAIKALGSFRSNA

>PtPIP2;9 Potri.005G109300

MSTGGKDYRDPPPAPLLDMEELKQWSFYRALIAEFVATFLFLYIGVGTVVGYKGVHNNLCDGAGYLGVAWAFGGMIFVLVYCTAGISGGHINPAVTFGLFVARKVSLIRAVAYMMAQCLGAMLGVWMVMILTGIHYDQAGGAVNVVAPGYSKGTALGAEIIGTFVLVYTVLAATDPKRMARDSHVPVLAPLPIGFAVFVVHLALIPITGTGINPARSLGAAVVKNAKEIWDDHWIFWVGPFVGALAAAVYHQYILGSGAAKALASFRSNPTS

>PtPIP2;10 Potri.005G109200

MSSEERNIERQHGRDYHDPPPAPLLDMGELKQWSFYRAAIAEFIATFLFLFFSVSTVVNYKEPNYTDQCSRVGHLGIAWANGGMIFVLVYCTSGISGGHLNPAVTFGMLVARKMSLIRAAAYMLAQCLGAILGHLFVFLFMYADEQQSSVGVVNVVSRNYSKGAGLGAEFIGTFVLVYTVFSATDPKRNARDSHVPVLAPLPIGFAVFVVHLATIPITGTGINPARSLATNLLHRSTAEAMDDLWIFWVGPFLGALAAAVYHKYVLRAGAVKTLKSFRALGSFGSQPPV

>PtTIP1;1 Potri.001G235300

MPITSIAFGSPAEAGQPDALRAALAEFISMLIFVFAGEGSGMAFNKLTDNGSSTPAGLVAASLAHAFALFVAVSVGANISGGHVNPAVTFGAFIGGHITFIRSLLYWVAQCLGSVVACLLLKLATGGQETSAFALSSGVGAWNAVVFEIVMTFGLVYTVYATAVDPKKGDIGIIAPIAIGFIVGANILAGGAFDGASMNPAVSFGPAVVSWTWDSHWVYWLGPFVGSAIAAIVYEVIFINPSTHEQLPSTDF

>PtTIP1;2 Potri.009G027200

MAITSIAFGSPAEVGQSDALKAALAEFISMLIFVFAGEGSGMAFNKLTDDGSSTPAGLVAASLAHAFALFVAVSVGANISGGHVNPAVTFGAFLGGHITFIRSILYWVAQCLGSVVACLLLKLATGGLETSAFSLSSGVGVWNAVVFEIVMTFGLVYTVYATAVDPKRGDIGIIAPIAIGFIVGANILAGGAFDGASMNPAVSFGPAVVSWTWDNHWVYWLGPFVGSAIAAIVYEVCFISPTTHEQLTSSDF

>PtTIP1;3 Potri.010G209900

MPINRIAFGTPREASHPDALRAALAEFISMLIFVFAGSGSGMAFNKLTDNASTTPSGLVAAALAHAFALFVAVSVGANISGGHVNPAVTFGALIGGNITLLRSILYWIAQLLGSVVACLLLKFATGGLETPAFGLSSGVGAWNALVFEIVMTFGLVYTVYATAVDPKKGNLGIIAPIAIGFIVGANILAGGAFDGASMNPAVSFGPAVVSWTWTNHWVYWLGPFIGAAIAALVYDNIFIGSGGHEPLPTNDF

>PtTIP1;4 Potri.008G050700

MPINRIAVGTPGEASHPDSLRAALAEFISTLIFVFAGSGSGMAFNKLTDSASTTPAGLVAAALAHAFALFVAVSVGANISGGHVNPAVTFGALIGGNITLLRSILYWIAQLLGSVVACLLLKFSTGGLETPAFGLSSGVGAWNAVVFEIVMTFGLVYTVYATAVDPKKGNLGIIAPIAIGFIVGANILAGGAFDGASMNPAVSFGPAVVSWTWTNHWVYWLGPFIGAGIAALVYDNIFIGSGGHEPLPTNDF

>PtTIP1;5 Potri.016G098200

MPIRNIAVGHYRETTQPDALKAALAEFISTLIFVFAGEGSGMAFSKLTDGASNTPAGLIAAAIAHAFALFVAVSVGANISGGHVNPAVTFGAFIGGNITLFRGILYWIAQLLGSTVACLLLKFVTGGLETSAFALSTGVGVWNAFVLEIVMTFGLVYTVYATAIDPKKGNLGIIAPIAIGFIVGANILVGGAFDGASMNPAVSFGPALVSWSWTNHWVYWAGPLVGGGLAGLIYELFFIGFGTHEQLPTTDY

>PtTIP1;6 Potri.006G121700

MPIRNIAVGHYHEATQPDALRAALAEFISTLIFVFAGEGSGMAFAKLTDGAANTPAGLIAAAIAHAFALFVAVSVGANISGGHVNPAVTFGAFIGGNITLLRGILYWIAQLLGSTVACLLLKFTTGGLETSAFALSSGVGVWNAFVLEIVMTFGLVYTVYATAVDPKKGNLGIIAPIAIGFIVGANILAGGAFDGASMNPAVSFGPALVSWTWTNHWVYWAGPLIGGGLAGLIYEFFFIGFGNHEQLPTADY

>PtTIP1;7 Potri.009G005400

MPNLIVIDRIAIGTVAADFHPNAFKAALAEFISTLIFVFAGQGSTMAYNKLTSNAPTSPAGLIAVALAHAFGLFVAVATSANISGGHCNPAVTFGAFLGGNITLLRGILYWIAQLLGSTVACLLLKFATHYMTVSVFTLSSGVSVWNAFVFEIVMTFALVYTVYATAIDAKKGDVGVIAPLAIGFVLGANILAGGAFEGAALNPAVPFGPALVSWNWHHHWVYWAGPLIGGGLAGVVYELIFISHTHEPLAVVEY

>PtTIP1;8 Potri.004G216500

MRNFIIIERITIGRVEDDFHSNAFKAALAEFISTLIFVFAGQGSTMAYNKLTSNAPTSPAGLIAVALAHAFGLFVGVAVSANISGGHVNPAVTFGAFIGGNISLLRGILYWIAQLLGSTVACLLLKYTTHHMTVSVFTLSPGVTVWNAFVFEIVMTFALVYTVYATAIDPKKGDVGVIAPLAIGFVLGANILVGGAFEGAALNPAVPFGPALVSWNWYHHWVYWAGPLIGGGLAGIVYELIFMSHSTHEPLPGGEF

>PtTIP2;1 Potri.001G186700

MAGIAFGRFDDSFSLGSFKAYLAEFISTLLFVFAGVGSAMAYNKLTGDAALDPAGLVAIAVCHGFALFVAVSVGANISGGHVNPAVTFGLALGGQITILTGIFYWIAQLLGSIVACYLLKVATGGLAVPIHSVAAGVGAIEGVVMEIIITFALVYTVYATAADPKKGSLGTIAPIAIGFIVGANILAAGPFSGGSMNPARSFGPAVASGDFHDNWIYWAGPLVGGGIAGLIYGNVFITDHTPLSGDF

>PtTIP2;2 Potri.003G050900

MARIAFGRFNDSFSLGSLKAYLAEFISTLLFVFAGVGSAMAYNKLTGDAALDPAGLVAIAVCHGFALFVAVAVGANISGGHVNPAVTLGLALGGQMTILTGIFYWIAQLLGSIVACYLLKVVTGGLAVPIHSVAAGVGAIEGVVMEIIITFALVYTVYATAADPKKGSLGTIAPIAIGFIVGANILAAGPFSGGSMNPARSFGPAVASGDFHDNWIYWVGPLIGGGLAGLIYGNLYITDHSPSSYEF

>PtTIP2;3 Potri.003G077800

MAKIAFGSLGDSFSLASIKAYLSEFIATLLFVFAGVGSAIAYSKLTTDAALDPPGLVAVAVAHAFALFVGVSIAANISGGHLNPAVTFGLAIGGNITFLTGLLYWIAQCLGSIVACLLLKVVTSAEGIPTHGVASGMSAIEGVVMEIVITFALVYTVYATAADPKKGSLGIIAPIAIGFIVGANILAAGPFSGGSMNPARSFGPAVVSGDFSQNWIYWLGPLVGGGLAGLVYGGIFIGSYAPAPVSEDYA

>PtTIP2;4 Potri.001G157000

MVKIAFGSLGDSFSVGSLKAYLSEFIATLLFVFAGVGSAIAYSKLTTDAALDPPGLVAVAVAHAFALFVGVSIAANISGGHLNPAVTFGLAIGGNITILTGLLYWIAQCLGSIAACLLLKFATSAESIPTHGVASGMSAVEGVVMEIVITFALVYTVYATAADPKKGSIGIIAPIAIGFIVGANILAAGPFSGGSMNPARSFGPAVVSGDFSQNWIYWLGPLIGGGLAGLVYGDIFIGSYTAAPVSEDYA

>PtTIP3;1 Potri.018G152100

MPRRYAFGKADEATRPDAMRAALAELVSTFIFVFAGEGSILALDKLYKGTGPPASGLLVVALAHALALFSAVASSINISGGHVNPAVTFGSLVGGRISVIRAVSYWVAQLLGSIFAALLLRLVTNGMIPAGFHVQSEVGEVHGLLLEMALTFGLVYTVYATAIDPKRGSLGIIAPLAIGFVVGANILVGGPFDGASMNPARAFGPALVGWRWRNHWIYWVGPFLGGGLAALIYEYIVISAEPVAHHTHQHQPLAPEDY

>PtTIP3;2 Potri.017G154800

MPRRHAFGRADEATHPDSMRAALAEFVSTFVFVFAGEGSVLALDKLYKETGPLASGLVVVALAHALALFSAVASSINISGGHVNPAVTFGSLVGGRISVIRAVYYWVAQLLGSIVAALLLRLVTNGMRPVGFHVQSGVGEVHGLLMEMALTFGVVYTVYATALDPKRGSLGIIAPLAIGFIVGANILVGGPFDGASMNPARAFGPALIGWRWRNHWIYWVGPFLGGGLAALIYEYIVIPTEPVPRHAHQHQPLAPEDY

>PtTIP4;1 Potri.006G239700

MTKIALGSRHEAAQPDCLKALVVEFVTTFLFVFAGVGSAMAADKLTGDALLGLFVVAVAHAFVVAVMISAGHISGGHLNPAVTIGLLFGGHITVVRSILYWIDQLLASTAACFLLKYLTGGLATSVHTLASGMDYLQGVVWEIVLTFSLLFTVYATIVDPKKGSIDGLGPMLTGFVVGANILAGGAFSGASMNPARSFGPALVSWDWTDHWVYWVGPLIGGGLAGFIYENFFITRSHRPLPSEEEPF

>PtTIP5;1 Potri.003G108500

MASTSLTARFKQSVTPASLRAYLAEFISTFFYVFAVVGSAMASRKLLPDAAAVPSSLVIVAIANAFALSSAVYIAANASGGHVNPAVTFGMAVGGRINVPTALFYWISQMLASVMACIFLKVATVGQHVPTNTIAEEMTGFGASLLEGVMAFGLVYTVYAAGDPRRGSLGAIGPLAVGLTAGANVLAAGPFSGGSMNPACAFGSAVIAGRLKNQAVYWVGPLIGAAVAGLLYDNVVFPTEAPDSLRGVSDDVGV

>PtTIP5;2 Potri.001G124800

MAPTSLTARFQQSVTPASLRAYLAEFISTFFYVFAVVGSAMASRKLLPDAAADPSSLVIVAIANAFALSSAVYIAANASGGHVNPAVTFGMAVGGHINVPTALFYWISQLLASVMASIFLKVTTVGQHVPTYTIAEEMTGFGASLLEGVMTFGLVYTVYAAGDPRRSSLGAIGPLAVGLMAGANVLAAGPFSGGSMNPACAFGSAVIAGKFKNQAVYWVGPLIGASVAGLLYDNVVFPTQAPDSGRRGVSEGVGV

>PtNIP1;1 Potri.004G063000

MAEIDGTNGNGNHGGVVLDIKDNYPSSSSIKEVSVLNFYVPFMQKLVAEIAGTYFLIFAGCSSVAVNLNFDKVVTLPGISITWGLAVMVLVYSVGHISGAHFNPAVTLAFATCKRFPWKQVPAYVACQVIGATLAAGTIRLLFQGDQDHFTGTMPAGSNLQSFVVEFIITFYLMFIISGVATDNRAIGELAGLAVGSTVLLNVMFAGPISGASMNPARSLGPAIVSHQYKGLWIYIVSPILGAQAGAWVYNLIRYTDKPLREITKSASFLNGKESS

>PtNIP1;2 Potri.011G072100

MADIDGTGSNGNHGGVVLDIKDDHPPTSSNLTKEDSDLYFSVPFMQKLVAEIVGTYFLIFAGCSSVAVNLNFEKVVTLPGISIVWGLAVMVLVYSLGHISGAHFNPAVTLAFATCKRFPWKQVPAYISCQVIGSTLAAGTIRLIFQGKQDHFTGTMPAGSDLQSFVVEFIITFYLMFIISGVATDNRAIGELAGLAVGSTVLLNVMFAGPISGASMNPARSLGPAMVSHEYRGIWIYVVSPILGAQAGAWVYNLIRYTDKPLREITKSASFLQSKGRF

>PtNIP2;1 Potri.017G083300

MATVDQEMNISVESSRFHFVKLFREHYPSGFLRKVVAEVIATYLLVFVTCGAAAISASDEHKVSKLGASVAGGLIVTVMIYAVGHISGAHMNPAVTTAFAAVLNFPWKQVPFYAAAQLTGAISASFTLKVLLHPIRNVGTTSPSGTAVQALIMEIVVTFSMMFITSAVATDTKAVGELAGIAVGSAVCITSILAGPVSGGSMNPARTLGPAIASRYFKGVWVYLLGPVTGTLLGAWSYNLIRVTDKPVQAIPRRFSFGSRRTRAIDEQSPSMGPLDAF

>PtNIP3;1 Potri.002G097000

MSSSNSITEPSPKFQLPTRRSIMAEAKAASPAPEWLSTRNAALSNFQKIVAELMGTYILVFVGCGAALTDKVQRLNMLGIAIVWGAVLMAAIYALGHVSGAHFNPAVSIALAVVRKFSWKEVPMYILAQVLGSTLASLTLRMLFHEQGNIQPIVNQYSDPTSDLEAIVWEFIITFILMFTICGVATDPRASKDLSGVAIGGAVMFNAMIAGPITGASMNPARSLGPALVSGVYKNLWVYIVSPILGAMAAAAVYSVLRVPEPAKPEDTNKSTYNNLNLHADP

>PtNIP4;1 Potri.010G112900

MPWNNEFGDDTEGGKKTESSDEDSPPETTVQIIQKIIAEMIGTFFLIFMGCGSVVVNQMYGSVTFPGVCVVWGLIVMVMVYSVGHISGAHFNPAVTVTFAIFRHFPYKQVPLYIAAQLLGSLLASGTLSLLFSVTDEAYFGTIPVGPDIRSFVTEIIISFLLMFVISGVATDNRAIGELAGIAVGMTIMLNVFVAGPVSGASMNPARSLGPAIVMRQFKGIWVYIVGPPIGTILGALCYNIIRFTDKPLREITKTASFLKSKN

>PtNIP4;2 Potri.017G128200

MARKSDGIESQEITSMEEGLATPTDPKENGKFDCCTSPAAVTITQKLIAEVIGTYFVIFAGCGSVAVNNIYGSVTFPGVCVTWGLIVMVMIYSLGHISGAHFNPAVTIAFAIFRRFPSWQVPLYIIAQLMGSILASGTLALALDVTPEAFFGTVPVGSDGQSLVLEIIISFLLMFVISGVSTDDRAVGDLAGIAVGMTILLNVFVAGPVSGASMNPARSIGPAVVKHQFKGLWVYIVGPIIGAIAGAFACNLIRWTDKPLGELTKVGSFIKSGSKNYAS

>PtNIP5;1 Potri.001G455000

MPESEAGTPAVSAPNTPGTPGGPLFTGLRVDSLSYSDRKIMPKCKCLPVTAPTWGQPHTCFLDFPAPDVSLTRKLGAEFVGTFILIFAATAGPIVNQKYNNAETLIGNAACAGLAVMIIILSTGHISGAHLNPSLTIAFAALRHFPWVQVPAYIAAQVSASICASFALKGVFHPFMSGGVTVPSVSTGQAFALEFLITFNLLFVVTAVATDTRAVGELAGIAVGATVMLNILVAGPSSGGSMNPVRSLGPAVAAGTYKDIWIYLVAPTLGALVGAATYTAVKLREEEADPPRQVRSFRR

>PtNIP5;2 Potri.011G146900

MPGPEEAGTPTVTAPNTPGTPGGPLFTGLRVDSLSYSDRKIMPKCKCLPVTAPNWGQPHTCFLDIPSPDVSLTRKLGAEFVGTFILIFMATAGPIVNQKYDHAETLIGNAACAGLAVMIIILSTGHISGAHLNPSLTIAFAALRHFPWVQVPAYIAAQVSASICASFALKGVFHPFMSGGVTVPSVSTGQAFALEFFITFNLLFVVTAVATDTRAVGELAGIAVGATVMLNILVAGPSTGGSMNPVRTLGPAIAAGNYKKIWIYLVAPTLGAVVGAGAYTLVKLRDDETDPPRPVRSFRR

>PtNIP6;1 Potri.003G180900

MDNAEVPSVPSTPATPGTPGAPLFGGFKGERGVHGRKSLLRSCKCFSVEEWAMEEGRLPPVSCSLPPPPVSLARKVGAEFIGTLILIFAGTATAIVNQKTQGSETLVGLAASSGLAVMIVILATGHISGAHLNPSITIAFAALKHFPWKHVPVYIGAQVLASLCAAFALKGIFHPVMGGGVTVPSGGYGQAFALEFITSFILMFVVTAVATDTRAVGELAGIAVGATVMLNIFIAGETTGASMNPVRTLGPAIAVNNYKAIWIYLTAPILGALCGAGTYSAVKLPEEDGDSNEKTSAARSFRR

>PtNIP6;2 Potri.001G046800

MDTEEVPSAPSTPATPGTPGAPLFGGFKGERGVHGRKSLLRSCKCFGVEEWAMEEGRLPPVSCSLPPPPVSLARKLGAEFMGTLILIFAGTATAIVNQKTQGSEALIGLAASTGLAAMIVILSTGHISGAHLNPSITIAFAALKHFPWKHVPVYIGAQVLASLCAAFALKVIFHPMMGGGVTVPSGGHGQAFALEFIISFILMFVVTAVATDTRAVGELAGIAVGATVMLNILIAGETTGASMNPVRTLGPAIAANNYKAIWVYLTAPILGALCGAGTYSAVKLPEEDGDTNEKTSATRSFRR

>PtNIP7;1 Potri.008G203300

MKHLLEEITSAHVPKTAVLPPASSSSSSTDDQEMDSNSMPMKRHIFIKKSSFCSFLHGMDLNPARMVLAEMVGTFLLLFCVCGIVACTQILRGEVGLMEYASVAGLTIIVVIFSIGSISGAHVNPAVTIAFATFGHFPWSKVPLYILAQTVGSVSATYVGSSVYGVKTELMTTRPAIGCSSAFWVEFMATFMLMFLAASLTSQSRSIGPLSGFLYGIAIGLAVLITGPVSGGSLNPARSLGPAIVSWDFKDIWVYITAPTIGAVAGALMFHLLRIRPQACSANSSPDDDLLVHSIAFTES

>PtXIP1;1 Potri.009G128100

MWRATLTELVATTLTTCLLFTLTTSIISCLESTTAEPKFLIPFAIIVIAFFFLLTTVPLSGGHMSPVFTFIAALEGVITPVRALFYMSAQCVGSIVAYLVIKSVMDKNAEEKYSLGGCMIDGNGEGISPTNAFILEFSCTFIVLFVGVTVAFDKRRCKELGLQMVCGILAGAMALAFFVSISVTGRAGYAGVGLNPARCLGPSLLKGGRLWYGHWVFWVGPFVACIVYYGFTLTLPTGTS

>PtXIP2;1 Potri.009G128500

MAGNAGVVQDEEIGYGGNKVQPFASTPRPSKTERGKRDSSALSRILGLDELVSLNVWRASLAEVFGTAVLVFAMDTIVISSYETQTKTPNLVMATLIAITIAILLLATFPISGGHINPAITLSAMFTGLITVSRAAIYILAQCIGAILGALALKAVVNSTIEQTFSLGGCTLEIVAPGPSGPVAIGLETGQALWLEIICTFVFLFSSIYIAFDRRQAIALGRVVFCSIIGLVVGLLVFISTTVTATKGYAGVGMNPARCLGPALVRGGHLWKGHWVFWVGPVVASVAFSLYTKMIPREHLLEQNQNREAFHVVVSVNVIIHLLPFHGHNVV

>PtXIP3;1 Potri.009G128300

MAEALKNEGGKTKQITWREILGLEDLLSLTVWRASVAELLGTAVLVFALDTIVISTIQTGTNMPNLILSTLVAIIITILLLATFPISGGHINPIITFAAFLTGLISLSKTFIYILAQCVGAIFGALALKAVVNSEIEKTYSLGGCTLTIVAPGPHGPTVIGLETNQALWLEIICGFVFLFASVWMAFDHRQAQGIGRVGVFIIGGIVLGLLVFVSTTVTTTKGYAGAGLNPARCLGPAIVRGGHLWNGHWVFWVGPAVACVAFAVYTKIIPRQLAHTIE

>PtXIP3;2 Potri.009G128000

MAEALKNEGGKTKQITLREILGLEDLLSLTVWRASVAELLGTAVLVFALDTIVISTIQTGTNMPNLILSTLVAIIITILLLATFPISGGHINPIITFAAFLTGLISLSKTFIYILAQCVGAIFGALALKAVVNSEIEKTYSLGGCTLTIVAPGPHGPTVIGLETNQALWLEIICGFVFLFASVWMAFDHRQAQGIGRVRVLIIVGIVLGLLVFVSTTVTATKGYAGAGLNPARCLGPAIVRGGHLWNGHWVFWVGPAIACVAFAVYTKIIPRQLAHTIE

>PtXIP3;3 Potri.009G127900

MAGYTEGDEENLFRANKIQRFATTATTPTAEVVKNEKRMKKQKSTKLSEILGLEDLVSLTVWRASVAELIGTAVLVFTLDTIVISTIRIETKIPNLILSILAAIIITILILATFPISGGHINPLVTFAALLTGLVSLSKAIIYILAQCVGGIFGALALKAVVNREIQQTFSLGGCTLTVVAPGPDGQTVIGLETSQALWLEIICGFVFLFASVWMAFDQRQAKALGRVNVFIIIGIVVGLLVYISTTVTATKGYAGAGLNPARCLGPAIVRGGHLWDGHWVFWVGPGIACVLFALYTKLIPPQLSHTIE

>PtXIP3;4 Potri.004G167000

MAGYPGSTVEDEESLYSGKKPQPSATTPMAKVVQNEGGIQKKKSPTLREILGLEDLFSLTTWRASVAELLGTAVLVFALDTIVISTIQTQTKTPNLILSTLVAIIVTILLLATYPISGGHINPIVTFAALLTGLISISKAFIYILAQCVGGIVGALALKAVVNSEIERTFSLGGCTLTVVAPGPEGPTVVGLETGQALWLEIICGFVFLFASVWMAFDHRQAKGLGRVNVLIIVGIVLGLLVYVSTTVTATKGYAGAGLNPARCLGPAIVRGGHLWNGHWVFWVGPAIACVAFAIYTKVIPSQLSHTIE

>PtSIP1;1 Potri.013G053400

MGAVKAAIGDAVFTFMWVFVSSMFGLFTNVIVTALGLQTLVWAPVLANASLIFAFVFLFNFLGEFLGGATFNPTGTASFYAAGVGGDSLFSMALRFPAQAAGSVGGSLAILEVMPLQYKHMLGGPTLQVDLQTGGLAEGVLTFLMTFAVLVIILKGPRSSLVQAWFLATVTVTLVSAGSTYTGPSMNPAFAFGWAYVNKWHNTWEQLYVYWICPFIGAILAAWVFRVVFPPPAPKQKKT

>PtSIP1;2 Potri.019G030900

MGAIKAASGDAVLTFMWVFVSSMFGLFTNLIVTALGLQTLVWAPLVITTFIVFTFVFLFNLIGEALGGASFNPTGTASFYAAGVGGDTLFSMALRFPAQAAGAVGGALAIMEVMPVQYKHMLGGPTLQVDLHTGGLAEGVLTFLMSFAVLVIILKGPRNPLVQTLFLAIATITLVVAGSTYTGPSMNPANAFGWAYVRKWHNTWEQLYVYWICPFIGAILASWVFRAVFPPPAPKQKKA

>PtSIP1;3 Potri.002G227500

MGAIKGAIVDGILTAMWVFSVPLLGVFSSIIATYVGVEAMSIAGLFISINVAALFMLTFSLIGAAFGGASFNPATTITLYIAGLKPDASLLSMALRFPVQAAGGVGGAMAIRGVMPKHYRHVLKGGPSLRVDLHTGAIAEGVLTFLICLTLHFLLLKGPKNVVLKVWFLAVATVGLVMAGGKYTGPSMNPANAYGWAYLGNRHTTWDFFYVYWICPFIGAILAAFVSKFLFKAAPIKEKKA

>PtSIP1;4 Potri.014G154400

MGAIKGAIVDGILTCMWVFSVPLLGVFSSIIATYVGVEAMSIAGLFITINVAALFMLTFSLIGAACGGASFNPATTITLYTAGLKPDASLMSMALRFPVQAAGGVAGAMAITEVMPKQYRYVLRGGPSLKVDLHTGAIAEGVLTFLICLALHFVLLKGPKNFVLKVWLLAVATVGLVMAGGKYTGPSMNPANAYGWAYLSNRHTTWDFFYVYWICPFIGATLAALISKFLFKAPPIKDKKA

>PtSIP2;1 Potri.016G024900

MVSKTRLILSDFVVSLMWVWSGSLIKIFVFKVLGMGHDSRGEFLKNSLSIMNMFLFAFLGKFTKGGAYNPLTILSSAISGDFSQFLFTIGARIPAQVIGSITGVRLFIDTFPEIGLGPRLTVDIHKGALTEGLLTFAIVTISLGLARKIPGSFFMKTWISSVSKLSLHILGSDLTGGCMNPASVMGWAYARGDHITKEHILVYWLAPIEGTLLAVWTFKLLFRPQKQDEKEKLKGKTE

>PtSIP2;2 Potri.006G027200

MVSKTRLIVSDFIVSIIWVWNGALIKMFVFKVLQMGHDSRGEFMRQSLTVVSLFFFAFLAKVTKGASFNPLAVLSSAISGDFSHFLFTIGTRIPAQVIGSITAVRLLIDTFPEIGRGPRLNVDIHKGALTEGLLAFGVVTISLGLARKIPGSFFMKTWISSISKLSLHILGSDLTGGCMNPASVMGWAYARGDHITKEHILVYWLAPIQGALLAAYTFKLLFRPQKQDEKEKLKGKTD
